# Optical Control of the Glucocorticoid Receptor Activity With a Photoswitchable Agonist

**DOI:** 10.1002/anie.9699968

**Published:** 2026-09-26

**Authors:** Zoe Sessions, Chloé Van Leene, Dorien Clarisse, George Keen, Agnė Šveistytė, Dinahlee Saturnino Guarino, Robert H. Mach, Karolien De Bosscher, Dirk Trauner

**Affiliations:** ^1^ Department of Chemistry University of Pennsylvania Philadelphia Pennsylvania USA; ^2^ VIB‐UGent Center for Medical Biotechnology (CMB) Ghent Belgium; ^3^ Department of Biomolecular Medicine Ghent University Ghent Belgium; ^4^ Cancer Research Institute Ghent (CRIG) Ghent Belgium; ^5^ Department of Systems Pharmacology and Translational Therapeutics Perelman School of Medicine University of Pennsylvania Philadelphia Pennsylvania USA; ^6^ Department of Radiology University of Pennsylvania Philadelphia Pennsylvania USA

**Keywords:** drug design, glucocorticoid, photoswitch, receptors

## Abstract

Glucocorticoids (GCs) are widely prescribed anti‐inflammatory agents whose clinical utility is limited by systemic side effects arising from broad glucocorticoid receptor (GR) activation. Optical control of GR pharmacology offers a strategy to modulate receptor signaling with spatial and temporal resolution. Here, we report **AzoCort1**, a prednisolone‐derived photopharmaceutical that enables light‐dependent tuning of GR activity in cell culture. Upon irradiation, **AzoCort1** exhibits low picomolar activity (EC_50_ = 11 pM) in a gene expression assay, representing a 36‐fold enhancement relative to its dark state (EC_50_ = 400 pM). Despite its high potency, **AzoCort1** is less efficacious in triggering glucocorticoid response element (GRE)‐driven activation compared to dexamethasone, while preserving maximal GR‐dependent repression of NF‐κB and AP‐1 signaling. These findings demonstrate light‐driven control of GR activity and separation of maximal GR‐mediated gene activation from gene repression.

## Graphical abstract

A photoswitchable glucocorticoid receptor agonist enables optical control of receptor activity, achieving picomolar potency and partial agonism that preferentially maintains anti‐inflammatory signaling over canonical transcriptional activation.

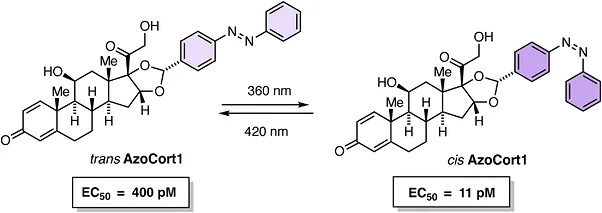

## 1 Introduction

Glucocorticoids (GCs) are central regulators of inflammation and metabolic homeostasis, acting through the glucocorticoid receptor (GR), a ligand‐activated nuclear receptor that functions as a transcription factor (TF) to regulate thousands of genes [1]. Upon ligand binding, GR translocates to the nucleus and modulates transcription through various mechanisms. GR‐mediated gene activation typically occurs via direct DNA binding to glucocorticoid response elements (GRE) in regulatory regions of target genes. Repression of genes controlled by pro‐inflammatory TFs such as NF‐κB and AP‐1 can arise from tethering of GR to these TFs or the binding of the GR to cryptic GREs within TF response elements [2, 3, 4, 5]. This dual mechanism underlies the anti‐inflammatory efficacy of widely prescribed glucocorticoids such as prednisone and dexamethasone. Since the first scalable syntheses of cortisone in the mid‐20th century, extensive structural diversification has produced numerous glucocorticoid derivatives for systemic, topical, and inhaled therapeutic applications.

Despite their efficacy, chronic glucocorticoid therapy frequently produces severe adverse effects that depend on dose, treatment duration, and route of administration. Systemic administration can lead to metabolic dysfunction, osteoporosis, adrenal suppression, and increased cardiovascular risk, while topical application can result in poor wound healing and skin thinning problems [6, 7]. These toxicities arise from the broad and sustained genomic activity of GR agonists, which cannot currently be confined in space or time. As a result, clinical use involves an unavoidable trade‐off between therapeutic benefit and systemic side effects. Strategies that enable spatiotemporal control of GR pharmacology are therefore highly desirable.

Photopharmacology offers an attractive approach to achieve such control by incorporating photoswitchable elements that modulate ligand conformation and bioactivity in response to defined wavelengths of light. This strategy enables external regulation of drug activity with spatial and temporal precision, without requiring chemical degradation, through the incorporation of light‐responsive motifs, such as azobenzenes [8]. The rational design of azobenzene‐containing ligands typically relies on either bioisosteric replacement (azologization) of key structural motifs or installation at positions predicted to be tolerated within the ligand‐binding site [9, 10].

Building on these principles, we report a series of photoswitchable glucocorticoids (**AzoCorts**) designed to enable optical modulation of glucocorticoid receptor signaling. The design of our **AzoCort** series was guided by structure–activity relationships of glucocorticoids substituted at C16α, such as dexamethasone and desisobutyryl ciclesonide (DBC) (Figure 1a). Hobson et al. demonstrated that the C16α/C17 diol present in the glucocorticoids 16α‐hydroxy prednisolone (16‐αHP), triamcinolone, and fluocinolone can undergo acetal formation with bulky linkers to afford antibody‐drug conjugates (ADCs) that retain glucocorticoid receptor binding and activity (Figure 1a) [11, 12]. These findings suggested that this position of the steroid scaffold tolerates structural modifications resembling photoswitchable azobenzenes. We therefore targeted the C16α/C17 diol for installation of an azobenzene photoswitch with the goal of preserving GR engagement while enabling photoisomer‐dependent modulation of activity, resulting in compounds such as **AzoCort1** (Figure 1b).

**FIGURE 1:**
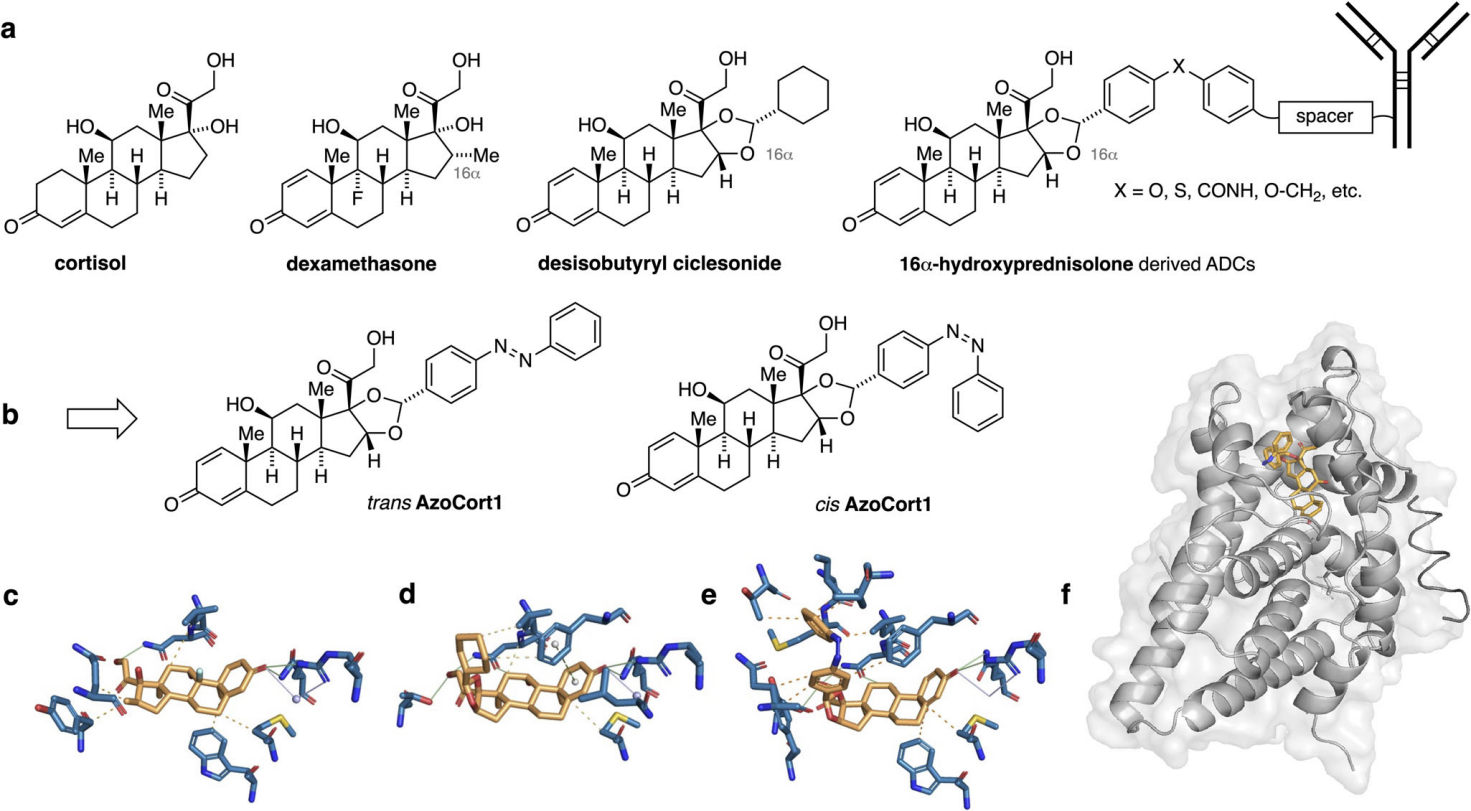
Design of **AzoCort1** and molecular docking of **AzoCort1** showing proposed interactions of glucocorticoids at key residues. (a) Structures of foundational glucocorticoids and derivatives at the C16α/17 diol, including desisobutyryl ciclesonide and antibody‐drug conjugates by Hobson et al. [11, 12, 13]. (b) Structures of **AzoCort1** with *cis* and *trans* photoisomers shown. (c–e) Proposed binding interactions of ligands within the GR‐LBD binding pocket, calculated by PLIP [14]; interacting residues are visible with orange lines depicting hydrophobic interactions, green lines depicting hydrogen bonding, and water bridging in grey. (c) Proposed binding interactions of dexamethasone from the reported crystal structure (PDB: 4UDC) of GR–LBD in complex with dexamethasone [15]. (d) Proposed binding interactions of desisobutyryl ciclesonide from the reported crystal structure (PDB: 4UDD) of GR–LBD in complex with desisobutyryl ciclesonide [16]. (e) Proposed binding interactions of **AzoCort1** from the molecular docking into the GR‐LBD (PDB: 4UDD) [16]. (f) View of the docking pose of *cis* **AzoCort1** in the GR–LBD determined with Schrödinger Maestro [17].

## 2 Results and Discussion

### 2.1 Design, Synthesis, and Photocharacterization of AzoCorts

Our design was further supported by the x‐ray crystal structure of the acetal desisobutyryl ciclesonide bound to the GR ligand binding domain (LBD) [16], which revealed a flexible pocket capable of accommodating substituents projecting from the C16α position (Figure 1d). It suggested that extension of the scaffold with an azobenzene photoswitch could be accommodated within the LBD, providing a handle for optical control. We first explored our hypothesis of photoisomer‐preferred binding in silico by 3D molecular docking using Schrödinger Maestro [17]. Following positive control re‐docking of DBC (PDB: 4UDD) [16], prednisolone docking gave a satisfactory glide score of −10.69. Docking of *cis* **AzoCort1** yielded a glide score of −11.58 (Figure 1f), an encouraging result compared to 16‐αHP (−11.26) and Dex (−11.15). Docking suggested that azobenzene incorporation would be allowed only in the *cis* photoisomer, as no *trans* photoisomer docked with a satisfactory score ( ≤ −6.0). Further, analysis of residue interactions using PLIP [14] provided a rationale for the improved binding score over the known steroids. Compared to the structure of dexamethasone‐bound GR (PDB: 4UDC [15], Figure 1c) and DBC‐bound GR (PDB: 4UDD [16], Figure 1d), *cis* **AzoCort1** docked into 4UDD is predicted to have the same hydrogen bonds as DBC [N564 (2), Q570, R611, T739], which is increased compared to Dex [N564 (1), Q570, and R611]. The *cis* **AzoCort1** is predicted to share the same water bridge as Dex, at R611. Furthermore, *cis* **AzoCort1** bound GR is predicted to have ten hydrophobic interactions within 4 Å (Figure 1e). This maintains all the hydrophobic interactions predicted both for DBC (4) and Dex (5), with four additional connections at T556, I559, M560, and I629 (Figure 1c–e) [18].

Encouraged by these calculations, we set out to synthesize **AzoCort1** through acid‐catalyzed condensation of 16‐αHP with an azobenzene aldehyde (Scheme 1a). This afforded a mixture of **AzoCort1** and its diastereomer **AzoCort1β**, as well as their respective photoisomers, which could be separated by HPLC. Analogously, we synthesized **AzoCort2** and **AzoCort3** from triamcinolone and fluocinolone, respectively (Scheme 1b). We hypothesized that the increased fluorination would increase binding affinity and thus could impact the sensitivity of the receptor to the conformational change induced by irradiation. Finally, we synthesized **AzoCort4**‐**6** from 16‐αHP with diversified photoswitch properties targeting a better photostationary state, fast relaxation, and the potential of additional hydrogen bonding.

**SCHEME 1:**
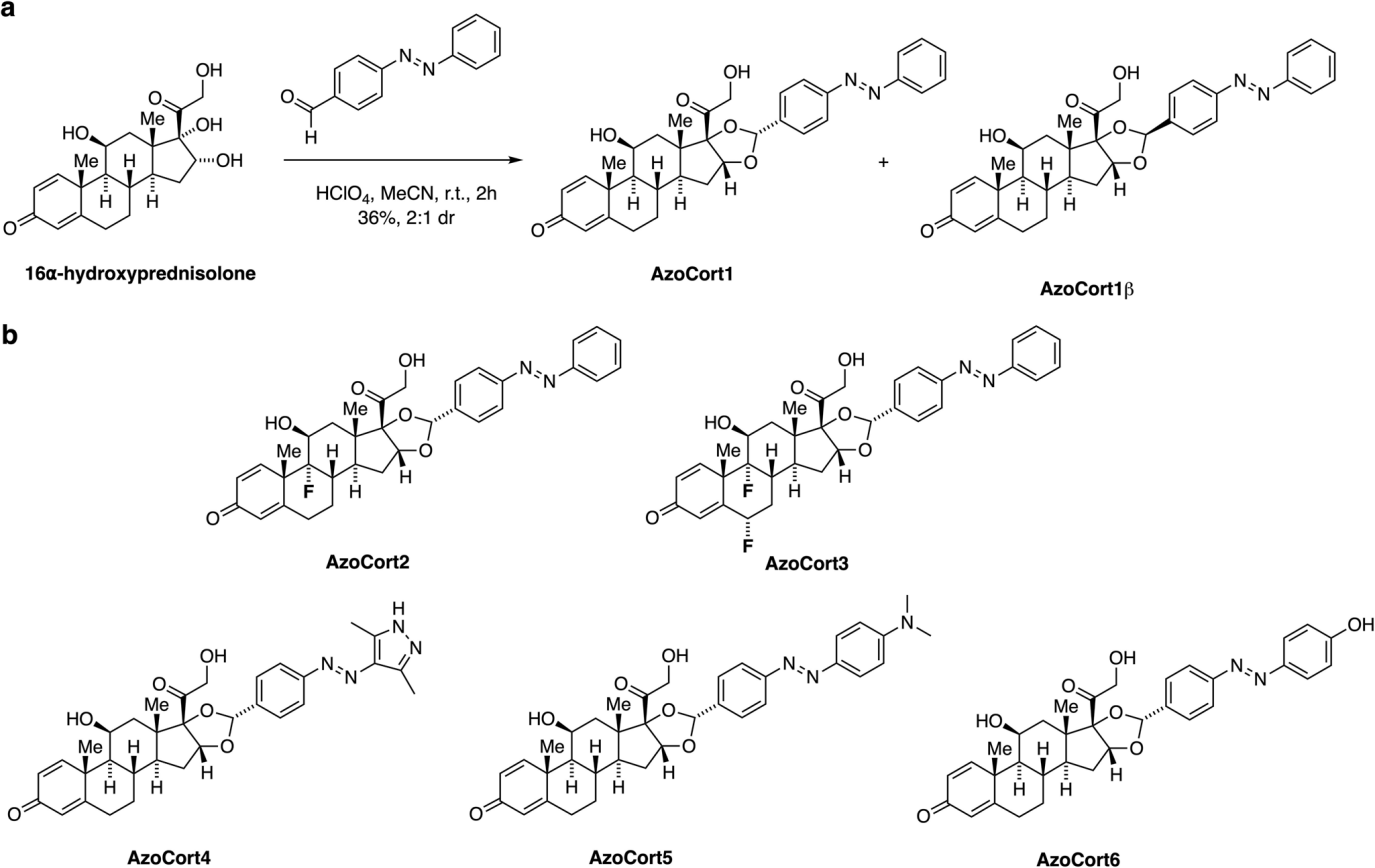
Synthesis of the AzoCorts. (a) Representative synthesis of **AzoCort1** and **AzoCort1β**, illustrating the introduction of the azobenzene moiety. (b) Chemical structures of the AzoCort derivatives (**AzoCort 2–6**) synthesized in this study.

Photophysical characterization of **AzoCort1** yielded optimal *cis* enrichment at 360 nm (PSS_360_ = 84% *cis*) and *trans* enrichment at 420 nm (PSS_420_ = 94% *trans*), a thermal *cis* half‐life of 33 h (DMSO, 37°C), and switching reversibility through at least six cycles (Figure 2). The photophysical properties of **AzoCort1**–**3** were similar, whereas the arylazopyrazole **AzoCort4** demonstrated excellent photostationary states in both directions (PSS_360_ = 96% *cis*, PSS_440_ = 97% *trans*). The electron‐rich azobenzene derivatives **AzoCort5** and **AzoCort6** were distinguished by very short thermal half‐lives and poor photostationary states (see Supporting Information).

**FIGURE 2:**
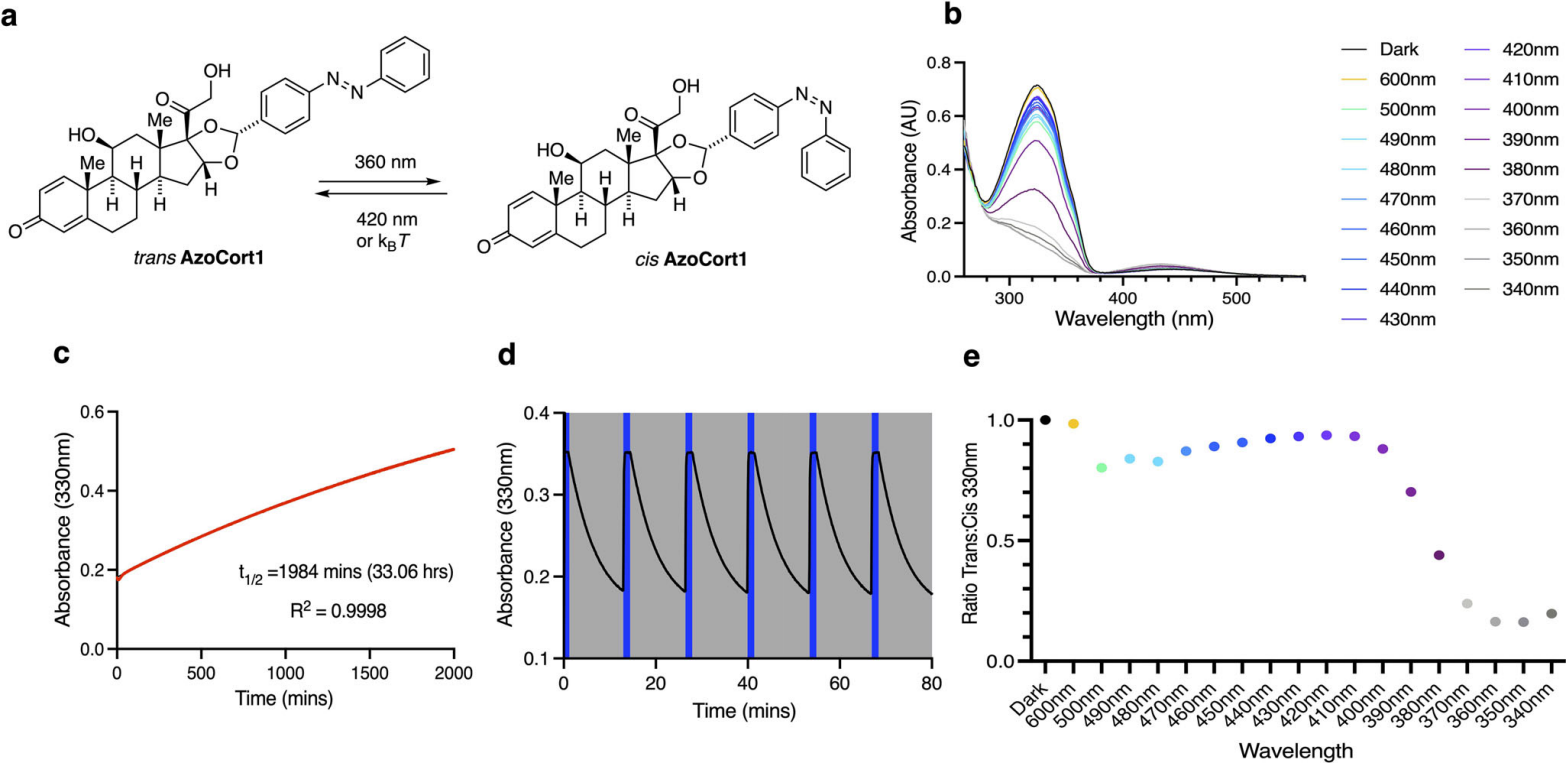
Photochemical characterization of **AzoCort1**. (a) Schematic photoswitching of **AzoCort1**. (b–e) Photophysical characterization of 50 µM **AzoCort1** in 100% DMSO. (b) UV–vis absorption spectra following irradiation at differing wavelengths. (c) Thermal relaxation at 37°C in DMSO and calculation of half‐life. (d) Photocycling between *trans* and *cis* enrichment of **AzoCort1**. (e) Relative *trans*:*cis* ratios extracted from the wavelength scan.

### 2.2 In Vitro Evaluation of AzoCort1

With **AzoCorts1–6** in hand, we set out to evaluate their light‐responsive activity on the GR. A previously described luciferase reporter assay [18] designed to specifically monitor GRE‐dependent transcriptional activation of endogenous GR upon ligand stimulation was used to evaluate transcriptional activity for all compounds. **AzoCort1** demonstrated the strongest light‐dependency via GRE luciferase assays and thus became the focus of our investigation [13, 19]. Varying light‐dependent transcriptional activity was observed with **AzoCort2**‐**6** (Figure S3 and Table S3), but none at the level of **AzoCort1**. Consistent with literature precedent, the β diastereomers at the acetal junction, such as **AzoCort1β**, were inactive [13, 19].

As observed in Figure 3, dark‐adapted **AzoCort1** demonstrated an EC_50_ value of 400 pM, which is 10‐fold lower compared to Dex. Upon irradiation with 360 nm light, a 36‐fold decrease was observed (EC_50_ 11 pM). As such, **AzoCort1** irradiated with 360 nm light was found to be >8000 times more potent than its precursor, 16‐αHP. The plateau of **AzoCort1**‐induced GRE‐driven gene expression was consistently capped between 33%–35% of Dex saturation values, classifying **AzoCort1** as a partial GR agonist. As demonstrated in the literature [20, 21, 22, 23, 24], partial agonists can be particularly beneficial therapeutically if the GR still delivers full gene repression capacity, despite its lower GRE‐driven gene activation. Functional ligand activation of the GR was also confirmed via pSer211 Western blotting [25, 26, 27]. Partial agonism was further supported by differential patterns in pSer203 and pSer226 Western blotting [28, 29], where **AzoCort1** favors Ser211 phosphorylation compared to Dex (see Figure S2).

**FIGURE 3:**
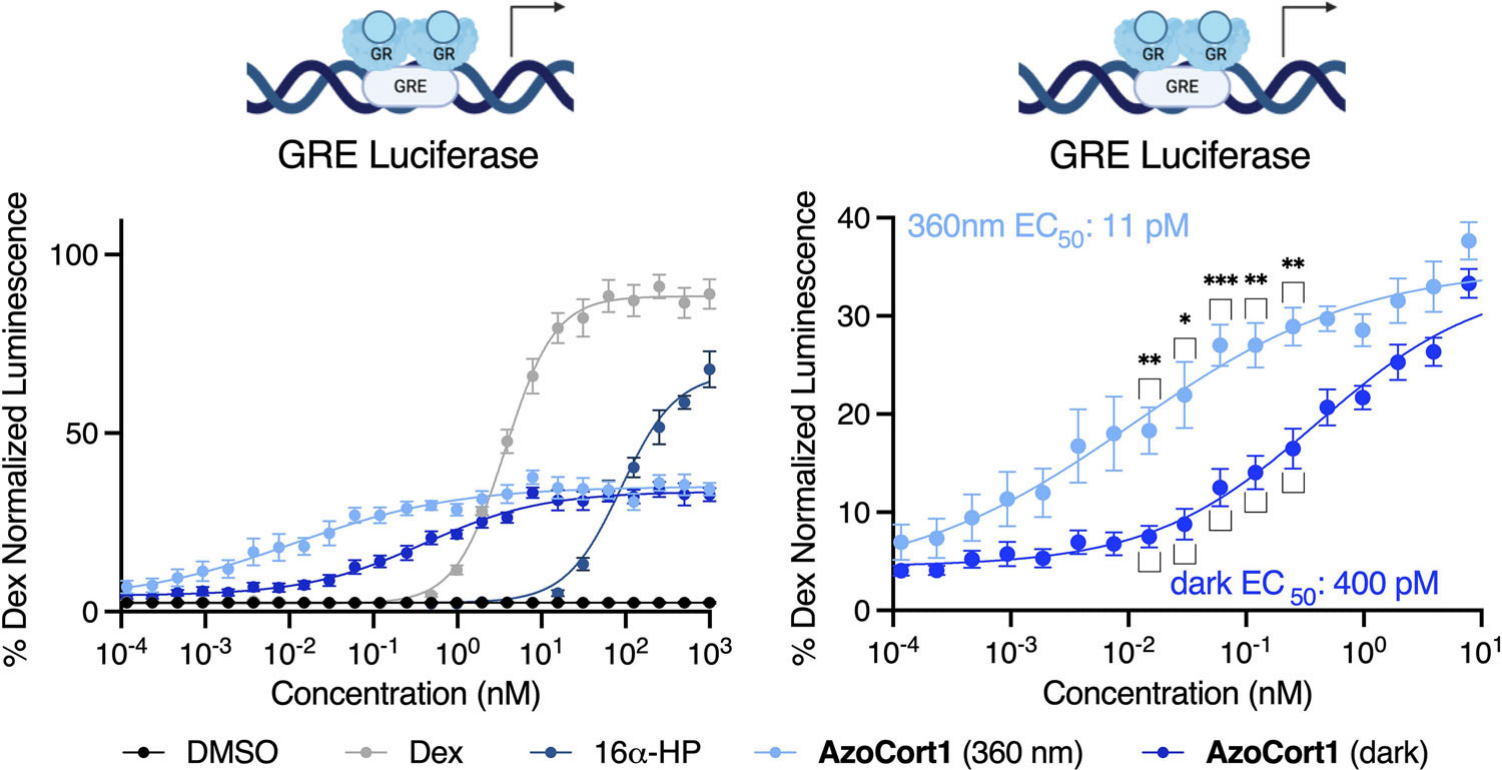
Concentration‐response curves showing **AzoCort1** light‐dependent promotion of GRE‐driven transcription. GRE reporter A549 cells were treated for 6 h with compound, maximally 0.1% DMSO, 1 µM Dex, dark **AzoCort1**, or **AzoCort1** irradiated for 15 min with 360 nm light, then serially diluted. Results are normalized to positive control (Dex). The right panel is zoomed in on **AzoCort1** results. Curves were generated using 24 concentrations per condition. Data are shown as mean ± SEM of dexamethasone‐normalized luciferase counts (%) from *n* = 7 matched biological replicates. Two‐way ANOVA was performed with Šídák's multiple comparisons test. *p* < 0.05 (*), *p* < 0.01 (**), *p* < 0.001 (***), *p* < 0.0001 (****); ns = not significant.

**AzoCort 1** remained light‐responsive in a cellular environment, as irradiation of the cell‐culture plates with 360 nm light [28] produced a modest ∼4‐fold increase in **AzoCort1** potency. Noteworthy, pre‐irradiation could be attenuated by irradiation at 420 nm, which favors the less active *trans* isomer (Figure S1). Pre‐irradiation, however, produced the greatest separation in activity and was therefore used throughout this study.

To assess the anti‐inflammatory potential of **AzoCort1**, we performed NF‐κB and AP‐1‐dependent reporter assays in which a pro‐inflammatory stimulus triggers TF‐mediated gene expression. **AzoCort1** was found to be essentially as efficacious as Dex in suppressing NF‐κB and AP‐1‐dependent gene expression (Figure 4a,b). This contrasts with the GRE‐reporter results, where **AzoCort1** potency was high, but the maximal response was clearly reduced compared to Dex (Figure 3). Against NF‐κB, **AzoCort1** retained high potency, with **AzoCort1** in the dark‐adapted state yielding an IC_50_ value of 923 pM and irradiation at 360 nm delivering a further 72‐fold increase in potency (IC_50_ 13 pM). This trend is maintained across AP‐1 repression, where dark‐adapted **AzoCort1** displayed an IC_50_ of 274 pM, whereas after irradiation at 360 nm the IC_50_ value dropped to 23 pM, a 12‐fold change. These assays highlight that **AzoCort1** retains full gene suppression of NF‐κB and AP‐1 compared to Dex, with enhanced potency. **AzoCort1** also failed to suppress TNF‐induced NF‐κB‐driven gene expression in the counterpart GR KO cell model, demonstrating full GR dependence of the observed gene repression (Figure 4c).

**FIGURE 4:**
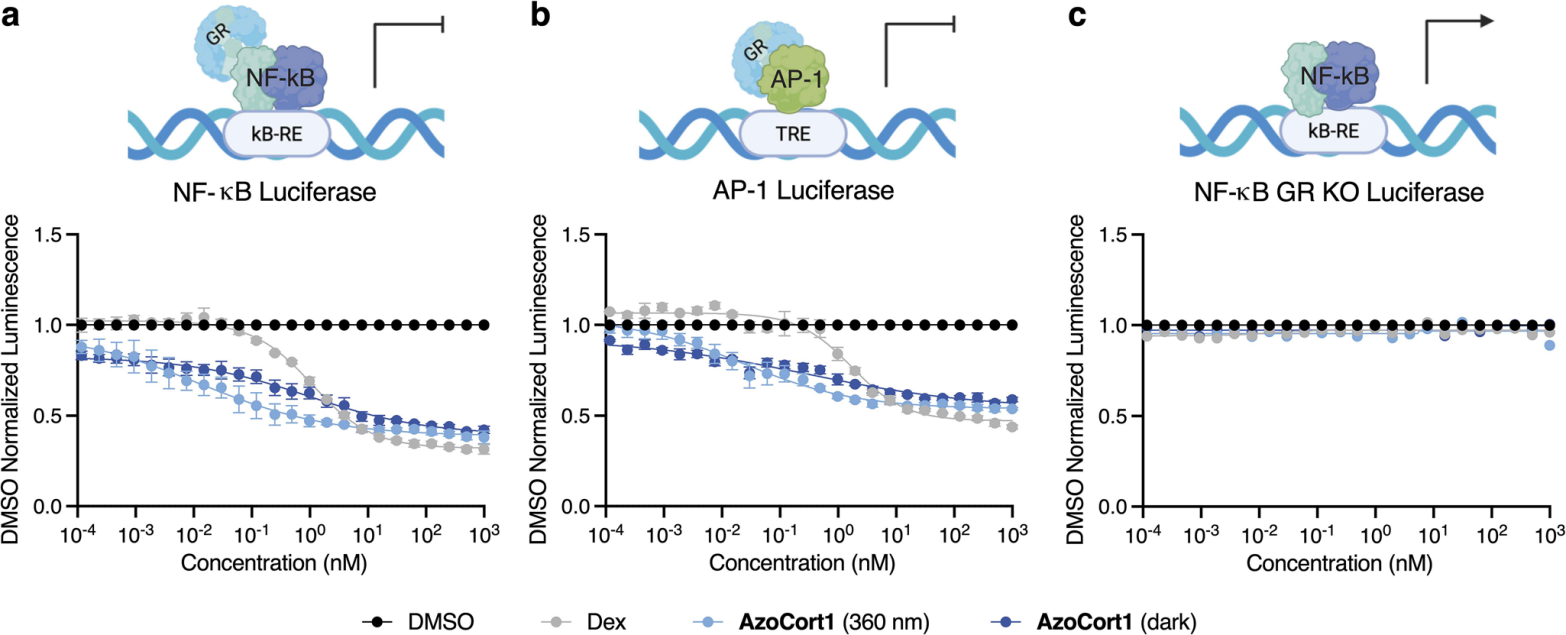
**AzoCort1** induces GR‐dependent, transcriptional gene‐suppressive effects in A549 reporter cells treated for 6 h with compound (maximally 0.1% DMSO, 1 µM Dex, dark **AzoCort1**, or **AzoCort1** irradiated for 15 min with 360 nm light, then serial diluted) and induction of inflammation at 1 h (using 2000 IU/mL TNFα for NF‐κB lines or 100 nM phorbol 12‐myristate 13‐acetate for AP‐1). (a) NF‐𝛋B‐driven luciferase reporter gene counts normalized to vehicle (DMSO). (b) AP‐1‐driven luciferase reporter gene counts normalized to vehicle (DMSO). (c) NF‐𝛋B‐driven luciferase counts normalized to vehicle (DMSO) in GR KO cells. Data are shown as mean ± SEM of DMSO‐normalized luciferase counts from *n* = 3 matched biological replicates.

To further validate the light‐dependent effects of **AzoCort1**, we performed an immunofluorescence assay as an evaluation of GR translocation. Cytosolic GR decreases from 33% (DMSO) to 2% with 1 µM Dex treatment (Figure 5). Irradiated **AzoCort1** induces an approximately 4‐fold decrease in cytosolic GR, with 8% more translocation at 5 nM compared to the dark condition.

**FIGURE 5:**
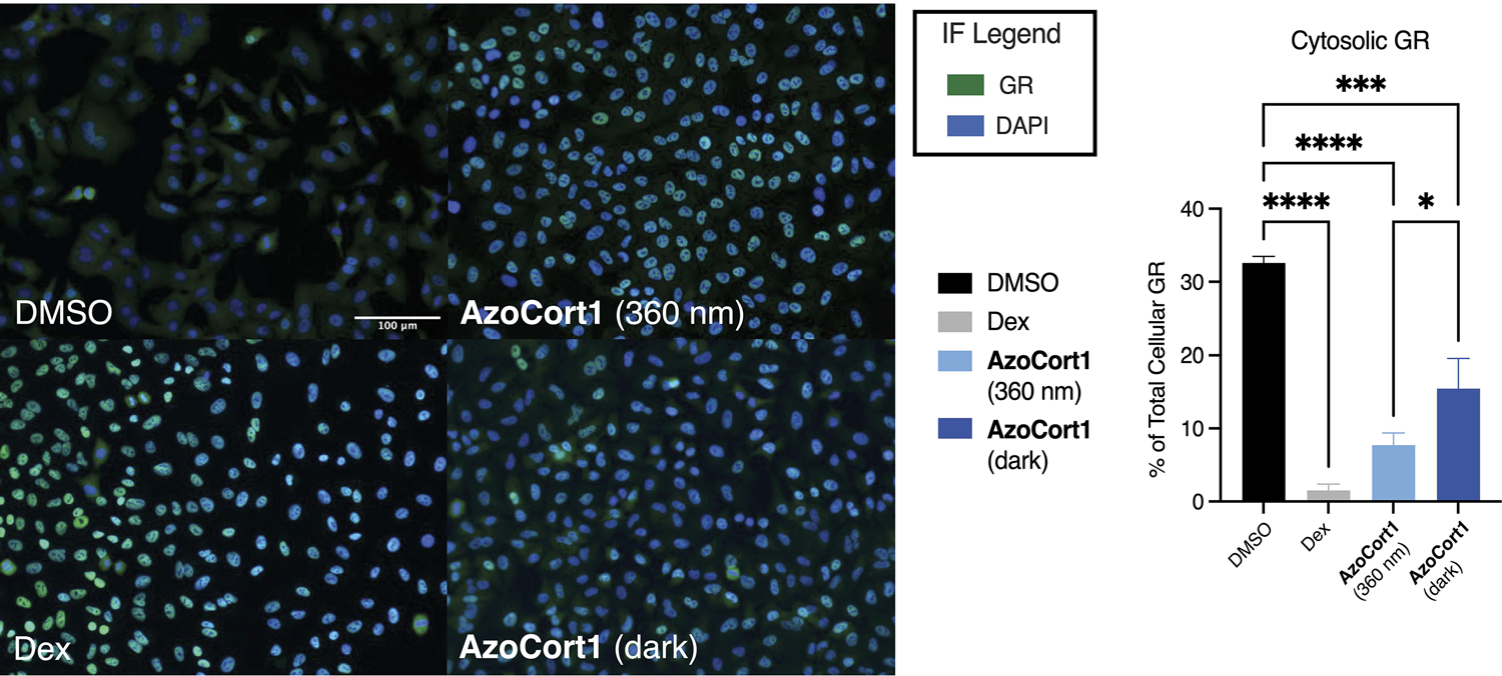
Light‐dependent GR nuclear translocation induced by **AzoCort1**; Representative immunofluorescence images of A549 cells treated for 1 h with (0.5%) DMSO, 1 µM Dex, or 5 nM **AzoCort1** following 15 min irradiation at 360 nm prior to treatment, and 5 nM **AzoCort1** under dark conditions. Images were acquired at 20x magnification, with nuclei stained in blue (Hoechst 33342) and GR in green (1° GR, 2° Alexa Fluor 488 antibodies). Representative images are shown from 10 fields per condition from *n* = 3 biological replicates. Figure images are presented as composites in ImageJ [29]. No adjustments were made, and quantification was done on raw images. Scale bar = 100 µm. GR nuclear translocation was quantified using ImageJ [29]. Data were background‐subtracted using the negative control (Figure S5) and are presented as mean ± SD (*n* = 4 biological replicates). Statistical significance was determined by one‐way ANOVA with Šídák's multiple comparison test. *p* < 0.05 (*), *p* < 0.01 (**), *p* < 0.001 (***), *p* < 0.0001 (****); ns = not significant.

To better understand the molecular basis for the irradiation‐dependent differences in **AzoCort1** activity, we also performed radioligand binding assays using 1,2,4‐[^3^H] dexamethasone. Under optimized conditions (Figure S6), the K_i_ of Dex was determined to be 3.22 nM (Figure 6). In the dark, **AzoCort1** exhibited a K_i_ value of 1.11 nM, whereas upon irradiation with 360 nm light, we observed a 1.6 times lower K_i_ value of 0.69 nM. The transcriptional EC_50_ of 400 pM (dark) and 11 pM (irradiated) is not inconsistent with the K_i_ of 1.11 nM (dark) and 0.69 nM (irradiated), as the K_i_ measures the ligand's biochemical affinity for the receptor ligand‐binding domain, whereas the EC_50_ reflects its functional potency in a cellular transcriptional assay. The latter is influenced by receptor abundance, receptor reserve, dimerization, promoter architecture, coactivator recruitment, intracellular ligand accumulation, and amplification during transcription and reporter expression [30, 31, 32, 33, 34], all of which could be influenced by photoswitching. Consequently, half‐maximal transcription can occur when considerably fewer than half of the receptors are occupied, and the light responsiveness could differ significantly between assays.

**FIGURE 6:**
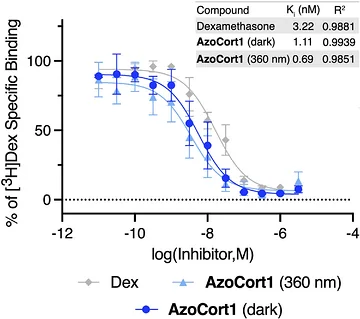
Light‐dependent competitive binding of **AzoCort1** to the GR–LBD. Competition binding curves of increasing concentrations of unlabeled Dex, dark and 360 nm (15 min pre‐irradiation) **AzoCort1** against tritiated dexamethasone (0.82 nM). Data are shown as mean ± SEM from two biological replicates, each performed in technical duplicates. Competition curves were generated by nonlinear regression analysis, and the corresponding K_i_ values and R^2^ values are reported in the inset table.

## 3 Conclusion

In summary, we have introduced **AzoCort1**, a photoswitchable glucocorticoid that functions as a partial agonist and enables light‐dependent activation of the glucocorticoid receptor with picomolar potency. Irradiation with UV–light produced a 36‐fold shift in the EC_50_ for GR‐driven gene activity while maintaining GR‐dependent repression of NF‐κB and AP‐1 signaling. Binding to the GR was confirmed with radiolabel assays and affinity was found to be similar to that of the well‐established glucocorticoid dexamethasone. Our work expands on previous efforts to photosensitize nuclear receptors, including RARα [35], PPARα [36], PPARγ [37], FXR [38], LXR [39], VDR [40, 41], RORγ [42], and ER [43, 44]. As the most potent photopharmaceutical reported to date, **AzoCort1** establishes photoswitchable glucocorticoids as a strategy for the optical control of gene expression [45, 46] and provides a platform for interrogating glucocorticoid receptor signaling with spatial and temporal resolution.

## Author Contributions

**Zoe Sessions**: conceptualization, methodology, formal analysis, data curation, writing – original draft, writing – review and editing, investigation, validation, visualization. **Chloé Van Leene**: investigation, methodology, validation, writing – review and editing. **Dorien Clarisse**: methodology, investigation, writing – review and editing, validation. **George Keen**: investigation, validation, writing – review and editing. **Agnė Šveistytė**: methodology, writing – review and editing. **Dinahlee Saturnino Guarino**: formal analysis, methodology, writing – review and editing. **Robert H. Mach**: supervision, methodology, funding acquisition. **Karolien De Bosscher**: conceptualization, supervision, writing – review and editing, funding acquisition. **Dirk Trauner**: conceptualization, supervision, writing – review and editing, writing – original draft, funding acquisition.

## Conflicts of Interest

The authors declare no conflicts of interest.

## Supporting information



The authors have cited additional references within the Supporting Information [46, 47, 48, 49, 50].**Supporting File**: anie74998‐sup‐0001‐SuppMat.pdf.

## Data Availability

The data that support the findings of this study are available on request from the corresponding author. The data are not publicly available due to privacy or ethical restrictions.

## References

[1] D. Clarisse , L. Van Moortel , C. Van Leene , K. Gevaert , and K. De Bosscher , “Glucocorticoid Receptor Signaling: Intricacies and Therapeutic Opportunities,” Trends in Biochemical Sciences 49 (2024): 431–444, 10.1016/j.tibs.2024.01.012. 38429217

[2] E. R. Weikum , M. T. Knuesel , E. A. Ortlund , and K. R. Yamamoto , “Glucocorticoid Receptor Control of Transcription: Precision and Plasticity via Allostery,” Nature Reviews Molecular Cell Biology 18 (2017): 159–174, 10.1038/nrm.2016.152. PMC6257982 28053348

[3] M. A. Sacta , B. Tharmalingam , M. Coppo , et al., “Gene‐Specific Mechanisms Direct Glucocorticoid‐Receptor‐Driven Repression of Inflammatory Response Genes in Macrophages,” eLife 7 (2018): e34864, 10.7554/eLife.34864. PMC5821458 29424686

[4] L. Escoter‐Torres , F. Greulich , F. Quagliarini , M. Wierer , and N. H. Uhlenhaut , “Anti‐Inflammatory Functions of the Glucocorticoid Receptor Require DNA Binding,” Nucleic Acids Research 48 (2020): 8393–8407, 10.1093/nar/gkaa565. PMC7470971 32619221

[5] L. Escoter‐Torres , G. Caratti , A. Mechtidou , J. Tuckermann , N. H. Uhlenhaut , and S. Vettorazzi , “Fighting the Fire: Mechanisms of Inflammatory Gene Regulation by the Glucocorticoid Receptor,” Frontiers in Immunology 10 (2019): 1859, 10.3389/fimmu.2019.01859. PMC6693390 31440248

[6] M. Oray , K. Abu Samra , N. Ebrahimiadib , H. Meese , and C. S. Foster , “Long‐Term Side Effects of Glucocorticoids,” Expert Opinion on Drug Safety 15 (2016): 457–465, 10.1517/14740338.2016.1140743. 26789102

[7] R. Pofi , G. Caratti , D. W. Ray , and J. W. Tomlinson , “Treating the Side Effects of Exogenous Glucocorticoids; Can We Separate the Good From the Bad?,” Endocrine Reviews 44 (2023): 975–1011, 10.1210/endrev/bnad016. PMC10638606 37253115

[8] K. Hüll , J. Morstein , and D. Trauner , “In Vivo Photopharmacology,” Chemical Reviews 118 (2018): 10710–10747. 10.1021/acs.chemrev.8b00037 29985590

[9] P. Kobauri , F. J. Dekker , W. Szymanski , and B. L. Feringa , “Rational Design in Photopharmacology With Molecular Photoswitches,” Angewandte Chemie International Edition 62 (2023): e202300681, 10.1002/anie.202300681. 37026576

[10] W. A. Velema , W. Szymanski , and B. L. Feringa , “Photopharmacology: Beyond Proof of Principle,” Journal of the American Chemical Society 136 (2014): 2178–2191, 10.1021/ja413063e. 24456115

[11] A. D. Hobson , M. J. McPherson , W. Waegell , et al., “Design and Development of Glucocorticoid Receptor Modulators as Immunology Antibody–Drug Conjugate Payloads,” Journal of Medicinal Chemistry 65 (2022): 4500–4533, 10.1021/acs.jmedchem.1c02099. 35133822

[12] A. D. Hobson , J. Xu , D. S. Welch , et al., “Discovery of ABBV‐154, an Anti‐TNF Glucocorticoid Receptor Modulator Immunology Antibody‐Drug Conjugate (iADC),” Journal of Medicinal Chemistry 66 (2023): 12544–12558, 10.1021/acs.jmedchem.3c01174. 37656698

[13] A. D. Hobson , M. J. McPherson , M. E. Hayes , et al., “Discovery of ABBV‐3373, an Anti‐TNF Glucocorticoid Receptor Modulator Immunology Antibody Drug Conjugate,” Journal of Medicinal Chemistry 65 (2022): 15893–15934, 10.1021/acs.jmedchem.2c01579. 36394224

[14] P. Schake , S. N. Bolz , K. Linnemann , and M. Schroeder , “PLIP 2025: Introducing Protein–Protein Interactions to the Protein–Ligand Interaction Profiler,” Nucleic Acids Research 53 (2025): W463–W465. 10.1093/nar/gkaf361 PMC12230730 40347107

[15] K. Edman , A. Hogner , A. Hussein , et al., GR in Complex with Dexamethasone, Protein Data Bank, 2015, 10.2210/pdb4udc/pdb.

[16] K. Edman , A. Hogner , A. Hussein , et al., GR in Complex with Desisobutyrylciclesonide, Protein Data Bank, 2015, 10.2210/pdb4udd/pdb.

[17] Schrödinger Release **2022–4**: Maestro, Glide, Schrödinger, LLC, New York, NY, 2022.

[18] L. Van Moortel , J. Thommis , B. Maertens , et al., “Novel Assays Monitoring Direct Glucocorticoid Receptor Protein Activity Exhibit High Predictive Power for Ligand Activity on Endogenous Gene Targets,” Biomedicine and Pharmacotherapy 152 (2022): 113218, 10.1016/j.biopha.2022.113218. 35709653

[19] A. D. Hobson , H. Zhu , W. Qiu , R. A. Judge , and K. Longenecker , “Minimising the Payload Solvent Exposed Hydrophobic Surface Area Optimises the Antibody‐Drug Conjugate Properties,” RSC Medicinal Chemistry 15 (2024): 832–838, 10.1039/D3MD00540B. PMC10953475 38516584

[20] L. Ripa , K. Edman , M. Dearman , et al., “Discovery of a Novel Oral Glucocorticoid Receptor Modulator (AZD9567) With Improved Side Effect Profile,” Journal of Medicinal Chemistry 61 (2018): 1785–1799, 10.1021/acs.jmedchem.7b01690. 29424542

[21] X. Liu , Y. Wang , J. S. Gutierrez , et al., “Disruption of a Key Ligand‐H‐Bond Network Drives Dissociative Properties in Vamorolone for Duchenne Muscular Dystrophy Treatment,” Proceedings of the National Academy of Sciences 117 (2020): 24285–24293, 10.1073/pnas.2006890117. PMC7533876 32917814

[22] M. J. Coghlan , P. B. Jacobson , B. Lane , et al., “A Novel Antiinflammatory Maintains Glucocorticoid Efficacy With Reduced Side Effects,” Molecular Endocrinology 17 (2003): 860–869. 10.1210/me.2002-0355 12586843

[23] T. Hegelund Myrbäck , S. Prothon , K. Edman , et al., “Effects of a Selective Glucocorticoid Receptor Modulator (AZD9567) Versus Prednisolone in Healthy Volunteers: Two Phase 1, Single‐Blind, Randomised Controlled Trials,” Lancet Rheumatology 2 (2020): e31–e41, 10.1016/S2665-9913(19)30103-1. 38258274

[24] S. L. Ripp , A. Mukherjee , H. Eng , et al., “In Vitro and In Vivo Investigation of Potential for Complex CYP3A Interaction for PF‐00251802 (Dagrocorat), a Novel Dissociated Agonist of the Glucocorticoid Receptor,” Clinical Pharmacology in Drug Development 7 (2018): 244–255. 10.1002/cpdd.411 PMC5900965 29112329

[25] Z. Wang , J. Frederick , and M. J. Garabedian , “Deciphering the Phosphorylation “Code” of the Glucocorticoid Receptor In Vivo,” Journal of Biological Chemistry 277 (2002): 26573–26580, 10.1074/jbc.M110530200. 12000743

[26] W. Chen , T. Dang , R. D. Blind , et al., “Glucocorticoid Receptor Phosphorylation Differentially Affects Target Gene Expression,” Molecular Endocrinology 22 (2008): 1754–1766, 10.1210/me.2007-0219. PMC2725771 18483179

[27] S. Gadwala and C. Ghosh , “The Role of the Glucocorticoid Receptor and Its Phosphorylation in Neurological Disorders,” International Journal of Molecular Sciences 26 (2025): 4213, 10.3390/ijms26094213. PMC12071575 40362450

[28] M. Borowiak , W. Nahaboo , M. Reynders , et al., “Photoswitchable Inhibitors of Microtubule Dynamics Optically Control Mitosis and Cell Death,” Cell 162 (2015): 403–411. 10.1016/j.cell.2015.06.049 26165941

[29] J. Schindelin , I. Arganda‐Carreras , E. Frise , et al., “Fiji: An Open‐Source Platform for Biological‐Image Analysis,” Nature Methods 9 (2012): 676–682, 10.1038/nmeth.2019. PMC3855844 22743772

[30] A. Kralli and K. R. Yamamoto , “An FK506‐Sensitive Transporter Selectively Decreases Intracellular Levels and Potency of Steroid Hormones,” Journal of Biological Chemistry 271 (1996): 17152–17156, 10.1074/jbc.271.29.17152. 8663352

[31] K. Ronacher , K. Hadley , C. Avenant , et al., “Ligand‐Selective Transactivation and Transrepression via the Glucocorticoid Receptor: Role of Cofactor Interaction,” Molecular and Cellular Endocrinology 299 (2009): 219–231, 10.1016/j.mce.2008.10.008. 19007848

[32] S. H. McLaughlin and S. E. Jackson , “Folding and Stability of the Ligand‐Binding Domain of the Glucocorticoid Receptor,” Protein Science 11 (2002): 1926–1936, 10.1110/ps.5000102. PMC2373678 12142447

[33] Y. Sun , Y. Tao , B. L. Kagan , Y. He , and S. S. jr Simmons ., “Modulation of Transcription Parameters in Glucocorticoid Receptor‐Mediated Repression,” Molecular and Cellular Endocrinology 295 (2008): 59–69, 10.1016/j.mce.2008.05.008. PMC2662735 18583028

[34] S. Robertson , J. M. Rohwer , J. P. Hapgood , and A. Louw , “Impact of Glucocorticoid Receptor Density on Ligand‐Independent Dimerization, Cooperative Ligand‐Binding and Basal Priming of Transactivation: A Cell Culture Model,” PLoS ONE 8 (2013): e64831, 10.1371/journal.pone.0064831. PMC3661511 23717665

[35] J. Morstein , M. Awale , J.‐L. Reymond , and D. Trauner , “Mapping the Azolog Space Enables the Optical Control of New Biological Targets,” ACS Central Science 5 (2019): 607–618, 10.1021/acscentsci.8b00881. PMC6487453 31041380

[36] S. Willems , J. Morstein , K. Hinnah , D. Trauner , and D. Merk , “A Photohormone for Light‐Dependent Control of PPARα in Live Cells,” Journal of Medicinal Chemistry 64 (2021): 10393–10402, 10.1021/acs.jmedchem.1c00810. 34213899

[37] K. Hinnah , S. Willems , J. Morstein , et al., “Photohormones Enable Optical Control of the Peroxisome Proliferator‐Activated Receptor γ(PPARγ),” Journal of Medicinal Chemistry 63 (2020): 10908–10920, 10.1021/acs.jmedchem.0c00654. PMC11684002 32886507

[38] J. Morstein , J. B. Trads , K. Hinnah , et al., “Optical Control of the Nuclear Bile Acid Receptor FXR With a Photohormone,” Chemical Science 11 (2020): 429–434, 10.1039/C9SC02911G. PMC7067245 32190263

[39] T. K. Mukhopadhyay , S. Willems , C. J. Arp , et al., “Development of Light‐Activated LXR Agonists,” ChemMedChem 18 (2023): e202200647, 10.1002/cmdc.202200647. 36896647

[40] X. Rovira , A. Espada , C. Serra , et al., “Light‐Regulated Agonists Spatiotemporally Activating the Vitamin D Receptor Mitigate Psoriasis‐Like Inflammation in Mice Without Inducing Hypercalcemia,” ACS Central Science 11 (2025): 2340–2352. 10.1021/acscentsci.5c00987 PMC12746157 41473792

[41] X. Ge , S. Willems , F. Melfi , et al., “An Agonist/Antagonist Photo‐Switchable Vitamin D Mimetic Enables Bidirectional Optical Control of VDR,” Angewandte Chemie International Edition 65 (2026): e9726457, 10.1002/anie.9726457. PMC13360702 42112826

[42] M. Reynders , S. Willems , J. A. Marschner , T. Wein , D. Merk , and O. Thorn‐Seshold , “A High‐Quality Photoswitchable Probe That Selectively and Potently Regulates the Transcription Factor RORγ,” Angewandte Chemie International Edition 63 (2024): e202410139, 10.1002/anie.202410139. PMC11586699 39248642

[43] J. Ewert , L. Heintze , M. Jordà‐Redondo , et al., “Photoswitchable Diazocine‐Based Estrogen Receptor Agonists: Stabilization of the Active Form Inside the Receptor,” Journal of the American Chemical Society 144 (2022): 15059–15071, 10.1021/jacs.2c03649. 35952371

[44] K. Tsuchiya , T. Umeno , G. Tsuji , et al., “Development of Photoswitchable Estrogen Receptor Ligands,” Chemical and Pharmaceutical Bulletin 68 (2020): 398–402, 10.1248/cpb.c19-01108. 32238658

[45] M. Yamada , S. C. Nagasaki , T. Ozawa , and I. Imayoshi , “Light‐Mediated Control of Gene Expression in Mammalian Cells,” Neuroscience Research 152 (2020): 66–77, 10.1016/j.neures.2019.12.018. 31923448

[46] K. Y. Manoilov , V. V. Verkhusha , and D. M. Shcherbakova , “A Guide to the Optogenetic Regulation of Endogenous Molecules,” Nature Research 18 (2021): 1027–1037, 10.1038/s41592-021-01240-1. 34446923

[47] L. L. C. Schrödinger , *The PyMOL Molecular Graphics System, Version∼2.5.5*, (2022).

[48] GraphPad Prism, Version 10.6, GraphPad Software, Boston, MA, USA, **2025‐2026**.

[49] GelAnalyzer, version 23.1.1, István Lázár, Budapest, Hungary, **2023**.

[50] C. W. Chu , L. Stricker , T. M. Kirse , M. Hayduk , and B. J. Ravoo , “Light‐Responsive Arylazopyrazole Gelators: From Organic to Aqueous Media and From Supramolecular to Dynamic Covalent Chemistry,” Chemistry—A European Journal 25 (2019): 6131–6140. 10.1002/chem.201806042 PMC6593461 30791165

