# Supplementary material for: Optical Control of the Glucocorticoid Receptor Activity With a Photoswitchable Agonist
Source: Angew Chem Int Ed Engl. 2026 Sep 26;65(45):e9699968. doi: 10.1002/anie.9699968 (PMC13725556; doi:10.1002/anie.9699968)
Supplement: Supplementary file 1 [file ANIE-65-e9699968-s001.pdf]

# Optical Control of the Glucocorticoid Receptor Activity with a Photoswitchable Agonist

# Supporting Information

## Table of Contents


**GENERAL EXPERIMENTAL INFORMATION** .......... **2**

**MATERIALS & REAGENTS** .......... **2**

Chemical Reagents .......... 2
Biological Reagents .......... 3

**MOLECULAR BIOLOGY METHODS** .......... **4**

Cell Culture .......... 4
Immunofluorescence & Fluorescence Microscopy .......... 4
Reporter Assays (GRE, Constitutive, NF-κB, NF-κB GR-KO, AP-1) .......... 5
Western Blotting .......... 5
Radioligand Binding Assays .......... 6
Equipment & Technology .......... 6

**SUPPLEMENTARY FIGURES** .......... **6**

Figure S1. In-Cell DISCO Irradiation of AzoCort1 .......... 6
Figure S2. Western Blot Total GR Vs. Phosphorylated Ser203, Ser211, and Ser226 GR Western Blot .......... 7
Figure S3. AzoCort2-6 GRE Screen .......... 8
Figure S4. Toxicity Assay .......... 8
Figure S5. Negative Control Immunofluorescence .......... 9
Figure S6. Radioligand Competition Binding Assay Optimization .......... 9
Figure S7. Unedited Western Membranes & Chemiluminescence .......... 10

**CHEMICAL SYNTHESIS & CHARACTERIZATION** .......... **11**

General Procedures .......... 11
Synthetic Procedures and Compound Characterization .......... 11
*Azoarene **1*** .......... *11*
*Azoarene **2*** .......... *12*
*Azoarene **3*** .......... *13*
*Azoarene **4*** .......... *13*
*Azoarene **5*** .......... *14*
*Azoarene **6*** .......... *15*
*Azoarene **7*** .......... *15*
*Azoarene **8*** .......... *16*
*Compound 9 (**AzoCort1**) & Compound 9β (**AzoCort1β**)* .......... *17*

*Compound 10 (**AzoCort2**) & Compound 10β (**AzoCort2β**)* ........ 18
*Compound 11 (**AzoCort3**) & Compound 11β (**AzoCort3β**)* ........ 20
*Compound 12 (**AzoCort4**) & Compound 12β (**AzoCort4β**)* ........ 23
*Compound 13 (**AzoCort5,** 8:1 mixture C22α: C22β)* ........ 25
*Compound 14 (**AzoCort6**) & Compound 14β (**AzoCort6β**)* ........ 26
INDEX OF SPECTRA ........ 28
LC-MS PURITY TRACES ........ 72
UV-VIS CHARACTERIZATION ........ 78
*Methods* ........ 78
*Wavelength Scan, Thermal Relaxation, & Photocycling Spectra* ........ 79
**COMPUTATIONAL METHODS ........ 85**
MOLECULAR DOCKING ........ 85
DATA PROCESSING ........ 85
*Immunofluorescence Data Processing* ........ 85
**SUPPLEMENTARY TABLES ........ 90**
HPLC PURIFICATION METHODS ........ 90
ANTIBODY SPECIFICATIONS ........ 90
AZOCORT GRE LUCIFERASE $EC_{50}$ VALUES ........ 91
**REFERENCES ........ 92**

# General Experimental Information

## Materials & Reagents

### Chemical Reagents

1,2,4-$^3$H Dexamethasone was purchased from American Radiolabeled Chemicals (ART1107). Dexamethasone (CAS No 50-02-2, 98% purity) was purchased from NetaScientific (AstaTech-30767). 16α-hydroxyprednisolone (CAS No 13951-70-7, 97% purity) was purchased from aablocks (AA001B3K). Triamcinolone (CAS No 124-94-7, 97% purity) was purchased from Combi-Blocks (MFCD00010477). Fluocinolone acetonide (CAS No 67-73-2, 98% purity) was purchased from Cayman Chemical (23027-500). AzoCorts were made according to the synthesis below. All compounds were prepared as 10 mM stocks in DMSO and stored in 10μL aliquots at -20°C to avoid freeze-thaw cycles. Final

compound purity was assessed by LC-MS analysis). Light-sensitive compounds were handled under red light or reduced ambient light conditions. All other reagents and solvents were obtained from commercial suppliers (Ambeed, Thermo Fisher Scientific, Oakwood Chemical, Cayman Chemical, and Neta Scientific) and used without further purification unless otherwise specified. Where indicated, solvents were rendered anhydrous by passage through a solvent purification system and stored under an inert atmosphere. Flash column chromatography was performed on a CombiFlash NextGen 3000 system (Teledyne ISCO) using silica gel (60 Å, 40–63 µm). NMR spectra were recorded on 400, 500, and 600 MHz spectrometers. Chemical shifts (δ) are reported in ppm relative to residual solvent signals. HRMS analysis was performed on an Agilent LC–MS system with electrospray ionization (ESI).

## Biological Reagents

Cells were cultured using DMEM - high glucose w/o phenol red (1x) (Invitrogen 21063029) with 10% Fetal Bovine Serum (Corning MT35-010-CV) and 1mM sodium pyruvate (Invitrogen 11360070), PBS pH 7.4 (Invitrogen 10010023), and 0.25% Trypsin-EDTA (Invitrogen 25200056). Recombinant human TNFα was purchased from ThermoFisher (PeproTech 300-01A-50UG) as a lyophilized solid that was dissolved in MilliQ dd$H_2O$ to 7.82E6 IU/mL and stored in 10µL aliquots at -80°C. Phorbol 12-myristate 13-acetate (PMA, CAS No 16561-29-8, 95% purity) was purchased from Ambeed (A175370-5mg) then dissolved to 10mM stock in DMSO and stored in 10µL aliquots at -20°C. Hoescht 33342 (CAS No 23491-52-3, 99% purity) was purchased from Ambeed (A186328-50mg) then dissolved to 10mM stock in DMSO and stored in 10µL aliquots at -20°C. Antibodies were purchased from Cell Signaling or Sigma Aldrich(see Table S2 Antibody Specifications & Usage), except AlexaFluor 488 Goat anti-Rabbit IgG, which was purchased from ThermoFisher (Thermo A-11008). 4% paraformaldehyde in PBS (CAS No 30525-89-4) was purchased from ThermoScientific (J19943-K2). Bovine Serum Albumin (BSA, CAS 9048-46-8) was purchased from SigmaAldrich (A4737-100G). Triton X-100 was purchased from Sigma (T8787-100mL). Tricine (CAS No 5704-04-01) was purchased from Fisher Scientific (AAA1469522). Magnesium Sulfate Heptahydrate (CAS No 10034-99-8) was purchased from Fisher Scientific (1337184). D-Luciferin Potassium was purchased from Ambeed (A162108-5g). Coenzyme A (CAS No 85-61-0) was purchased from Ambeed (A148168-1g). Magnesium carbonate hydroxide pentahydrate (CAS No 56378-72-4) was purchased from Ambeed (A1001065-25g). 1,4-Dithiothreitol (DTT, CAS No 3483-12-3) was purchased from Ambeed (A587635-25g). Ethylenediaminetetraacetic acid disodium dihydrate (EDTA, CAS No 6381-92-6) was purchased from Ambeed (A626916-100g). Sodium Molybdate (NaMoO4, CAS 7631-95-0) was purchased from VWR (131760-100g). Adenosine 5'-triphosphate disodium salt hydrate (ATP, CAS No 34369-07-08) was purchased from

Ambeed (A471068-5g). Sodium chloride (CAS 7647-14-5) was purchased from Oakwood Chemicals (OAK-094554-20KG). Potassium chloride (CAS 7447-40-7) was purchased from SigmaAldrich (P3911-500G). Glycerol was purchased from Invitrogen (15514011). Tris-HCl pH 7.5 was purchased from Invitrogen (15567027). Protease Inhibitor Cocktail-Mammalian w/o Metal Chelators was purchased from Sigma (P8340-1ML). Halt Phosphotase Inhibitor cocktail was purchased from Pierce Endogen (78420). Super Signal West Pico Plus and SuperSignal West Femto Chemiluminescent Substrate were purchased from Pierce Endogen (PI34580, PI34095). BCA Protein Assay Kit was purchased from Pierce Endogen (23225). PageRuler Protein Ladder was purchased from ThermoFisher (26616). Bio-Rad Precision Plus Protein Dual Color Standards was purchased from Bio-Rad (1610374).

# Molecular Biology Methods

## Cell Culture

A549 (ATCC CCL-185) and A549 cells stably expressing $(NF\text{-}\kappa B)_3$_Luc, GRE_Luc, $collagenase_3$_Luc or luciferase driven by a constitutive promoter (Const_Luc) were previously described.[1] The A549 $(NF\text{-}\kappa B)_3$_Luc GR knock-out (KO) cell line was generated from the A549 $(NF\text{-}\kappa B)_3$_Luc cell line via the CRISPR/Cas9 system targeting exon 3 (CATTATGGAGTCTTAACCTTG) and was previously described.[1] All cells were kindly provided by Prof. Karolien De Bosscher and maintained at 37°C with 5% $CO_2$ for culture, using DMEM without phenol red supplemented with 10% FBS and 1% sodium pyruvate. Cells were passaged at ~80% confluency using .25% trypsin-EDTA and not kept above passage 20. Cells were tested for mycoplasma contamination and confirmed negative. For all experiments, 24h after seeding and 16h before compound treatment, all cells were serum-deprived.

## Immunofluorescence & Fluorescence Microscopy

30,000 A549 cells were seeded in iBidi 8-well µ-slides (iBIDI 80826) and following 1 hour treatment with compound (0.5% DMSO, 1µM Dex, or 5nM 360nm irradiated or dark AzoCort1), cells are fixed with 4% PFA in PBS. Primary staining was done with GR antibody (Anti-Rb, Cell Signaling 12041S), except in the negative control which receives only blocking buffer (blocking buffer is 0.2% Triton X-100 and 5% FBS in PBS). Secondary staining is done with AlexaFluor 488 Goat anti-Rabbit IgG (Thermo A-11008) and 100nM Hoecsht 33342 (CAS No. 23491-52-3) to visualize nuclei. Each well is imaged in 10 distinct locations, and the assay was repeated 3 times. Figure images are presented as composites in ImageJ[2]. No other adjustments were made and quantification was done on raw TIF files. The scale for the images is 0.255µm/pixel (3.9216pixel/µm). ImageJ[2] was used to build

nuclear and cytosolic classes then GraphPad Prism (version 10.5)[3] was used to calculate total % using quantification of grey value and for statistical analysis. Statistical significance was determined by one-way ANOVA with Šídák's multiple comparison test.

## Reporter Assays (GRE, Constitutive, NF-kB, NF-kB GR-KO, AP-1)

A549 cells with stably integrated $(NF\text{-}\kappa B)_3$_Luc, GRE_Luc, $collagenase_3$_Luc or luciferase driven by a constitutive promoter (Const_Luc) were seeded in white 96-well plates at 10,000 cells/well. Each compound (0.1% DMSO,1μM Dex or AzoCorts) was tested in triplicate in a 16- or 24-point serial dilution dose response curve from 1μM to 30pM or 119fM for 6 hours. NF-κB driven reporter lines are stimulated with 2000 IU/mL TNF$\alpha$ at 1 hour and AP-1 lines are stimulated with 100nM phorbol 12-myristate 13-acetate (PMA, CAS No. 16561-29-8) at 1 hour. At 6 hours, the media is removed and cells are lysed with 50μL 1x Cell Culture Lysis Reagent (CCLR, 25mM Tris-HCl pH 7.5, 10mM DTT, 10mM CDTA, 50% glycerol, 5% Triton X-100) and shaken for 10 minutes. 35μL luciferin substrate (Luci, 40mM Tricine, 2.14mM $(MgCO_3)_4Mg(OH)_2 \cdot 5H2O$, 5.24mM $MgSO_4 \cdot 7H2O$, 66.6mM DTT, 0.2mM EDTA, 270μM Coenzyme A, 530μM ATP, 470μM Luciferin) is added to each well and the plate is read immediately using a FLUOStar Omega plate reader for luciferase. Data were normalized against the respective control and each assay was conducted at least 4 times to allow robust curve fitting (nonlinear fit, 4 parameters GraphPad Prism version 10.5).

## Western Blotting

500,000 A549 cells were seeded per well in 6-well plates. The cells were treated with 0.1% DMSO, 1μM Dex, or 4nM 360nm irradiated or dark AzoCort1 for 2 hours. The cells were then washed with PBS and lysed in TOTEX cell buffer (50mM Tris-HCl, 150mM NaCl, 1mM EDTA, 1% v/v Triton X-100, 1% HALT inhibitor cocktail and 1% phosphatase inhibitor cocktail). Cell pellets were clarified and protein levels quantified using BCA assay to prepare western samples. NUPage Bis-Tris gels were run and transferred to nitrocellulose membranes. The membranes were blotted for the GR (Cell Signaling 12041S), phosphorylated Ser211 GR (Cell Signaling 4161S),phosphorylated Ser226 GR (Cell Signaling 97285T), and housekeeping GAPDH (Cell Signaling 97166S) including PageRuler Prestained Protein Ladder or Bio-Rad Precision Plus Protein Dual Color Standards markers. See Table S2 Antibody Specifications & Usage for more information. Following treatment with chemiluminescent substrate, the membranes were imaged using Invitrogen Chemiluminescence iBright1500 (Invitrogen) and quantified using GelAnalyzer (version 23.1.1)[4].

## Radioligand Binding Assays

Optimal competition conditions were determined to be 1000nM GR-LBD (ThermoFisher A15668) in 10mM modified KPBS (pH 7.4 + 50mM KCl, 10mM NaMoO4, 0.1mM EDTA, 1mM DTT, and 0.1% BSA) with 90-minute incubation at 22°C. Assays were run in 96-well plates with PEI-coated filter plates. Protein concentration was determined using 5 nM [$^3$H] Dexamethasone (American Radiolabeled Chemicals ART1107, 40Ci/mmol) and a concentration curve of GR-LBD ranging from 67.5-3000nM at 4°C, 22°C, and 37°C. Lack of ligand depletion was confirmed by Zone A analysis (<10% ligand binding). Saturation binding curves (n=2 replicates in triplicate) were run at 22°C for 90 minutes using 1000nM GR-LBD and 0.024-50nM [$^3$H] Dexamethasone. $B_{max}$ and $K_D$ were determined to be 530.2 and 0.21 nM, respectively, using one site specific binding nonlinear regression in GraphPad Prism (version 11.0). Competition binding assays (n=2 replicates in duplicate) were performed using 1000nM GR-LBD, 0.82nM [$^3$H] Dexamethasone at 22°C for 90 minutes with competing ligand concentrations ranging from .01-3000nM, all in the same modified KPBS buffer. $K_i$ for competition ligands were determined via one site fit $K_i$ nonlinear regression in GraphPad Prism (version 11.0).

## Equipment & Technology

Irradiation was done with 360nm or 420nm LEDs on a cell DISCO plate (XSL-360-5E or VL420-5-15 Roithner LaserTechnik) for 15 mins prior to treatment. In-cell irradiation was performed using DISCO plates with 75ms of light every 15s. Immunofluorescence imaging was completed using a 20x objective on an EVOS M7000 microscope using DAPI and GFP filter cubes. 96-well plates were read using FLUOStar Omega plate reader. Western blots were imaged using iBright1500 (Invitrogen) and quantified using GelAnalyzer (version 23.1.1)[4]. All graphs were made in GraphPad Prism (version 10.5 or version 11.0)[3].

# Supplementary Figures

## Figure S1. In-Cell DISCO Irradiation of AzoCort1

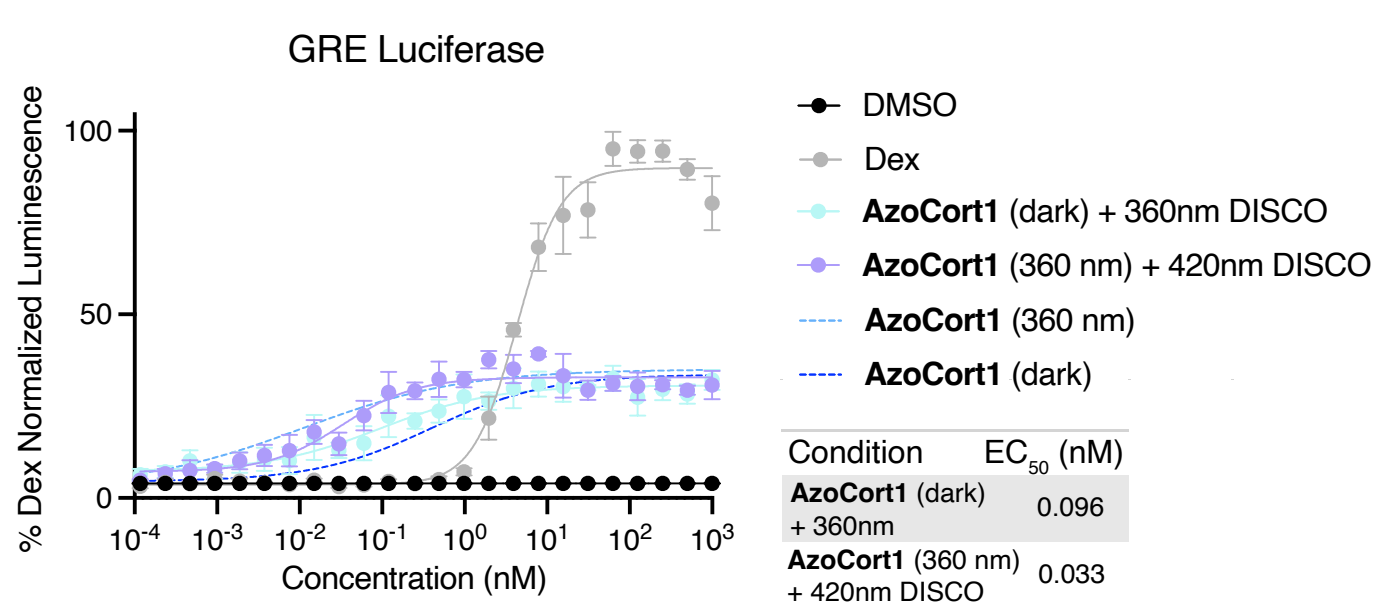

*Figure S1. Concentration-response curves showing AzoCort1 light-responsive promotion of GRE-driven transcription. GRE reporter A549 cells were treated for 6 hours with compound, maximally 0.1% DMSO, 1 μM Dex, **AzoCort1** (dark), or **AzoCort1** (360nm), which is irradiated for 15 mins with 360nm light, then serially diluted. Cells were incubated on 360nm or 420nm LED DISCOs when indicated. Results are normalized to positive control (Dex). Curves were generated using 24 concentrations per condition. Data are shown as mean ± SEM of dexamethasone-normalized luciferase counts (%) from n = 3 matched biological replicates. **AzoCort1** (360 nm) and **AzoCort1** (dark) are overlaid from **Figure 3**.*

# Figure S2. Western Blot Total GR Vs. Phosphorylated Ser203, Ser211, and Ser226 GR Western Blot

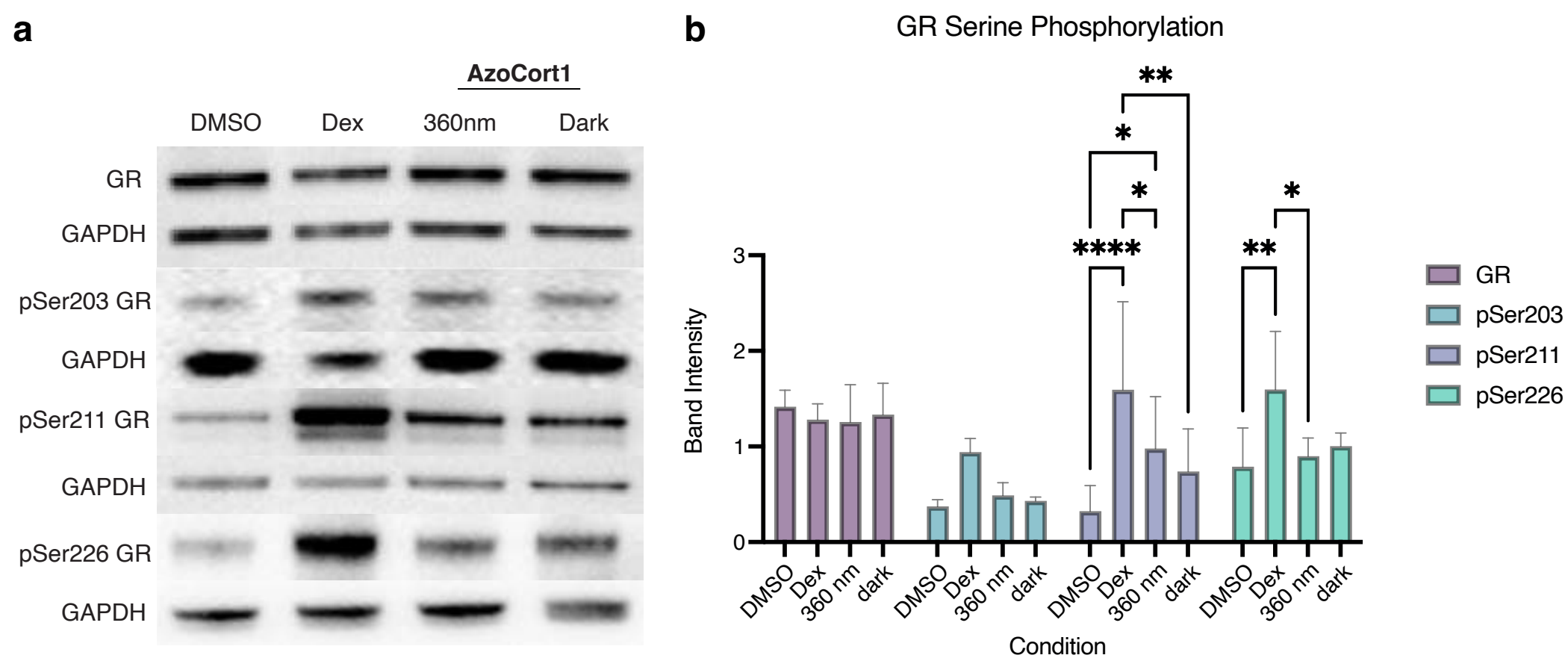


*Figure S2. Irradiation leads to an observed increase in Ser 211 phosphorylation and a decrease in Ser226 phosphorylation. **a**) Western blot of whole A549 cell lysates blotted for total GR, pSer203, pSer211, and pSer226 GR normalized against GAPDH as control following treatment with 0.01% DMSO, 1 μM Dex, 4 nM **AzoCort1** (360 nm) after 15mins 360nm irradiation prior to treatment, or 4 nM **AzoCort1** (dark). Full blots and raw images available in* **Figure S7**; ***b**) Quantification of western blot data via Gel Analyzer 23.1.1 and plotted with GraphPad Prism 11.0. Data is represented as data ±SD normalized to GAPDH. Two-way ANOVA was run with Šídák's multiple comparisons test. P < 0.05 (*), P < 0.01 (**), P < 0.001 (***), P < 0.0001 (****); ns = not significant.*

# Figure S3. AzoCort2-6 GRE Screen

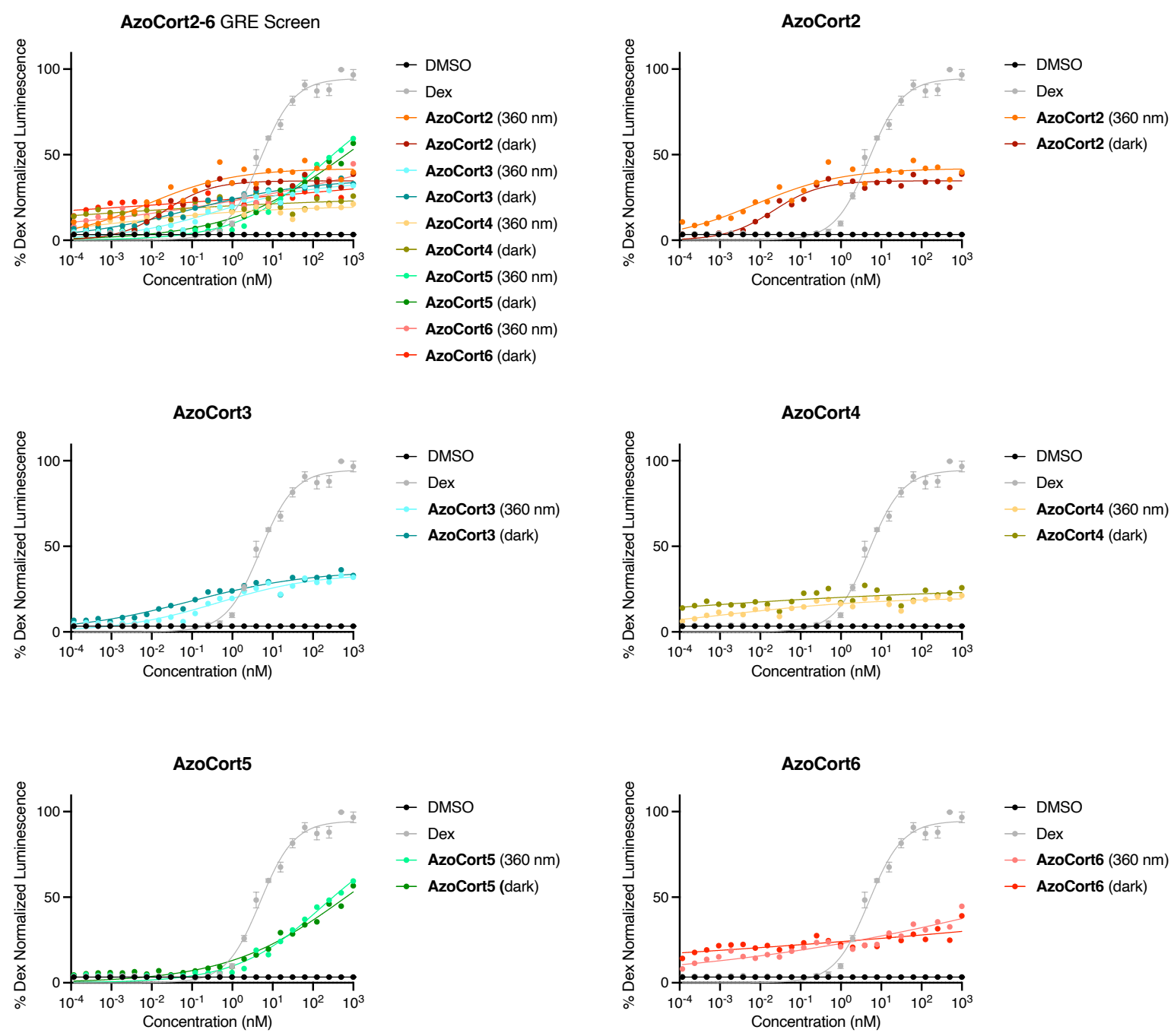


*Figure S3. Dose-response curves showing **AzoCort2-6** light-dependent promotion of GRE transcription. 24 points for each condition are shown as data ± SEM of % dexamethasone normalized luciferase counts from n =1 biological replicate. Data represents luminescent output from treated GRE luc A549 cells. $EC_{50}$ values available in **Table S3** AzoCort GRE Luciferase $EC_{50}$ Values.*

# Figure S4. Toxicity Assay

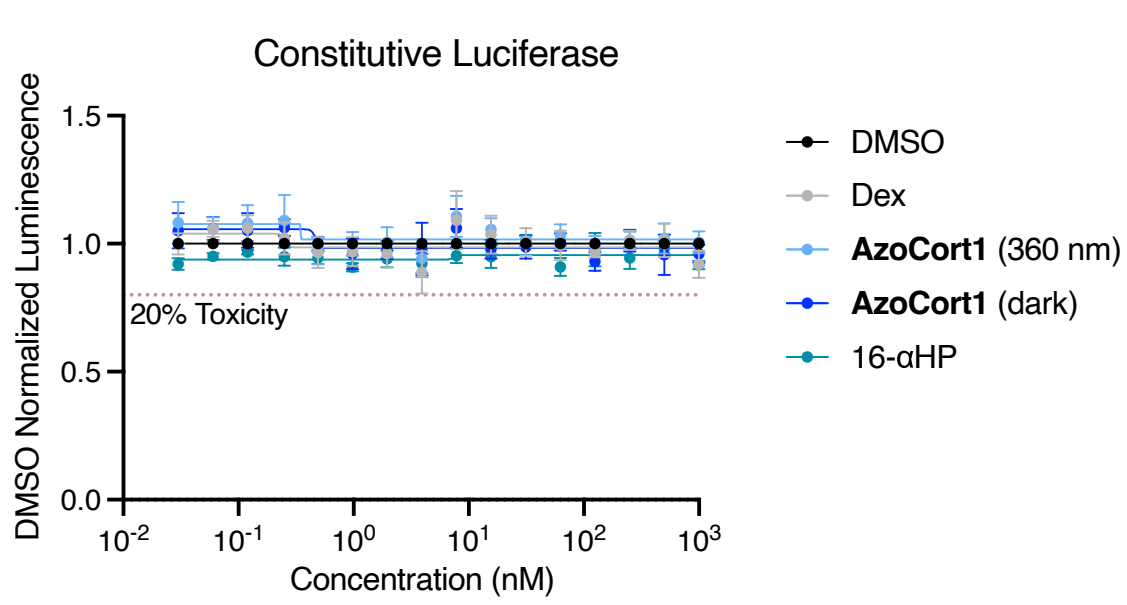


*Figure S4. Dose-response curve of DMSO-normalized luminescent output from constitutive luciferase reporter A549 cells treated with DMSO, Dex, **AzoCort1** (360nm) or **AzoCort1** (dark), or 16-$\alpha$HP as a measure of cytotoxicity. Data is shown as data ± SEM from n =4 biological replicates.*

## Figure S5. Negative Control Immunofluorescence

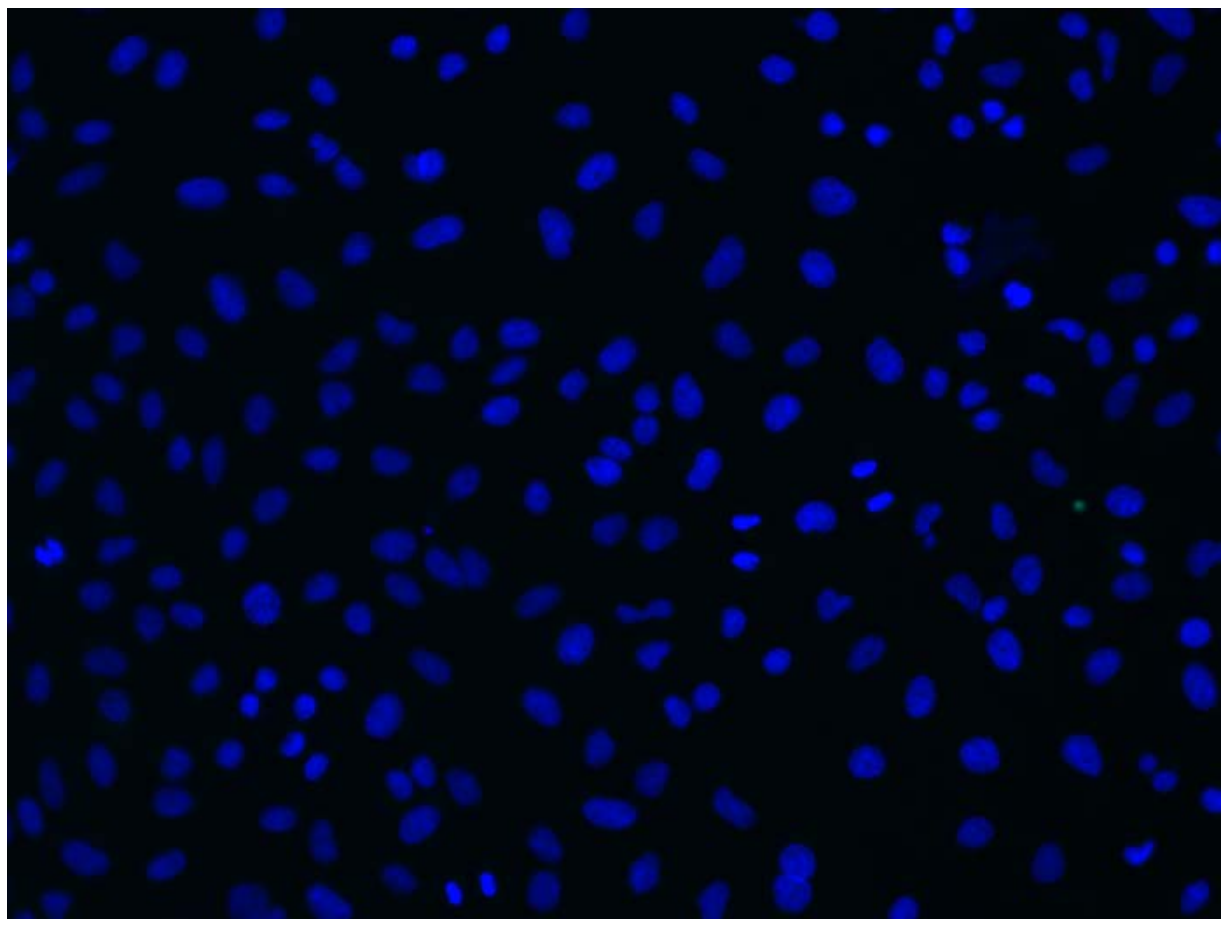

*Figure S5. Negative control immunofluorescence imaging. Representative image of 10 technical replicates from n=3 matched biological replicates. A549 cells were treated with 0.5% DMSO and processed identically to other samples, except not exposed to GR antibody. Images were taken at 20x with nuclei in blue (Hoescht 33342) and GR in green (AlexaFluor-488).*

## Figure S6. Radioligand Competition Binding Assay Optimization

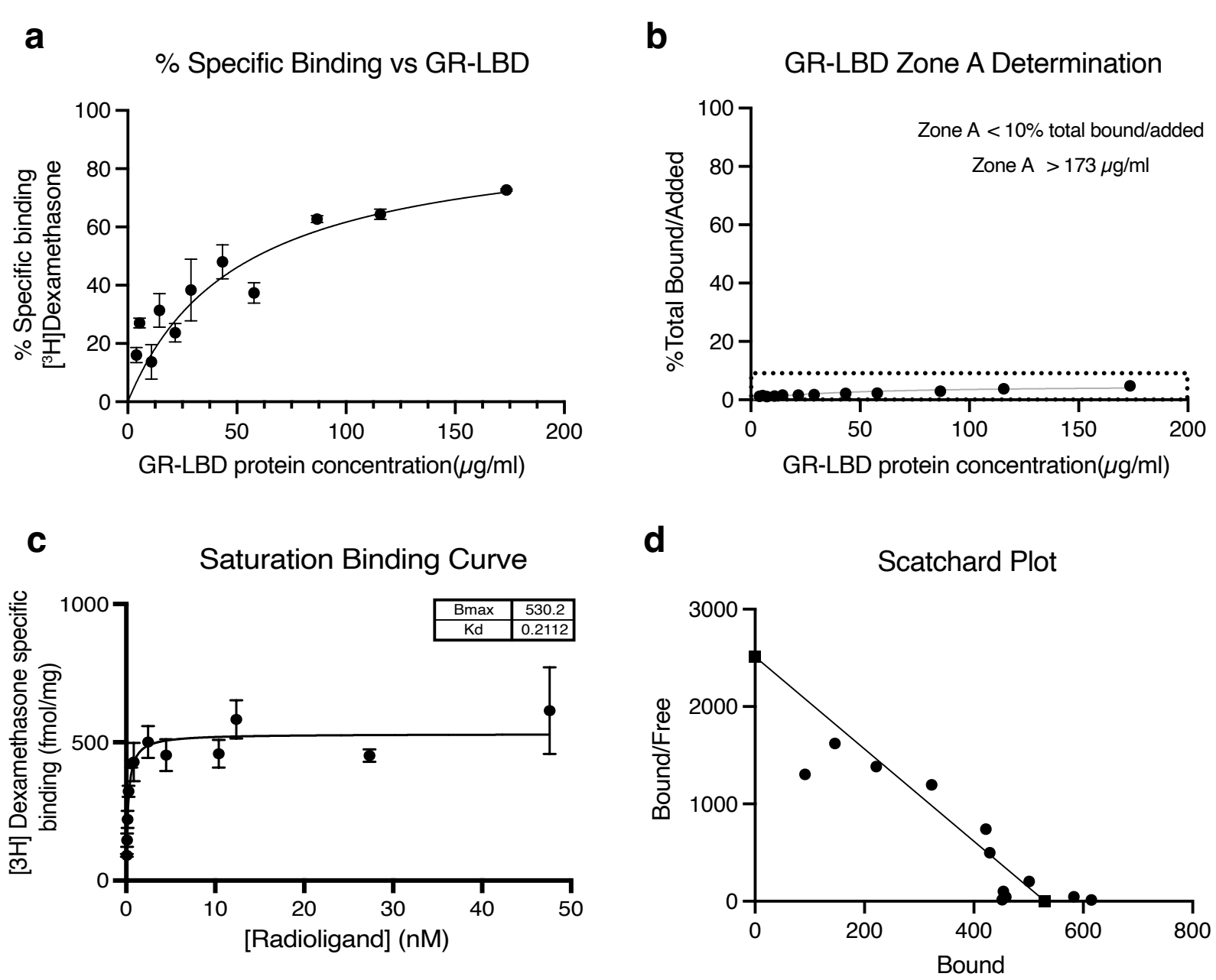


*Figure S6. Radioligand competition binding assay protein and radioligand concentration optimization curves; **a**) Graphs of specific binding of [$^3$H] Dex against varying concentrations of GR-LBD at room temperature; **b**) Zone A determination where GR-LBD did not exceed ligand depletion limits at any concentration tested; **c**) Saturation binding curve of 1000nM GR-LBD binding to varying concentrations of [$^3$H] Dex at room temperature after 90 mins; **d**) Scatchard plot depicting general fit of saturation binding curve. Data is represented as mean (Zone A and Scatchard), mean ±SD (saturation binding curve), or mean ±SEM (% specific binding).*

# Figure S7. Unedited Western Membranes & Chemiluminescence

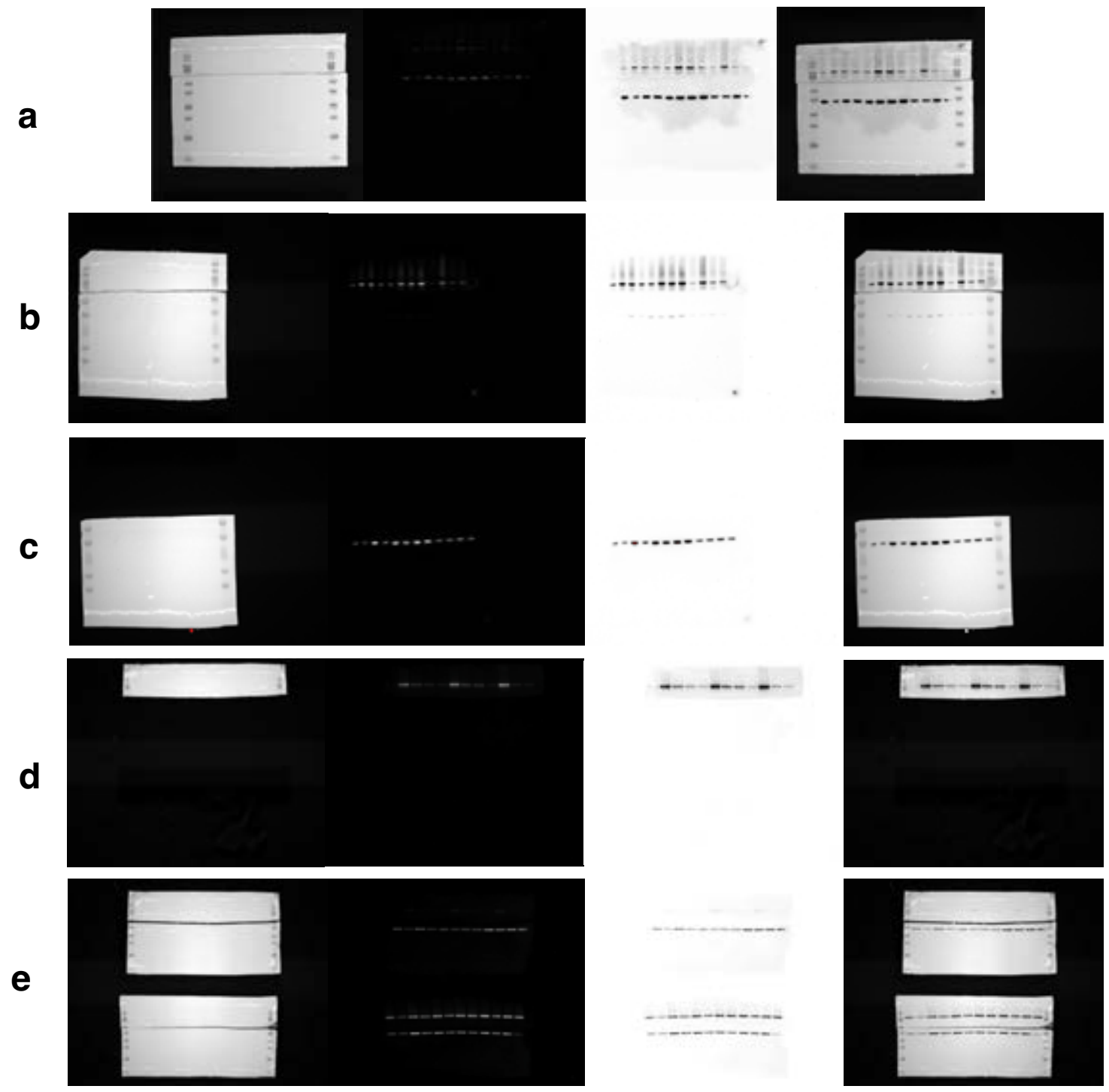


*Figure S7. Raw TIF files of (left to right) western blot membranes, chemi layer (white on black), chemi layer (black on white), and overlay of **a**) pSer203 GR and matching GAPDH; **b**) pSer226 GR and **c**) matching GAPDH; **d**) pSer211 GR and **e**) matching GAPDH blotting (top), with GR and matching GAPDH (bottom). PageRuler Protein Ladder or Bio-Rad Precision Plus Protein Dual Color Standards markers are visible on outside lanes. Blots are of biological triplicate conditions. pSer203 images have been cropped to include only blots presented in this work.*

# Chemical Synthesis & Characterization

## General Procedures

**General Procedure A**

To a 20mL vial was added sterol (1.0 equiv.), aldehyde azobenzene (1.1 equiv.), and MeCN. The solution was stirred then perchloric acid was added (4.0 equiv.). The solution is stirred for 2.5hrs, then quenched with aqueous $NaHCO_3$. The organic is extracted with DCM x3 and dried over $Na_2SO_4$. This material is dried, resuspended in 1.0 mL MeCN, filtered through a 0.22 micron filter, and injected on the HPLC.

**Compound Nomenclature**

Products are designated as AzoCort# (C22α) and AzoCort#β (C22β).

## Synthetic Procedures and Compound Characterization

### Azoarene **1**

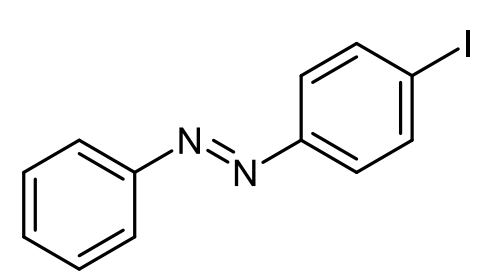

To a 500mL flask was added 200mL of acetic acid and a stir bar. Nitrosobenzene (4.69g, 1.20 equiv., 43.8mmol) and 4-iodoaniline (8.00g, 1.00 equiv., 36.5mmol) are added. The sides of the flask were washed with an additional 200mL of acetic acid (totaling 400mL, 91mM). The solution was stirred for 72 hours at room temperature and checked by TLC. The crude solution (red with orange precipitate) was dried under reduced pressure then redissolved in DCM (100mL). The organic was washed with $NaHCO_3$ (3x, 100mL). The organic was filtered over $Na_2SO_4$ and concentrated under reduced pressure. The crude product was purified by flash column chromatography (eluent: 0-2% ethyl acetate/hexanes). The fractions were combined and dried under reduced pressure to yield (*E/Z*)-1-(4-iodophenyl)-2-phenyldiazene as an orange solid (10.99g, 98%). The m/z 309.085 [M+H]+ was detected via LC-MS at $t_R$ 1.581 & 2.606 on a 3 min gradient of 50-95% MeCN in $H_2O$ (.1% formic acid).

**$R_f$** (19:1 hexanes/ethyl acetate): 0.70

**$^1$H NMR** (400 MHz, $CDCl_3$) δ 7.94 – 7.84 (m, 4H), 7.68 – 7.63 (m, 2H), 7.55 – 7.46 (m, 3H).

**$^{13}$C NMR** (101 MHz, $CDCl_3$) δ 152.6, 152.1, 138.5, 131.5, 129.3, 124.6, 123.1, 97.8.

**IR**: 1615, 1519, 1329, 1072, 834, 705, 594 $cm^{-1}$

**HRMS:** m/z $[M+H]^+$ calculated for $C_{12}H_{10}IN_2$ 308.988322. Measured: 308.9883 Δ-0.1 ppm

## Azoarene **2**

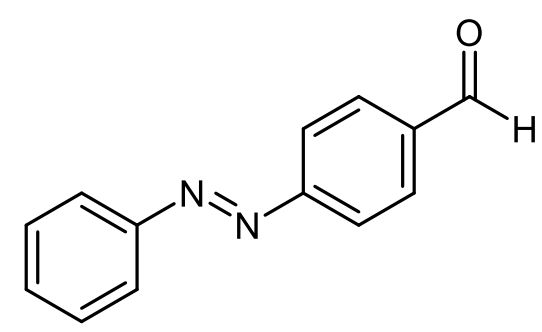


A 250mL flask and stir bar were flame dried and kept under inert gas. 1-(4-iodophenyl)-2-phenyldiazene was added (7.70g, 1.00 equiv., 25.0 mmol) to 160mL of anhydrous THF (837mM) The flask was cooled to -78°C in a bath of acetone and dry ice. After 10 minutes of cooling, *n*-BuLi (1.68g, 10.5mL, 1.05 equiv., 26.3mmol) was added dropwise down the side of the flask to maximize cooling time. The light orange solution immediately turns deep purple. The solution is stirred for 15 mins after which DMF (20.0mL, 10 equiv., 250mmol) is added with a syringe over 10 mins. The solution is stirred for 30 mins at -78°C. The cold solution is quenched with saturated aqueous solution of $NH_4Cl$ (80.0mL) then warmed to room temperature where it turns bright orange. The solution is separated with ethyl acetate (100mL) and washed with aqueous 1M LiCl (x3 200mL) then dried over $Na_2SO_4$. The combined organic is dried then purified by flash chromotagraphy on silica gel (CombiFlash, eluent: 2-6% ethyl acetate/hexanes). The fractions were collected and dried under reduced pressure to yield (*E/Z*)-4-(phenyldiazenyl)benzaldehyde (2.81g, 54%) as an orange solid. (Note: the compound crystallizes out of hexanes as red crystals). The m/z 211.067 [M+H]+ was detected via LC-MS at $t_R$ 0.781 & 1.667 on a 3 min gradient of 50-95% MeCN in $H_2O$ (.1% formic acid).

**$R_f$** (19:1 hexanes/ethyl acetate): 0.30

**$^1$H NMR** (600 MHz, $CDCl_3$) δ 10.11 (d, *J* = 1.2 Hz, 1H), 8.05 (d, *J* = 1.1 Hz, 4H), 8.00 – 7.91 (m, 2H), 7.64 – 7.44 (m, 3H).

**$^{13}$C NMR** (151 MHz, $CDCl_3$) δ 191.6, 155.9, 152.6, 137.5, 132.0, 130.7, 129.3, 123.4, 123.3.

**IR**: 3417, 2981, 2923, 1705, 1599, 1456, 1377, 1197, 1141, 1090, 945, 845, 795, 769, 717, 683, 547, 525 $cm^{-1}$

**HRMS:** m/z $[M+H]^+$ calculated for $C_{13}H_{11}N_2O$: 211.086631. Measured: 211.0866 Δ-0.2 ppm

## Azoarene **3**

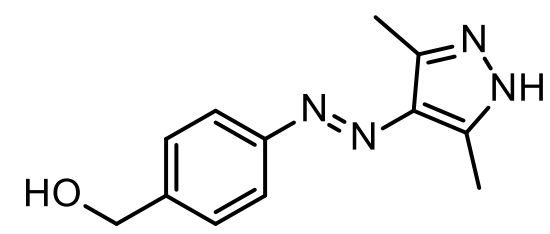

The compound was made according to literature precedent.[5]

**$R_f$** (3:2 ethyl actetate/hexanes): 0.80

**$^1$H NMR** (400 MHz, MeOD-$d_4$) δ 10.04 (s, 1H), 8.03 (d, J = 8.3 Hz, 1H), 7.92 (d, J = 8.4 Hz, 1H), 7.78 – 7.71 (d, 2H), 7.50 – 7.43 (d, 2H), 4.66 (s, 2H), 2.52 (s, 6H).

**$^{13}$C NMR** (101 MHz, MeOD-$d_4$) δ 154.2, 144.5, 131.8, 128.5, 123.3, 122.8, 64.8.

**IR**: 3201, 3121, 2981, 2889, 1698, 1598, 1500, 1408, 1253, 1204, 1148, 1009, 971, 837, 765, 552, 523 $cm^{-1}$

**HRMS:** m/z $[M+H]^+$ calculated for $C_{12}H_{15}N_4O$ 231.124474 . Measured: 231.1240 Δ-1.9ppm

## Azoarene **4**

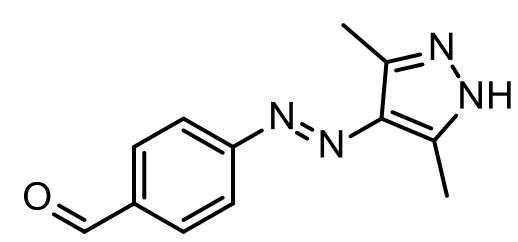

To a 40.0mL vial and stir bar was added (*E/Z*)-(4-((3,5-dimethyl-1H-pyrazol-4-yl)diazenyl)phenyl)methanol (200mg, 1.00 equiv., 869 μmol) and 10.0mL of DCM. DMP was added (3.66g, 9.93 equiv., 8.63mmol) and the reaction was stirred for 2h at room temperature. After completion, the reaction was diluted in DCM (50.0mL) then washed with saturated $Na_2SO_3$ (50.0mL x2) and saturated $NaHCO_3$ (50.0mL) and brine (50.0mL). The combined organic was dried over $Na_2SO_4$ then purified by flash chromatography on silica gel (CombiFlash, eluent: 2-6% ethyl acetate/hexanes) to yield (*E/Z*)-4-((3,5-dimethyl-1H-pyrazol-4-yl)diazenyl)benzaldehyde as an orange solid (62.0 mg, 31%). The m/z [M+H]+ was detected via LC-MS at $t_R$ 1.917 & 2.487 on a 3 min gradient of 5-95% MeCN in $H_2O$ (.1% formic acid).

**$R_f$** (3:2 ethyl actetate/hexanes): 0.30

**$^1$H NMR** (400 MHz, $CDCl_3$) δ 10.08 (s, 1H), 8.00 (d, *J* = 8.0 Hz, 2H), 7.92 (d, *J* = 8.1 Hz, 2H), 2.61 (s, 6H).

$^{13}$**C NMR** (126 MHz, $CDCl_3$) δ 191.7, 157.0, 142.3, 141.7, 136.5, 135.4, 134.1, 133.1, 132.7, 131.6, 130.7, 128.0, 127.9, 122.8, 122.4, 120.3, 12.3.

**IR**: 2981, 1589, 1439, 1382, 1234, 1141, 999, 846, 732, 561 $cm^{-1}$

**HRMS:** m/z $[M+H]^+$ calculated for C12H13N4O 229.108387. Measured: 229.1086 Δ-0.9.ppm

Azoarene **5**

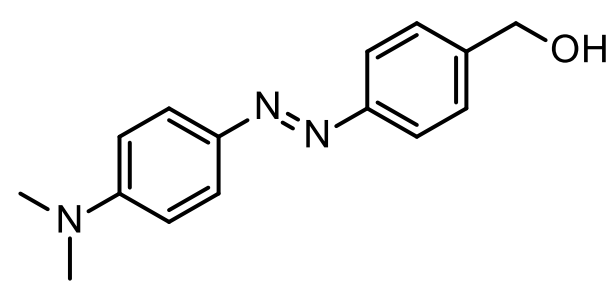


To a 50.0mL flask and stir bar was added (4-aminophenyl)methanol (203 mg, 1.00 equiv., 1.65mmol) and 5.00mL 12N HCl. The flask was cooled to 0°C. In a separate vial sodium nitrite (137 mg, 1.20 equiv., 1.98 mmol) was dissolved in 1.00mL $H_2O$ and added to the flask dropwise. The reaction was stirred for 2h at 0°C. N,N-dimethylaniline (209uL, 1.00 equiv., 1.65mmol) was added and the solution was stirred at room temperature for 30 mins. The solution was treated with 20.0mL saturated NaOAc and was then separated with ethyl acetate (200mL). The organic was collected, dried over $Na_2SO_4$, then concentrated under reduced pressure. A silica column from 30-60% ethyl acetate in hexanes yields (*E/Z*)-(4-((4-(dimethylamino)phenyl)diazenyl)phenyl)methanol as an orange solid (281 mg, 67%). The m/z 256.100 [M+H]+ was detected via LC-MS at $t_R$ 4.749 & 5.241 on a 6 min gradient of 5-100% MeCN in $H_2O$ (.1% formic acid).

$\mathbf{R_f}$ (30:70 EA in Hex): 0.30

$^1$**H NMR** (400 MHz, $CDCl_3$) δ 7.92 – 7.80 (m, 4H), 7.52 – 7.43 (m, 2H), 6.80 – 6.73 (m, 2H), 4.76 (d, *J* = 5.9 Hz, 2H), 3.09 (s, 6H).

$^{13}$**C NMR** (151 MHz, $CDCl_3$) δ 152.9, 152.6, 143.8, 142.2, 127.6, 125.1, 122.6, 111.7, 65.3, 40.5.

**IR**: 2981, 1602, 1526, 1375, 1232, 1157, 945, 826 $cm^{-1}$

**HRMS:** m/z $[M+H]^+$ calculated for $C_{15}H_{18}N_3O$ 256.144439. Measured: 256.1447 Δ-0.9ppm

## Azoarene **6**

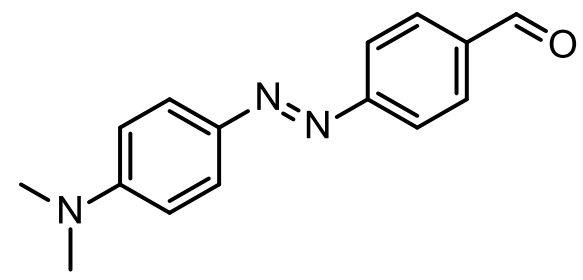

To a 20.0mL vial and stir bar was added (*E/Z*)-(4-((4-(dimethylamino)phenyl) diazenyl)phenyl)methanol (200mg, 1.00 equiv., 783 µmol) and 10.0mL of anhydrous DCM (76.0 mM). DMP was added (498mg, 1.50 equiv., 1.17mmol) and the reaction was stirred for 2h at room temperature. After completion, the reaction was diluted in EtOAc (50.0mL) then washed with saturated $Na_2SO_3$ (50.0mL x2) and saturated $NaHCO_3$ (50.0mL) and brine (50.0mL). The combined organic was dried over $Na_2SO_4$ then purified by flash chromatography on silica gel (CombiFlash, eluent: 0-40% ethyl acetate/hexanes) to yield (*E/Z*)-4-((4-(dimethylamino)phenyl)diazenyl)benzaldehyde as an orange solid (50.0 mg, 25%). The m/z 254.100 [M+H]+ was detected via LC-MS at $t_R$ 5.872 on a 6 min gradient of 5-100% MeCN in $H_2O$ (.1% formic acid).

**$R_f$** (1:4 ethyl acetate/hexanes): 0.30

**$^1$H NMR** (600 MHz, $CDCl_3$) δ 10.06 (s, 1H), 8.01 – 7.97 (m, 2H), 7.95 (d, *J* = 8.5 Hz, 2H), 7.93 – 7.90 (m, 2H), 6.79 – 6.75 (m, 2H), 3.13 (s, 6H).

**$^{13}$C NMR** (151 MHz, $CDCl_3$) δ 191.9, 157.1, 153.2, 144.0, 136.3, 130.9, 125.9, 122.8, 111.6, 40.4.

**IR**: 2981, 1553, 1379, 1157, 966 $cm^{-1}$

**HRMS:** m/z $[M+H]^+$ calculated for $C_{15}H_{16}N_3O$ 254.128789. Measured: 254.1287 Δ0.2ppm

## Azoarene **7**

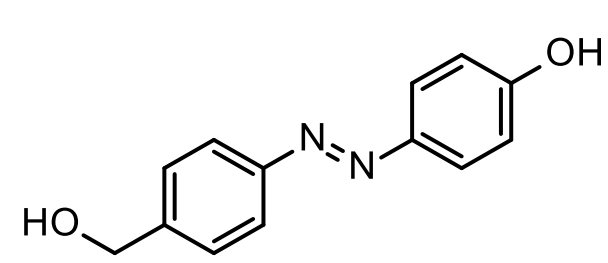

To a 25.0mL flask and stir bar was added 4-aminobenzylalcohol (400mg, 1.00 equiv., 3.25 mmol) and 4.40mL mq$H_2O$. The solution is stirred and cooled to 0°C. 12 N HCl (700µL, 2.50 equiv., 8.12 mmol) is added. In a separate vial a solution of 1.20mL mq$H_2O$ and sodium nitrite (235 mg, 1.05 equiv., 3.41 mmol) is prepared and cooled to 0°C. The $NaNO_2$ solution is added dropwise to the flask and the reaction is stirred at 0°C for 1h. A separate solution of phenol (321 mg, 1.05 equiv., 3.41 mmol) and $K_2CO_3$ (1.12g, 2.5 equiv., 8.12 mmol) is made in 4.00 mL mq$H_2O$ then added to the original solution dropwise. (Note: if the product

precipitates, add water to resolubilize). Stir 30 mins at 0°C then remove from the ice bath. A solution of 2:1 mq$H_2O$/99% glacial acetic acid, v/v is then added until a pH of 5 is reached (4.80mL was needed). The precipitated product was vacuum filtered and the residual solution was extracted with EtOAc (3x 20.0 mL). The combined organic was dried over $Na_2SO_4$ then purified by flash chromatography on silica gel (CombiFlash, eluent: 50% ethyl acetate/hexanes) to yield (*E/Z*)-4-((4-(hydroxymethyl)phenyl)diazenyl)phenol as an orange solid (510 mg, 69%). The m/z 229.532 [M+H]+ was detected via LC-MS at $t_R$ 1.750 on a 3 min gradient of 5-95% MeCN in $H_2O$ (.1% formic acid).

**$R_f$** (1:1 ethyl acetate/hexanes): 0.40

**$^1$H NMR** (400 MHz, MeOD) δ 7.85 – 7.77 (m, 4H), 7.50 (d, *J* = 8.3 Hz, 2H), 6.95 – 6.87 (m, 2H), 4.68 (s, 1H), 4.59 (br s, 1H).

**$^{13}$C NMR** (101 MHz, MeOD) δ 162.1, 153.4, 147.5, 145.3, 128.5, 125.9, 123.4, 116.7, 64.8.

**IR**: 2981, 1589, 1439, 1382, 1234, 1141, 999, 846, 732, 561cm$^{-1}$

**HRMS:** m/z $[M+H]^+$ calculated for $C_{13}H_{13}N_2O_2$ 229.097154. Measured: 229.0976 Δ-1.7ppm

## Azoarene **8**

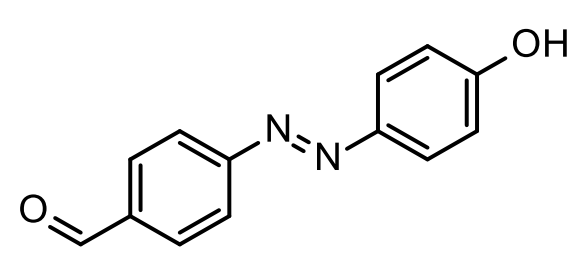


To a flame-dried flask and stir bar under argon was added (*E/Z*)-4-((4-(hydroxymethyl)phenyl)diazenyl)phenol (250 mg, 1.00 equiv., 1.10 mmol) and Celite (500mg, 7.60 equiv). Anhydrous THF is added, and the solution is stirred. PCC (375 mg, 1.59 equiv., 1.74mmol) is added and the solution is stirred for 1h at room temperature. The reaction was monitored by TLC. Once complete, the reaction mixture was filtered through 1:1 celite/silica twice with methanol as an eluent. The crude organic was purified by flash chromatography on silica gel (CombiFlash, eluent: 0-5% methanol/DCM) to yield (*E/Z*)-4-((4-hydroxyphenyl)diazenyl)benzaldehyde as an orange solid (53 mg, 21%). The m/z 227.139 [M+H]+ was detected via LC-MS at $t_R$ 2.066 on a 3 min gradient of 5-95% MeCN in $H_2O$ (.1% formic acid).

**$R_f$** (3:97 MeOH/DCM): 0.35

**$^1$H NMR** (400 MHz, MeOD) δ 10.09 (s, 1H), 8.06 – 7.89 (m, 6H), 7.02 – 6.93 (m, 2H).

**$^{13}$C NMR** (126 MHz, MeOD) δ 192.3, 162.0, 156.4, 146.2, 136.9, 130.5, 125.4, 122.7, 115.8.

**IR**: 2981, 1684, 1589, 1463, 1381, 1238, 1141, 958, 838, 543 cm$^{-1}$

**HRMS:** m/z [M+H]$^+$ calculated for $C_{13}H_{11}N_2O_2$ 227.081504. Measured: 227.0815 Δ-0.1ppm

## Compound 9 (**AzoCort1**) & Compound 9β (**AzoCort1β**)

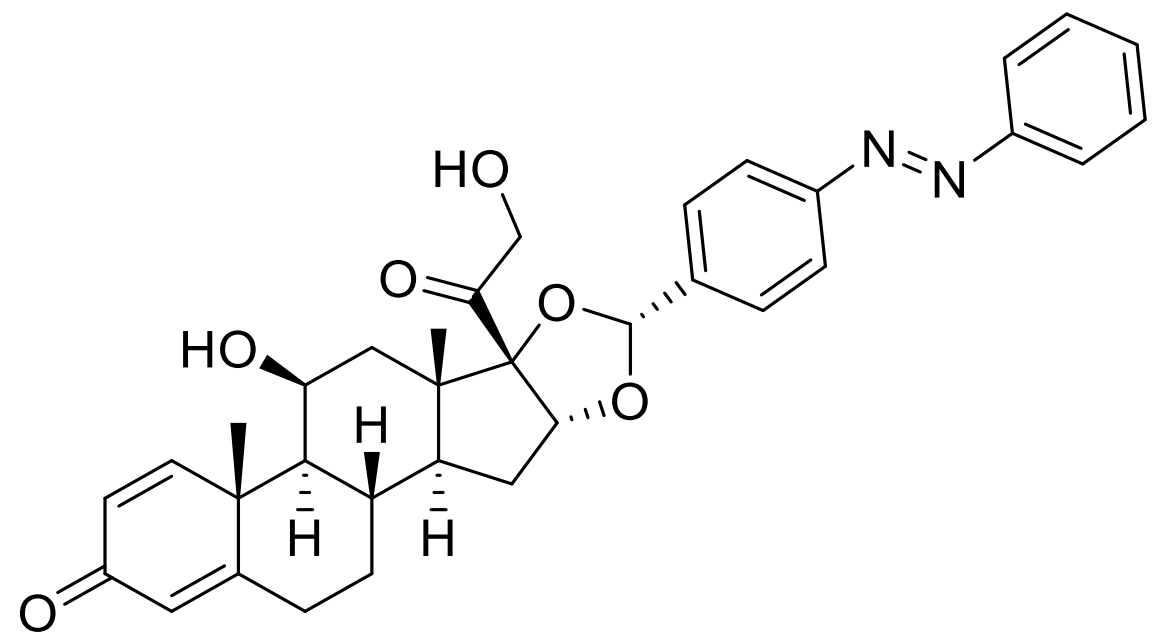

Prepared according to **General Procedure A** using 16α-hydroxyprednisolone (100mg, 1.00 equiv, 0.27mmol) and (*E*)-4-(phenyldiazenyl)benzaldehyde (61.4mg, 1.1 equiv., 0.29mmol). The compounds were purified via HPLC using method A (see HPLC Purification Methods). The compounds were isolated separately at $t_r$ = 6.61 (C22α) and 7.32 (C22β) as orange solids (36%, 54.0mg) in a 2:1 ratio favoring C22α, AzoCort1. The C22 epimer was determined via 2D NMR focusing on the NOESY correlations of the C22 acetal (α= 5.51, β=6.19) to the 20-oxo-21β-hydroxy AB system protons.

**$R_f$** (4:96 MeOH/DCM): 0.40

**$^1$H NMR** (400 MHz, $CDCl_3$) δ 7.92 (ddt, *J* = 9.7, 8.0, 1.8 Hz, 4H), 7.59 – 7.43 (m, 5H), 7.23 (d, *J* = 10.0 Hz, 1H), 6.03 (d, *J* = 1.7 Hz, 1H), 5.51 (s, 1H), 5.14 (d, *J* = 5.0 Hz, 1H), 4.67 (d, *J* = 20.1 Hz, 1H), 4.49 (q, *J* = 3.3 Hz, 1H), 4.33 (d, *J* = 20.2 Hz, 1H), 2.36 (ddd, J = 13.5, 5.0, 2.1 Hz, 1H), 2.28 – 2.02 (m, 3H), 2.01 – 1.91 (m, 1H), 1.75 (dddd, *J* = 33.8, 16.7, 9.5, 3.7 Hz, 4H), 1.45 (s, 3H), 1.29 – 1.06 (m, 4H), 0.98 (s, 3H).

**$^{13}$C NMR** (101 MHz, $CDCl_3$) δ 209.6, 186.6, 169.6, 155.9, 153.6, 152.5, 137.6, 131.4, 130.7, 129.2, 128.1, 127.5, 123.1, 123.0, 122.7, 120.6, 103.5, 97.8, 82.8, 69.9, 67.4, 64.5, 55.1, 50.3, 46.2, 44.0, 41.0, 34.0, 33.5, 31.9, 30.5, 25.4, 21.1, 17.4.

**IR**: 3424, 2935, 1717, 1656, 1616, 1442, 1391, 1297, 1238, 1168, 1062, 1014, 887, 841, 769, 689cm$^{-1}$

**HRMS:** m/z [M+H]$^+$ calculated for $C_{34}H_{37}N_2O_6$ 569.264613. Measured: 569.2655 Δ-1.5ppm

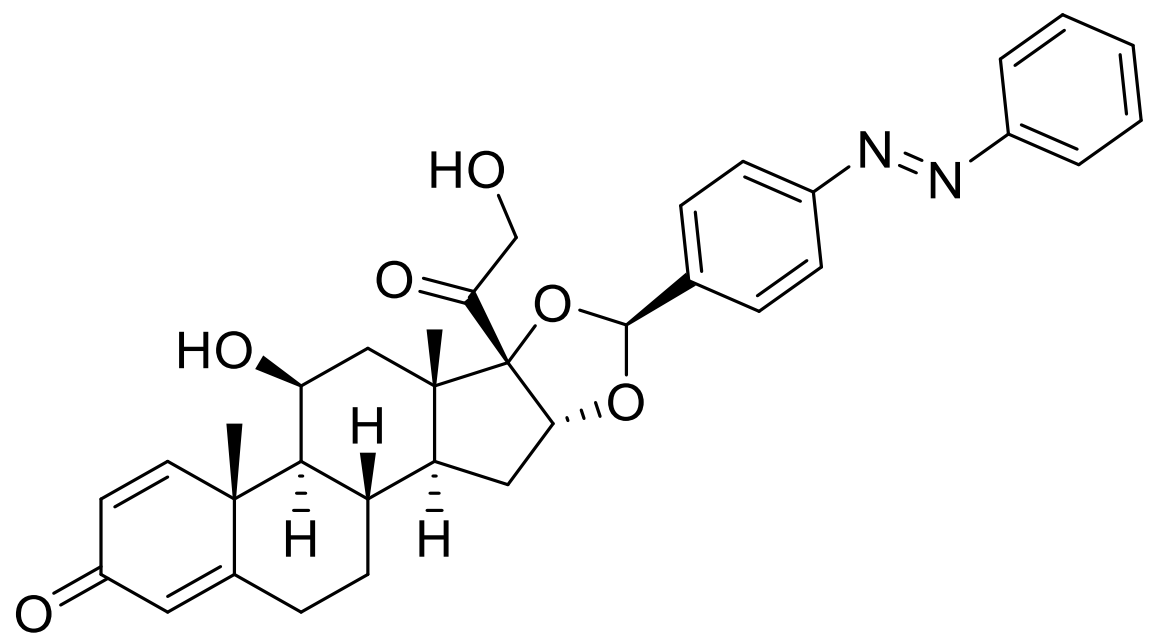

AzoCort1β (C22β) (contains <10% C22α):

**$R_f$** (4:96 MeOH/DCM): 0.40

**$^1$H** NMR (400 MHz, $CDCl_3$) δ 7.90 (ddd, *J* = 8.3, 6.5, 1.8 Hz, 4H), 7.59 – 7.40 (m, 6H), 7.23 (s, 1H), 6.32 – 6.24 (m, 1H), 6.05 (q, *J* = 2.2 Hz, 1H), 5.53 – 5.43 (m, 1H), 4.52 (q, *J* = 3.3 Hz, 1H), 4.38 – 4.25 (m, 1H), 4.09 (d, *J* = 19.9 Hz, 1H), 2.84 (s, 1H), 2.60 (tdd, *J* = 13.3, 5.3, 1.5 Hz, 1H), 2.38 (ddd, *J* = 13.3, 4.7, 1.9 Hz, 1H), 2.24 – 2.06 (m, 3H), 2.01 – 1.84 (m, 2H), 1.83 – 1.63 (m, 2H), 1.46 (d, *J* = 3.0 Hz, 3H), 1.31 – 1.07 (m, 3H), 1.01 (s, 3H).

**$^{13}$C NMR** (101 MHz, $CDCl_3$) δ 208.6, 186.5, 169.4, 155.7, 153.2, 152.5, 139.8, 131.4, 130.7, 129.2, 128.1, 126.9, 123.1, 123.0, 122.7, 106.6, 98.2, 84.3, 77.4, 77.0, 76.7, 69.9, 67.3, 55.2, 52.5, 47.5, 44.0, 41.3, 34.0, 33.2, 31.9, 31.0, 21.1, 17.7.

**IR**: 3436, 2981, 1657, 1442, 1391, 1165, 1090, 887, 768, 689 $cm^{-1}$

**HRMS:** m/z $[M+H]^+$ calculated for $C_{34}H_{37}N_2O_6$ 569.264613. Measured: 569.2654 Δ-1.5ppm

## Compound 10 (**AzoCort2**) & Compound 10β (**AzoCort2β**)

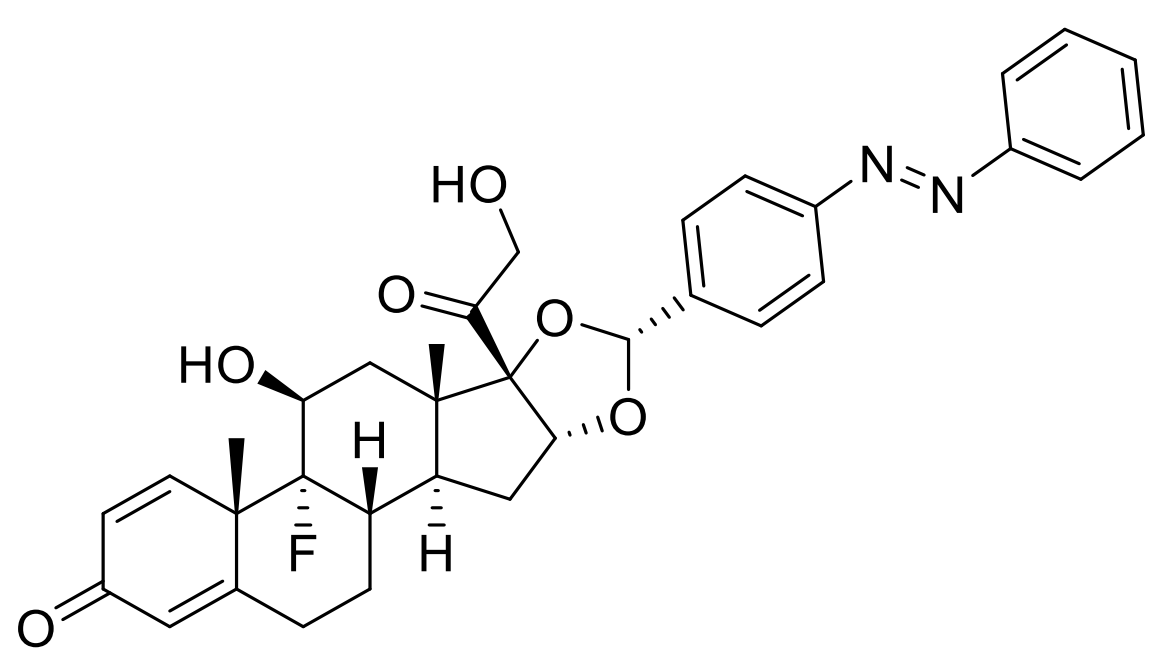

Prepared according to **General Procedure A** using triamcinolone (50.0mg, 1.00 equiv, 0.13 mmol) and (*E*)-4-(phenyldiazenyl)benzaldehyde (29.3mg, 1.10 equiv., 0.14mmol). The compounds were purified via HPLC using method B (see HPLC Purification Methods). The compounds were isolated separately at $t_r$ = 3.28+4.94 (C22α) and 5.38 (C22β) as orange solids (39%, 29.1mg) in a 4:1 ratio favoring C22α, AzoCort2. The C22 epimer was determined via 2D NMR focusing on the NOESY correlations of the C22 acetal (α= 5.57, β=6.28) to the 20-oxo-21β-hydroxy AB system protons.

**$R_f$** (4:96 MeOH/DCM): 0.40

**$^1$H NMR** (600 MHz, MeOD) δ 7.93 – 7.88 (m, 4H), 7.66 – 7.60 (m, 2H), 7.56 – 7.48 (m, 3H), 7.37 (dd, *J* = 16.0, 9.2 Hz, 1H), 6.30 (dd, *J* = 10.1, 1.9 Hz, 1H), 6.11 (t, *J* = 1.7 Hz, 1H), 5.58 (s, 1H), 5.49 (s, 1H), 5.10 (d, *J* = 5.3 Hz, 1H), 4.67 (d, *J* = 19.4 Hz, 1H), 4.36 (d, *J* = 19.4 Hz, 1H), 4.32 (dt, *J* = 9.6, 2.9 Hz, 1H), 2.77 – 2.69 (m, 1H), 2.60 (dtd, *J* = 28.8, 11.9, 4.8 Hz, 1H), 2.45 – 2.32 (m, 2H), 2.30 (dt, *J* = 13.9, 3.5 Hz, 1H), 2.03 (s, 1H), 1.93 (dt, *J* = 13.2, 5.2 Hz, 1H), 1.82 (dd, *J* = 13.5, 6.0 Hz, 1H), 1.74 (tdd, *J* = 13.9, 5.4, 1.9 Hz, 2H), 1.59 (s, 3H), 1.56 (d, *J* = 12.4 Hz, 1H), 1.01 (s, 3H).

**$^{13}$C NMR** (151 MHz, MeOD) δ 13C NMR (151 MHz, MeOD) δ 210.5, 189.5, 171.0, 156.1, 155.5, 154.6, 140.7, 133.2, 130.9, 130.6, 129.6, 129.3, 126.0, 124.6, 124.5, 122.1, 122.1, 105.2, 103.3, 102.1, 99.9, 84.4, 73.3, 73.0, 68.4, 55.4, 50.7, 50.6, 47.5, 45.6, 38.6, 35.2, 35.1, 34.7, 32.7, 29.7, 24.3, 24.3, 18.1.

**IR**: 3417, 2971, 1718, 1663, 1443, 1391, 1165, 1064, 980, 889, 770, 689 $cm^{-1}$

**HRMS:** m/z $[M+H]^+$ calculated for $C_{34}H_{36}FN_2O_6$ 587.255191. Measured: 587.2551 Δ0.1ppm

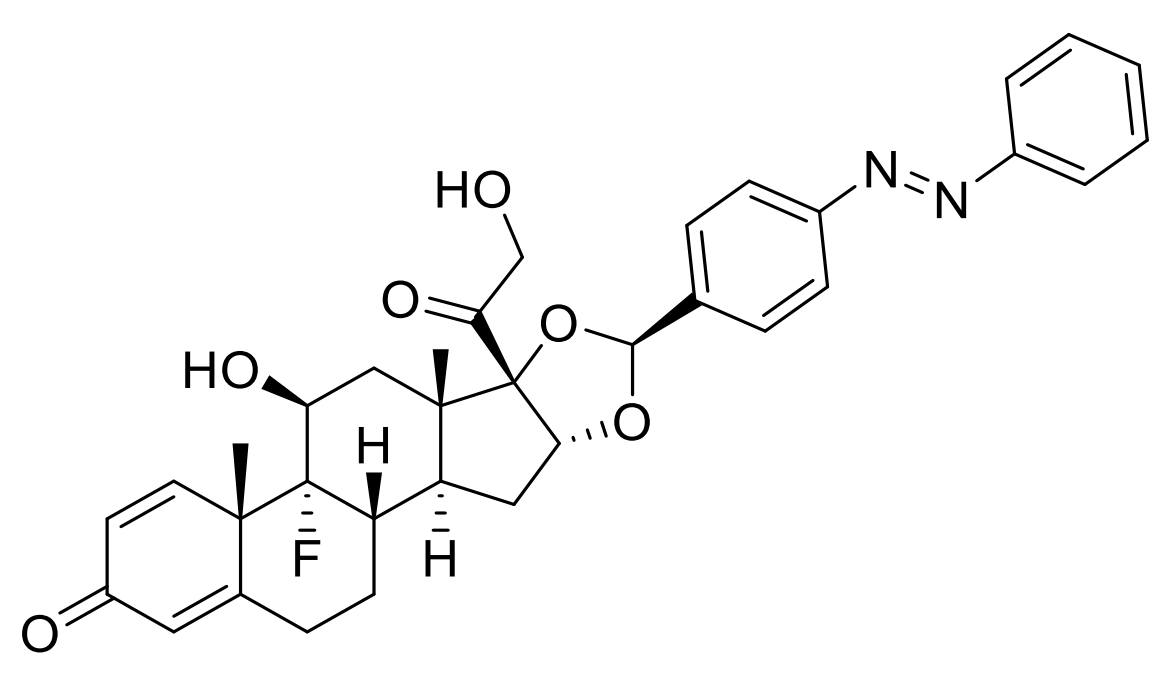

AzoCort2β (C22β):

**$R_f$** (4:96 MeOH/DCM): 0.40

**¹H NMR** (400 MHz, MeOD) δ 7.96 – 7.86 (m, 4H), 7.60 – 7.49 (m, 5H), 7.41 (d, *J* = 10.1 Hz, 1H), 6.31 (dt, *J* = 10.0, 2.8 Hz, 1H), 6.27 (s, 1H), 6.12 (t, *J* = 1.8 Hz, 1H), 5.47 (d, *J* = 6.6 Hz, 1H), 4.38 – 4.28 (m, 2H), 4.15 (d, *J* = 19.2 Hz, 1H), 2.77 (td, *J* = 13.7, 6.1 Hz, 1H), 2.58 (dtd, *J* = 28.8, 11.8, 4.8 Hz, 1H), 2.45 (dd, *J* = 13.7, 4.6 Hz, 1H), 2.35 – 2.15 (m, 2H), 2.02 – 1.79 (m, 2H), 1.76 – 1.57 (m, 5H), 1.03 (s, 3H).

**¹³C NMR** (151 MHz, MeOD) δ 207.3, 187.5, 169.1, 154.1, 153.1, 152.6, 140.9, 137.3, 131.1, 128.9, 128.6, 128.6, 127.5, 127.0, 126.7, 123.9, 122.5, 122.5, 122.4, 120.2, 120.2, 106.5, 106.3, 101.2, 100.1, 98.5, 98.4, 83.8, 83.8, 71.3, 71.0, 66.1, 66.1, 48.8, 48.6, 48.2, 46.5, 46.5, 45.6, 36.9, 33.9, 33.7, 32.3, 31.7, 30.7, 29.4, 29.1, 27.5, 22.3, 22.3, 22.2, 16.2, 16.2, 13.0.

**IR**: 2981, 1718, 1662, 1392, 1166, 1078, 893 $cm^{-1}$

**HRMS:** m/z $[M+H]^+$ calculated for $C_{34}H_{36}FN_2O_6$ 587.255191. Measured: 587.2551 Δ0.2ppm

Compound 11 (**AzoCort3**) & Compound 11β (**AzoCort3β**)

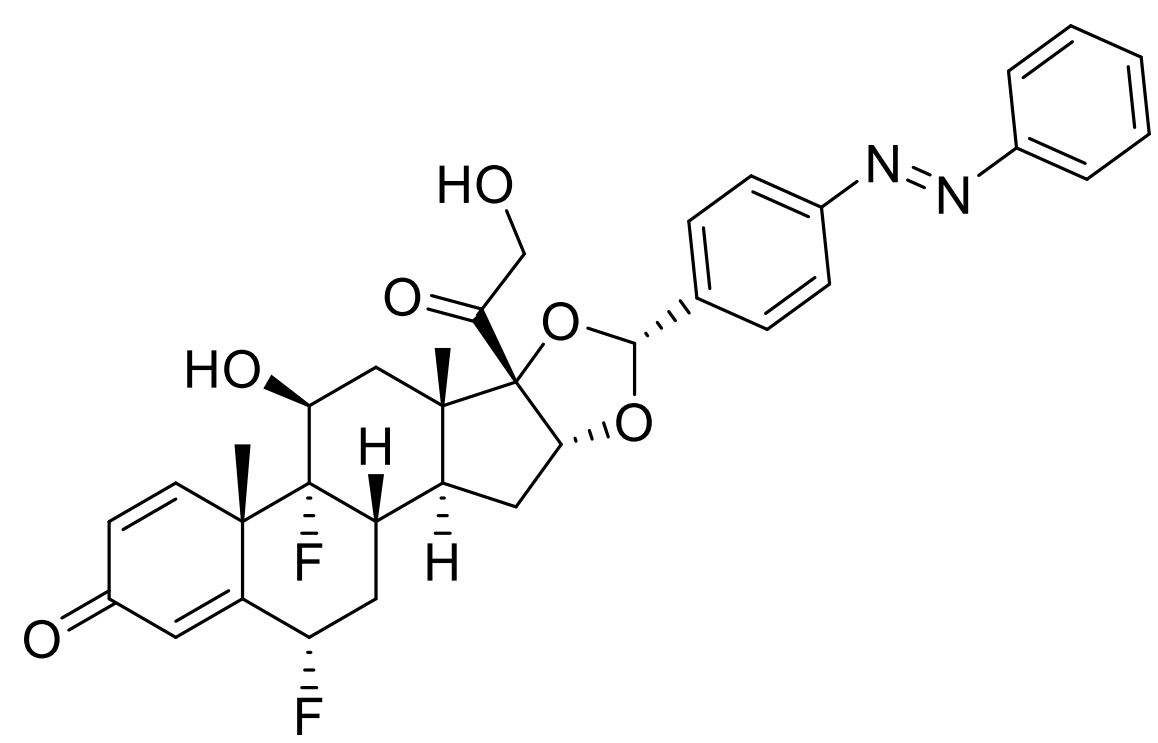


Prepared according to **General Procedure A** using fluocinolone acetonide (50.0mg, 1.00 equiv, 0.11 mmol) and (*E*)-4-(phenyldiazenyl)benzaldehyde (25.6mg, 1.10 equiv., 0.12mmol). The compounds were purified via HPLC using method C (see HPLC Purification Methods). The compounds were isolated separately at $t_r$ = 4.38 (C22α) and 4.74(C22β) as orange solids (45%, 30.1mg) in a 1.5:1 ratio favoring C22α, AzoCort3. The C22 epimer was determined via 2D NMR focusing on the NOESY correlations of the C22 acetal (α= 5.58, β=6.31) to the 20-oxo-21β-hydroxy AB system protons.

**$R_f$** (4:96 MeOH/DCM): 0.40

**¹H NMR** (600 MHz, MeOD) δ 7.94 – 7.88 (m, 3H), 7.65 – 7.61 (m, 2H), 7.56 – 7.48 (m, 2H), 7.37 – 7.29 (m, 2H), 6.87 – 6.78 (m, 1H), 6.37 – 6.31 (m, 2H), 5.59 (s, 1H), 5.54 – 5.46 (m,

1H), 5.08 (dd, *J* = 44.6, 5.0 Hz, 1H), 4.68 (d, *J* = 19.4 Hz, 1H), 4.39 – 4.25 (m, 2H), 2.76 – 2.62 (m, 1H), 2.43 (td, *J* = 12.6, 6.1 Hz, 1H), 2.38 (td, *J* = 6.9, 4.7 Hz, 1H), 2.30 (dq, *J* = 14.1, 3.1 Hz, 1H), 2.03 (s, 2H), 1.88 – 1.78 (m, 1H), 1.78 – 1.61 (m, 3H), 1.58 (s, 3H), 1.01 (s, 3H).

**$^{13}$C NMR** (151 MHz, MeOD) δ 13C NMR (151 MHz, MeOD) δ 209.7, 187.8, 165.8, 165.7, 156.1, 154.9, 154.8, 154.2, 153.9, 139.9, 136.5, 132.5, 130.3, 129.9, 128.9, 128.8, 128.6, 128.4, 123.9, 123.8, 121.5, 121.5, 121.1, 121.0, 118.1, 107.9, 104.6, 104.5, 101.5, 100.3, 99.8, 99.2, 99.1, 88.6, 87.4, 85.1, 83.6, 83.6, 72.4, 72.1, 67.8, 50.0, 49.9, 49.8, 49.8, 49.4, 49.3, 49.1, 49.0, 48.9, 48.7, 48.6, 47.9, 46.9, 44.8, 37.8, 35.5, 35.4, 34.0, 33.3, 33.2, 33.1, 33.1, 23.7, 23.6, 17.4, 17.3.

**IR**: 3423, 2971, 1718, 1668, 1630, 1392, 1294, 1068, 1015, 992, 941, 899, 841, 770, 689cm$^{-1}$

**HRMS:** m/z $[M+H]^+$ calculated for $C_{34}H_{35}F_2N_2O_6$ 605.245770. Measured: 605.2463 Δ-0.8ppm

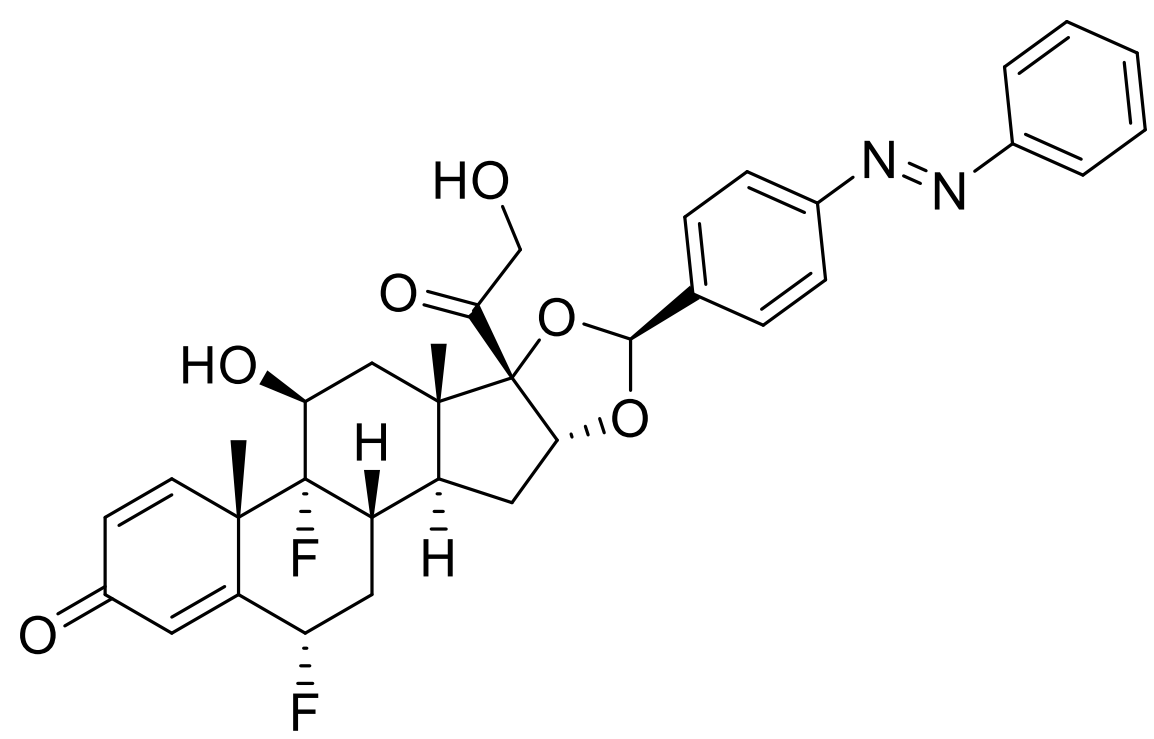


AzoCort3β (C22β):

**$R_f$** (4:96 MeOH/DCM): 0.40

**$^1$H NMR** (400 MHz, MeOD) δ 7.96 – 7.86 (m, 4H), 7.59 – 7.47 (m, 5H), 7.40 – 7.22 (m, 1H), 6.87 – 6.79 (m, 1H), 6.39 – 6.27 (m, 3H), 5.68 – 5.58 (m, 1H), 5.56 – 5.46 (m, 1H), 4.42 – 4.29 (m, 2H), 4.20 – 4.09 (m, 1H), 2.67 (ddd, *J* = 28.5, 12.4, 6.2 Hz, 1H), 2.35 (ddt, *J* = 28.0, 10.6, 6.3 Hz, 3H), 2.06 – 1.64 (m, 4H), 1.59 (d, *J* = 7.9 Hz, 3H), 1.02 (s, 3H).

**$^{13}$C NMR** (151 MHz, MeOD) δ 207.2, 186.5, 164.5, 164.4, 154.2, 153.1, 152.8, 152.6, 140.8, 137.3, 131.2, 128.9, 128.9, 128.8, 128.6, 127.6, 127.5, 127.0, 126.7, 122.5, 122.5, 122.5, 120.2, 120.2, 119.7, 119.6, 106.5, 106.3, 100.1, 98.9, 98.4, 98.3, 97.8, 87.2, 86.0, 83.7, 83.6, 82.3, 71.0, 70.8, 66.1, 66.1, 48.6, 48.5, 48.2, 46.5, 46.5, 45.3, 45.3, 36.8, 34.0, 33.9, 32.5, 32.4, 32.4, 32.3, 32.2, 29.4, 22.2, 22.2, 16.2.

**IR**: 2981, 1668, 1381, 1154, 1077, 969, 769 cm$^{-1}$

**HRMS:** m/z $[M+H]^+$ calculated for $C_{34}H_{35}F_2N_2O_6$ 605.245770. Measured: 605.2458 $\Delta$-0.1ppm

Compound 12 (**AzoCort4**) & Compound 12β (**AzoCort4β**)

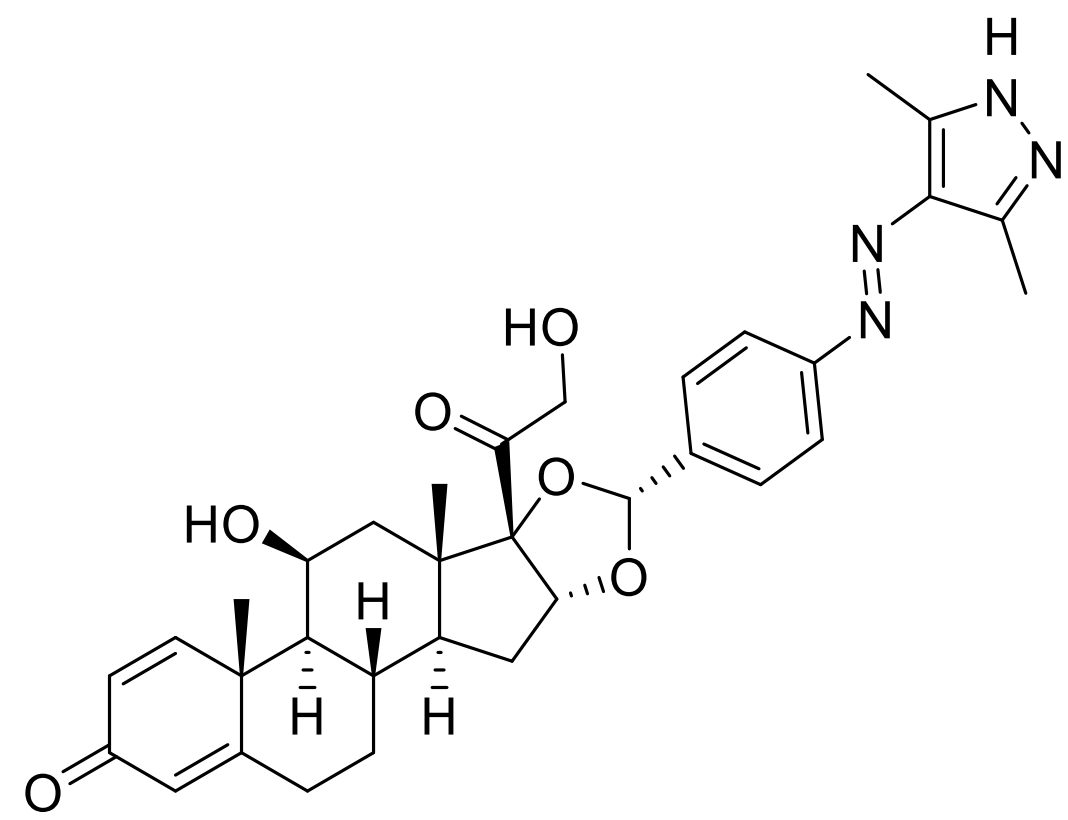

Prepared according to **General Procedure A** using 16α-hydroxyprednisolone (93.0mg, 1.00 equiv, 0.25mmol) and ((*E*)-4-((3,5-dimethyl-1H-pyrazol-4-yl)diazenyl)benzaldehyde (62.0mg, 1.10 equiv., 0.27mmol). The compounds were purified via HPLC using method D (see HPLC Purification Methods). The compounds were isolated separately at $t_r$ = 4.39 (C22α) and 4.99 (C22β) as orange solids (48%, 69.5mg) in a 10:1 ratio favoring C22α, AzoCort4. The C22 epimer was determined via 2D NMR focusing on the NOESY correlations of the C22 acetal (α= 5.97, β=6.21) to the 20-oxo-21β-hydroxy AB system protons.

**$R_f$** (70:30 EtOAc:Hex): 0.1

**$^1$H NMR** (400 MHz, MeOD) δ 7.73 (d, *J* = 8.3 Hz, 2H), 7.58 – 7.51 (m, 2H), 7.40 (d, *J* = 10.0 Hz, 1H), 6.21 (dd, *J* = 10.0, 1.9 Hz, 1H), 5.97 (t, *J* = 1.6 Hz, 1H), 5.51 (s, 1H), 5.08 (d, *J* = 5.3 Hz, 1H), 4.66 (d, *J* = 19.4 Hz, 1H), 4.41 – 4.29 (m, 2H), 2.62 (td, *J* = 13.7, 5.4 Hz, 1H), 2.50 (s, 7H), 2.38 – 2.29 (m, 1H), 2.22 (qd, *J* = 10.2, 5.7 Hz, 1H), 2.15 – 2.05 (m, 1H), 2.05 – 1.95 (m, 2H), 1.92 – 1.78 (m, 3H), 1.71 (td, *J* = 12.0, 5.6 Hz, 1H), 1.46 (s, 3H), 1.12 (qd, *J* = 13.6, 5.4 Hz, 1H), 1.02 (d, *J* = 3.5 Hz, 1H), 0.98 (s, 4H).

**$^{13}$C NMR** (101 MHz, MeOD) δ 208.6, 192.1, 187.5, 172.6, 158.2, 154.4, 136.9, 130.4, 127.4, 126.6, 121.4, 103.2, 98.1, 82.2, 69.0, 66.4, 55.4, 50.3, 45.8, 44.5, 40.1, 34.1, 33.1, 31.6, 30.4, 20.2, 16.4, 10.6.

**IR**: 2981, 1656, 1391, 1239, 1156, 1059, 957, 838 $cm^{-1}$

**HRMS:** m/z $[M+H]^+$ calculated for $C_{33}H_{39}N_4O_6$ 587.286411. Measured: 587.2862 Δ0.4ppm

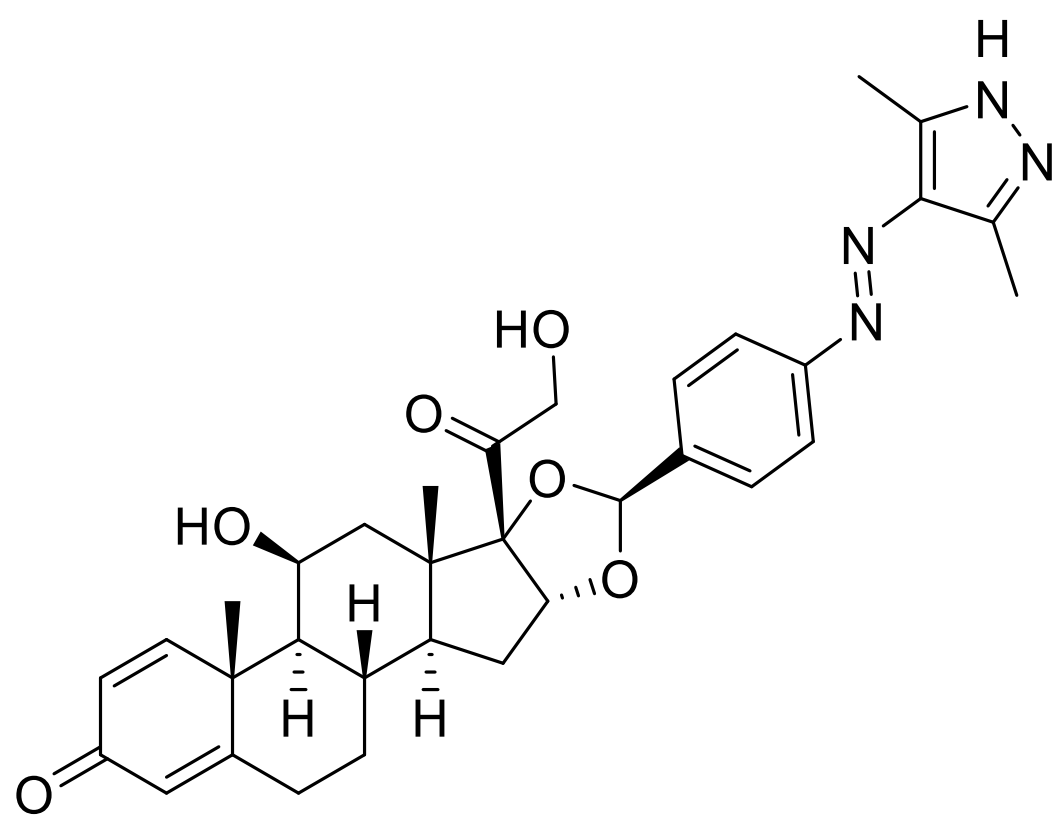

AzoCort4β (C22β):

**$R_f$** (70:30 EtOAc:Hex): 0.1

**$^1$H NMR** (400 MHz, MeOD) δ 7.81 – 7.70 (m, 2H), 7.61 – 7.54 (m, 1H), 7.50 – 7.40 (m, 2H), 6.30 – 6.19 (m, 1H), 6.03 (dt, *J* = 6.2, 1.6 Hz, 1H), 5.54 (s, 1H), 5.47 – 5.41 (m, 1H), 5.09 (d, *J* = 5.2 Hz, 1H), 4.66 (d, *J* = 19.3 Hz, 1H), 4.58 (s, 1H), 4.44 (d, *J* = 3.4 Hz, 1H), 4.37 (d, *J* = 6.9 Hz, 1H), 4.32 (d, *J* = 6.8 Hz, 1H), 4.14 (d, *J* = 19.2 Hz, 1H), 2.67 (dq, *J* = 12.8, 6.7 Hz, 1H), 2.51 (s, 6H), 2.45 – 2.37 (m, 1H), 2.34 – 2.13 (m, 1H), 2.10 – 1.97 (m, 1H), 1.97 – 1.67 (m, 4H), 1.51 (d, *J* = 2.4 Hz, 3H), 1.16 – 1.04 (m, 1H), 1.01 (d, *J* = 5.1 Hz, 3H).

**$^{13}$C NMR** (151 MHz, MeOD) δ 208.6, 187.6, 172.9, 158.4, 158.3, 154.5, 154.1, 136.9, 127.3, 126.9, 126.6, 121.4, 121.4, 121.3, 106.6, 103.3, 98.6, 98.1, 83.8, 82.3, 69.1, 66.4, 66.2, 55.7, 52.4, 50.4, 48.2, 47.0, 45.8, 44.6, 44.6, 40.1, 34.2, 34.0, 33.1, 32.7, 31.7, 31.0, 30.5, 29.4, 20.2, 16.7, 16.4.

**IR**: 2981, 1657, 1553, 1382, 1239, 1153, 958 $cm^{-1}$

**HRMS:** m/z $[M+H]^+$ calculated for $C_{33}H_{39}N_4O_6$ 587.2864. Measured: 587.285287 Δ1.9ppm

Compound 13 (**AzoCort5,** 8:1 mixture C22α: C22β)

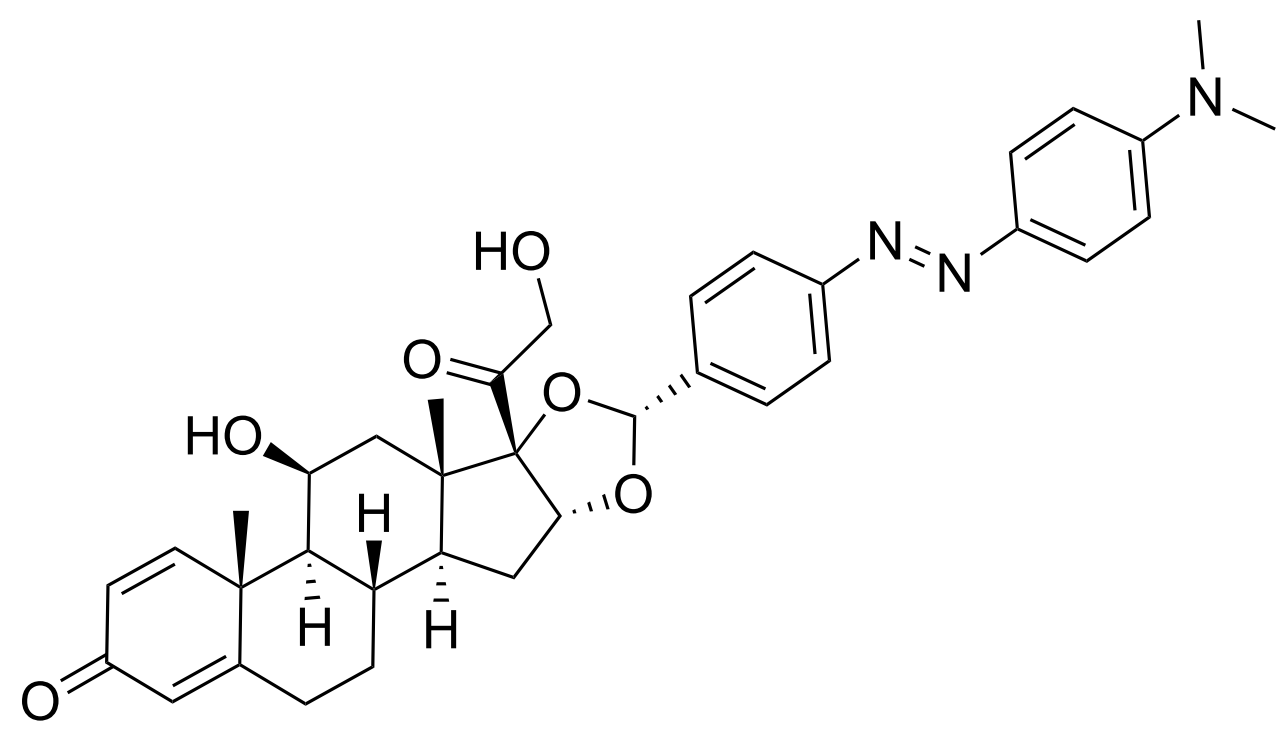


Prepared according to **General Procedure A** using 16α-hydroxyprednisolone (60.8mg, 1.00 equiv, 0.16mmol) and (*E*)-4-((4-(dimethylamino)phenyl)diazenyl)benzaldehyde (45.0mg, 1.10 equiv., 0.18mmol). The crude material was purified via flash chromatography and due to solubility challenges were not purified via HPLC. The mixture of diastereomers were isolated as orange solids (70%, 69.5mg) in an 7.15:1 ratio favoring C22α, AzoCort5, via LCMS & NMR.

**$R_f$** (70:30 EtOAc:Hex): 0.30

**$^{1}$H NMR** (400 MHz, MeOD) δ 7.86 – 7.73 (m, 4H), 7.62 – 7.54 (m, 2H), 7.51 – 7.40 (m, 2H), 6.87 – 6.76 (m, 2H), 6.66 (d, *J* = 8.9 Hz, 1H), 6.30 – 6.19 (m, 2H), 6.03 (q, *J* = 2.7 Hz, 1H), 5.54 (s, 1H), 5.09 (d, *J* = 5.3 Hz, 1H), 4.66 (d, *J* = 19.4 Hz, 1H), 4.44 (d, *J* = 3.3 Hz, 2H), 4.35 (dd, *J* = 19.3, 3.7 Hz, 1H), 4.18 – 4.06 (m, 1H), 3.09 (s, 6H), 2.67 (td, *J* = 13.5, 5.3 Hz, 2H), 2.45 – 2.35 (m, 1H), 2.31 – 2.12 (m, 1H), 2.05 – 1.99 (m, 3H), 1.94 – 1.87 (m, 1H), 1.85 – 1.80 (m, 2H), 1.76 (dd, *J* = 12.0, 5.2 Hz, 1H), 1.53 – 1.46 (m, 4H), 1.24 (t, *J* = 7.1 Hz, 1H), 1.21 – 1.06 (m, 2H), 1.01 (d, *J* = 5.2 Hz, 4H), 0.94 – 0.86 (m, 1H).

**$^{13}$C NMR** (151 MHz, MeOD) δ 208.6, 187.5, 172.8, 158.3, 154.1, 153.1, 143.4, 136.7, 127.3, 126.9, 126.6, 124.8, 121.6, 121.3, 111.2, 103.3, 98.1, 82.3, 69.1, 66.4, 60.1, 55.6, 50.4, 48.2, 47.0, 45.8, 44.6, 40.1, 39.0, 34.1, 33.0, 31.7, 30.5, 29.4, 29.3, 29.1, 26.7, 22.3, 20.2, 16.4, 13.1.

**IR**: 2981, 1657, 1600, 1382, 1238, 1154, 957, 813 $cm^{-1}$

**HRMS:** m/z $[M+H]^+$ calculated for $C_{36}H_{42}N_3O_6$ 612.306813. Measured: 612.3064 Δ0.7ppm

Compound 14 (**AzoCort6**) & Compound 14β (**AzoCort6β**)

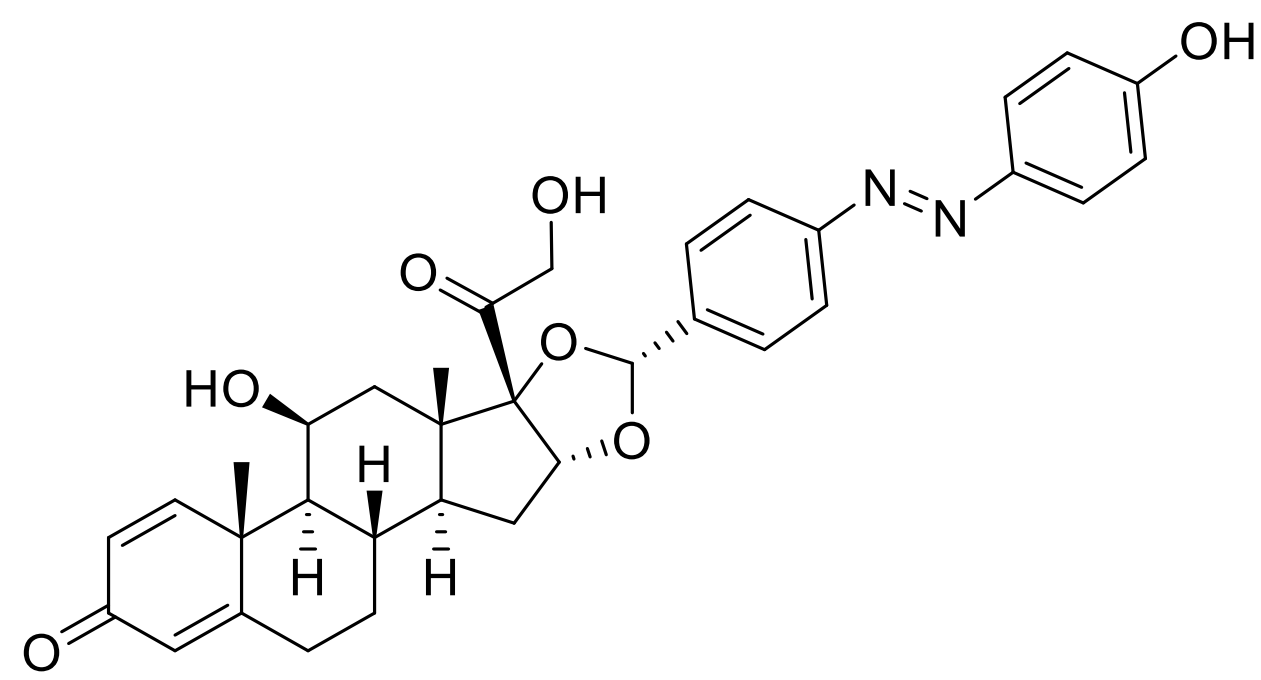

Prepared according to **General Procedure A** using 16α-hydroxyprednisolone (93.8mg, 1.00 equiv, 0.25mmol) and (*E*)-4-((4-hydroxyphenyl)diazenyl)benzaldehyde (62.0mg, 1.10 equiv., 0.27mmol). The compounds were purified via HPLC using method E (see HPLC Purification Methods). The compounds were isolated separately at $t_r$ = 3.63 (C22α) and 4.56 (C22β) as orange solids (37.1%, 54.1mg) in a 2.8:1 ratio favoring C22α, AzoCort6.

**$R_f$** (70:30 EtOAc:Hex): 0.30

**$^1$H NMR** (400 MHz, MeOD) δ 7.88 – 7.78 (m, 4H), 7.63 – 7.56 (m, 2H), 7.46 (s, 1H), 6.95 – 6.87 (m, 2H), 6.24 (dd, *J* = 10.1, 1.9 Hz, 1H), 6.02 (t, *J* = 1.6 Hz, 1H), 5.55 (s, 1H), 5.10 (d, *J* = 5.2 Hz, 1H), 4.64 (s, 1H), 4.47 – 4.36 (m, 2H), 2.73 – 2.61 (m, 1H), 2.39 (dd, *J* = 13.5, 3.6 Hz, 1H), 2.28 (qd, *J* = 11.2, 4.1 Hz, 1H), 2.06 – 1.97 (m, 1H), 1.95 – 1.67 (m, 4H), 1.50 (s, 3H), 1.28 – 1.09 (m, 1H), 1.08 (dd, *J* = 11.2, 3.6 Hz, 1H), 1.01 (s, 3H).

**$^{13}$C NMR** (151 MHz, MeOD) δ 208.6, 187.6, 172.8, 161.1, 158.3, 153.7, 146.1, 137.6, 127.4, 126.6, 124.8, 122.0, 121.3, 115.4, 103.2, 98.1, 82.3, 69.1, 66.4, 55.6, 50.4, 48.2, 45.8, 44.6, 40.1, 34.1, 33.0, 31.7, 30.5, 20.2, 16.4.

**IR**: 2981, 1553, 1382, 1239, 1151, 957, 803cm$^{-1}$

**HRMS:** m/z $[M+H]^+$ calculated for $C_{34}H_{37}N_2O_7$ 585.259528. Measured: 585.2586 Δ1.7ppm

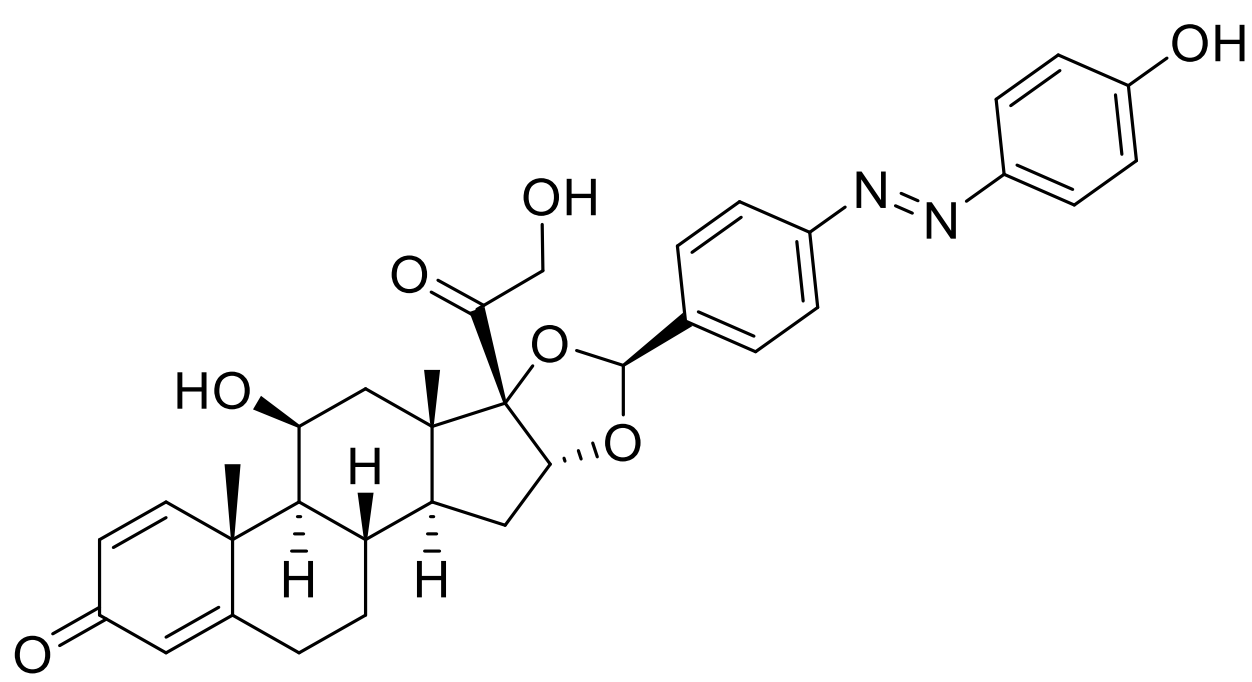


AzoCort6 C22β:

**R$_f$** (70:30 EtOAc:Hex): 0.30

**$^1$H NMR** (400 MHz, MeOD) δ 7.85 – 7.77 (m, 4H), 7.50 – 7.43 (m, 3H), 6.95 – 6.87 (m, 2H), 6.27 (dd, *J* = 10.0, 1.9 Hz, 1H), 6.22 (s, 1H), 6.04 (t, *J* = 1.6 Hz, 1H), 5.44 (dd, *J* = 5.4, 2.2 Hz, 1H), 4.44 (q, *J* = 3.2 Hz, 1H), 4.31 (s, 1H), 4.16 (s, 1H), 2.74 – 2.62 (m, 1H), 2.46 – 2.36 (m, 1H), 2.30 – 2.12 (m, 2H), 2.10 – 2.01 (m, 1H), 1.99 – 1.87 (m, 2H), 1.87 – 1.75 (m, 2H), 1.51 (s, 3H), 1.31 – 1.18 (m, 2H), 1.15 (dd, *J* = 11.1, 3.6 Hz, 1H), 1.02 (s, 3H).

**$^{13}$C NMR** (101 MHz, MeOD) δ 207.5, 187.5, 173.0, 161.0, 158.4, 153.3, 146.1, 139.9, 126.9, 126.6, 124.7, 122.0, 121.3, 115.4, 106.5, 98.7, 83.8, 69.0, 66.1, 55.7, 52.4, 44.6, 40.4, 34.0, 32.7, 31.7, 31.0, 20.2, 16.7.

**IR**: 2981, 1655, 1592, 1382, 1239, 1141, 1081, 957, 845 cm$^{-1}$

**HRMS:** m/z $[M+H]^+$ calculated for $C_{34}H_{37}N_2O_7$ 585.259528. Measured: 585.2599 Δ-0.7ppm

# Index of Spectra

## Azoarene 4


S 1 $^1$H NMR OF AZOARENE 4 (400MHZ, CDCL$_3$) ........ 30
S 2 $^{13}$C NMR OF AZOARENE 4 (101MHZ, CDCL$_3$) ........ 31


## AzoCort1


S 3. $^1$H NMR OF AZOCORT1(400MHZ, CDCL$_3$) ........ 32
S 4 $^{13}$C NMR OF AZOCORT1(101MHZ, CDCL$_3$) ........ 33
S 5 HSQC NMR OF AZOCORT1(400MHZ, CDCL$_3$) ........ 34
S 6 NOESY NMR OF AZOCORT1(400MHZ, CDCL$_3$) ........ 35
S 7 $^1$H NMR OF AZOCORT1β (400MHZ, CDCL$_3$) ........ 36
S 8 $^{13}$C NMR OF AZOCORT1β (101MHZ, CDCL$_3$) ........ 37
S 9 HSQC 2D NMR OF AZOCORT1β (400MHZ, CDCL$_3$) ........ 38
S 10 NOESY 2D NMR OF AZOCORT1β (400MHZ, CDCL$_3$) ........ 39


## AzoCort2


S 11 $^1$H NMR OF AZOCORT2 (600MHZ, MEOD) ........ 40
S 12 $^{13}$C NMR OF AZOCORT2 (151MHZ, MEOD) ........ 41
S 13 HSQC 2D NMR OF AZOCORT2 (600MHZ, MEOD) ........ 42
S 14 NOESY 2D NMR OF AZOCORT2 (600MHZ, MEOD) ........ 43
S 15 $^1$H NMR OF AZOCORT2β (600MHZ, MEOD) ........ 44
S 16 13C NMR OF AZOCORT2β (151MHZ, MEOD) ........ 45
S 17 NOESY 2D NMR OF AZOCORT2β (600MHZ, MEOD) ........ 46


## AzoCort3


S 18 $^1$H NMR OF AZOCORT3 (600MHZ, MEOD) ........ 47
S 19 $^{13}$C NMR OF AZOCORT3 (151MHZ, MEOD) ........ 48
S 20 HSQC NMR OF AZOCORT3 (600MHZ, MEOD) ........ 49
S 21 NOESY NMR OF AZOCORT3 (600MHZ, MEOD) ........ 50
S 22 $^1$H NMR OF AZOCORT3β (400MHZ, MEOD) ........ 51
S 23 $^{13}$C NMR OF AZOCORT3β (151MHZ, MEOD) ........ 52
S 24 NOESY NMR OF AZOCORT3β (400MHZ, MEOD) ........ 53


## AzoCort4


S 25 $^1$H NMR OF AZOCORT4 (400MHZ, MEOD) ........ 54
S 26 13C NMR OF AZOCORT4 (101MHZ, MEOD) ........ 55
S 27 HSQC NMR OF AZOCORT4 (400MHZ, MEOD) ........ 56
S 28 NOESY NMR OF AZOCORT4 (400MHZ, MEOD) ........ 57
S 29 1H NMR OF AZOCORT4β (400MHZ, MEOD) ........ 58
S 30 13C NMR OF AZOCORT4β (151MHZ, MEOD) ........ 59
S 31 NOESY NMR OF AZOCORT4β (400MHZ, MEOD) ........ 60

## AzoCort5


S 32 1H NMR OF AZOCORT5 (400MHZ, MEOD) ........ 61
S 33 13C NMR OF AZOCORT5 (151MHZ, MEOD) ........ 62
S 34 HSQC NMR OF AZOCORT5 (600MHZ, MEOD) ........ 63
S 35 NOESY NMR OF AZOCORT5 (600MHZ, MEOD) ........ 64


## AzoCort6


S 36 1H NMR OF AZOCORT6 (400MHZ, MEOD) ........ 65
S 37 13C NMR OF AZOCORT6 (151MHZ, MEOD) ........ 66
S 38 HSQC NMR OF AZOCORT6 (600MHZ, MEOD) ........ 67
S 39 NOESY NMR OF AZOCORT6 (600MHZ, MEOD) ........ 68
S 40 1H NMR OF AZOCORT6β (400MHZ, MEOD) ........ 69
S 41 13C NMR OF AZOCORT6β (101MHZ, MEOD) ........ 70
S 42 NOESY NMR OF AZOCORT6β (400MHZ, MEOD) ........ 71


## LC-MS Purity Traces


S 43 AZOCORT1 LC-MS PURITY TRACE ........ 72
S 44 AZOCORT2 LC-MS PURITY TRACE ........ 73
S 45 AZOCORT3 LC-MS PURITY TRACE ........ 74
S 46 AZOCORT4 LC-MS PURITY TRACE ........ 75
S 47 AZOCORT5 LC-MS PURITY TRACE ........ 76
S 48 AZOCORT6 LC-MS PURITY TRACE ........ 77


## Photocharacterization


S 49 AZOCORT1 PHOTOCHARACTERIZATION ........ 79
S 50 AZOCORT2 PHOTOCHARACTERIZATION ........ 80
S 51 AZOCORT3 PHOTOCHARACTERIZATION ........ 81
S 52 AZOCORT4 PHOTOCHARACTERIZATION ........ 82
S 53 AZOCORT5 PHOTOCHARACTERIZATION ........ 83
S 54 AZOCORT6 PHOTOCHARACTERIZATION ........ 84

*S 1 $^1$H NMR of Azoarene 4 (400MHz, $CDCl_3$)*

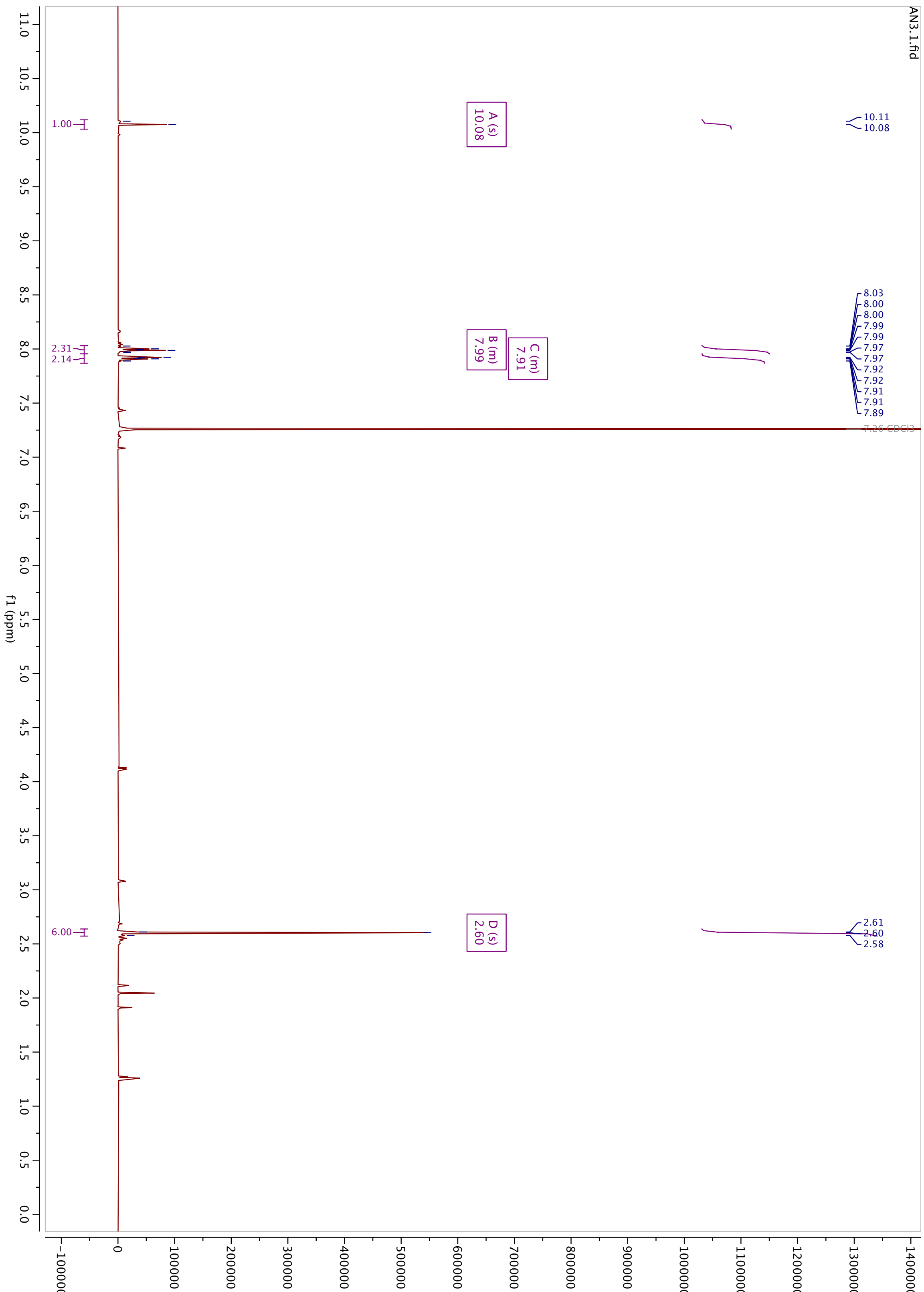

S 2 $^{13}C$ NMR of Azoarene 4 (101MHz, $CDCl_3$)

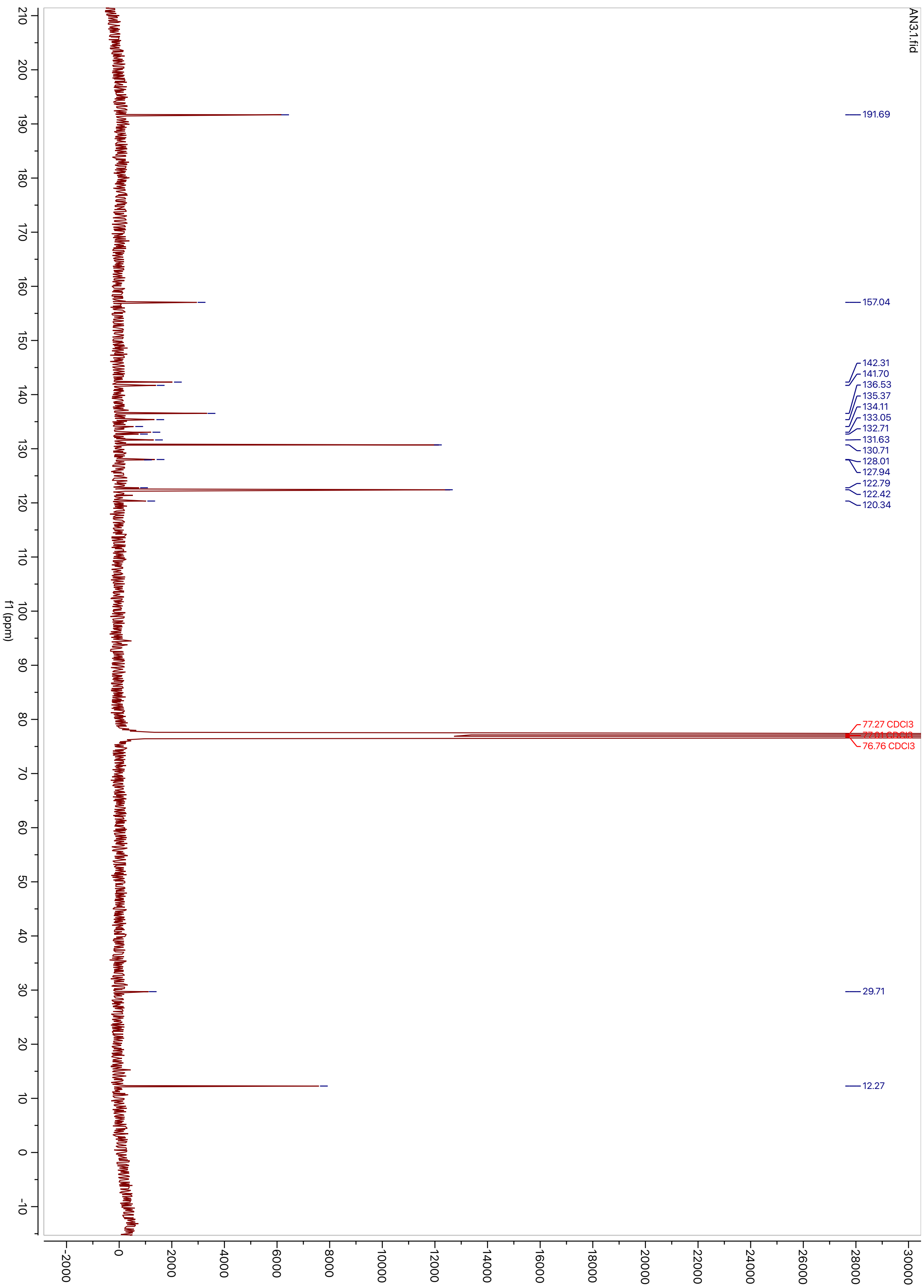

*S 3. ¹H NMR of AzoCort1(400MHz, CDCl₃)*

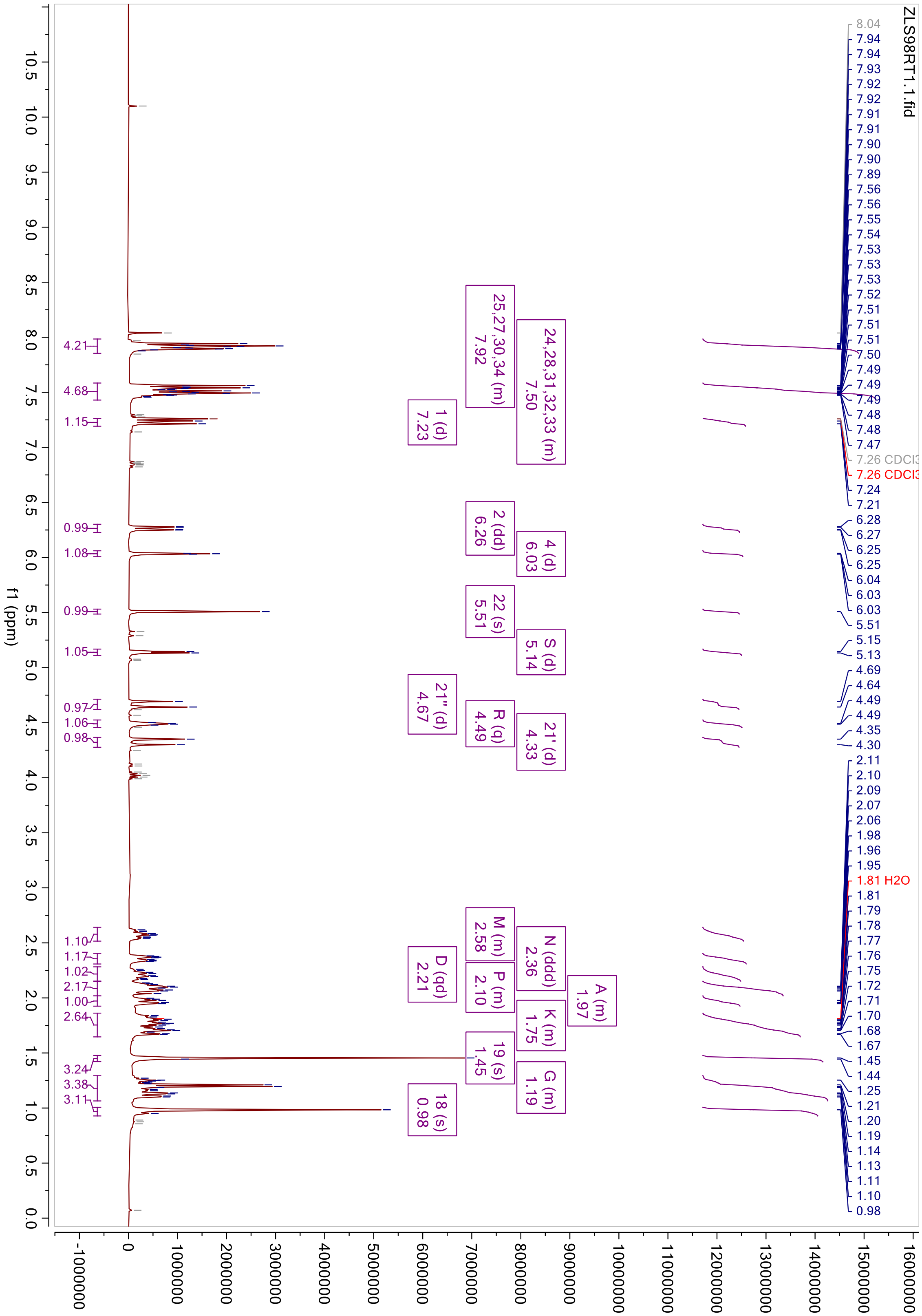

*S 4 $^{13}C$ NMR of AzoCort1(101MHz, $CDCl_3$)*

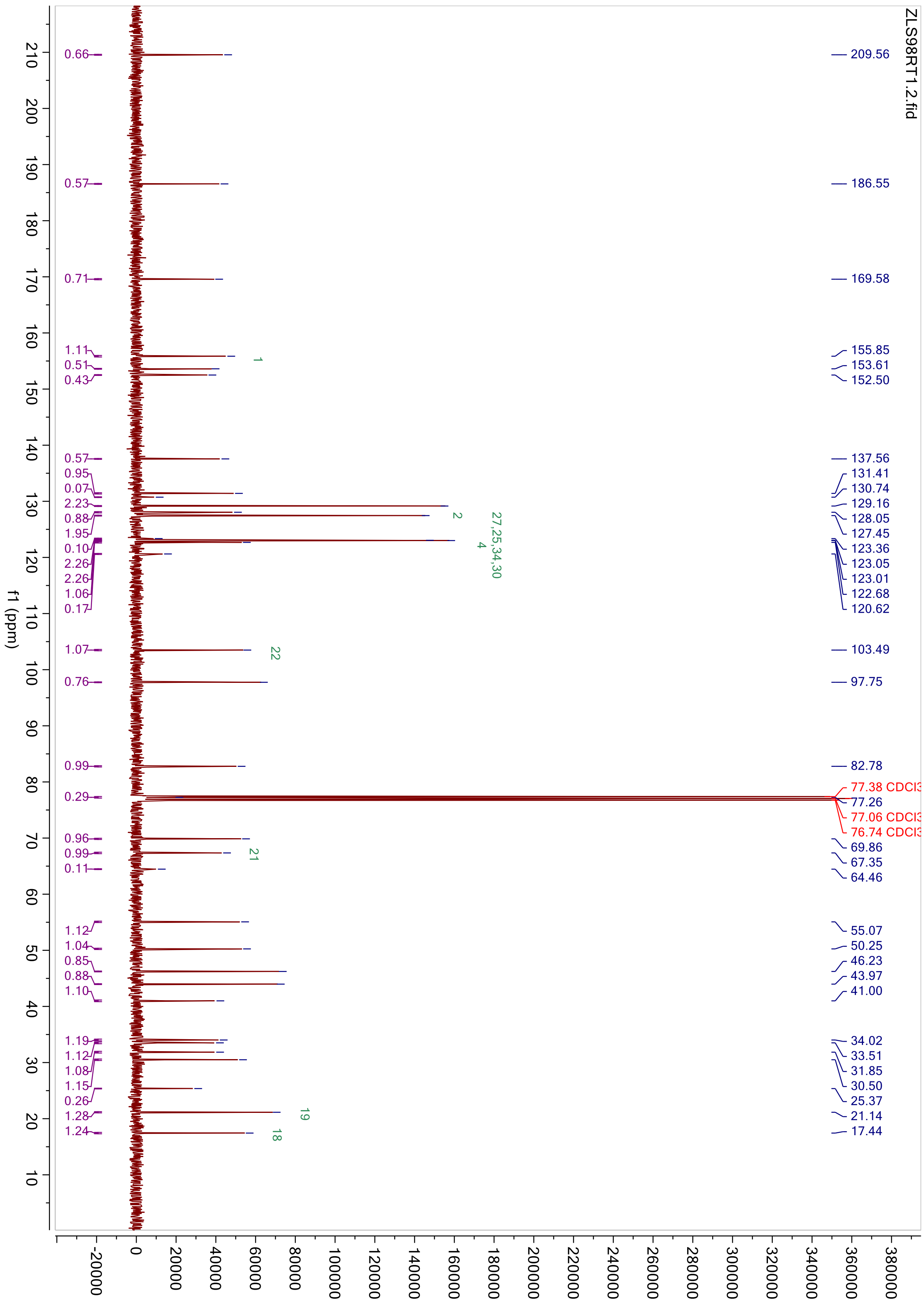

*S 5 HSQC NMR of AzoCort1(400MHz, CDCl$_3$)*

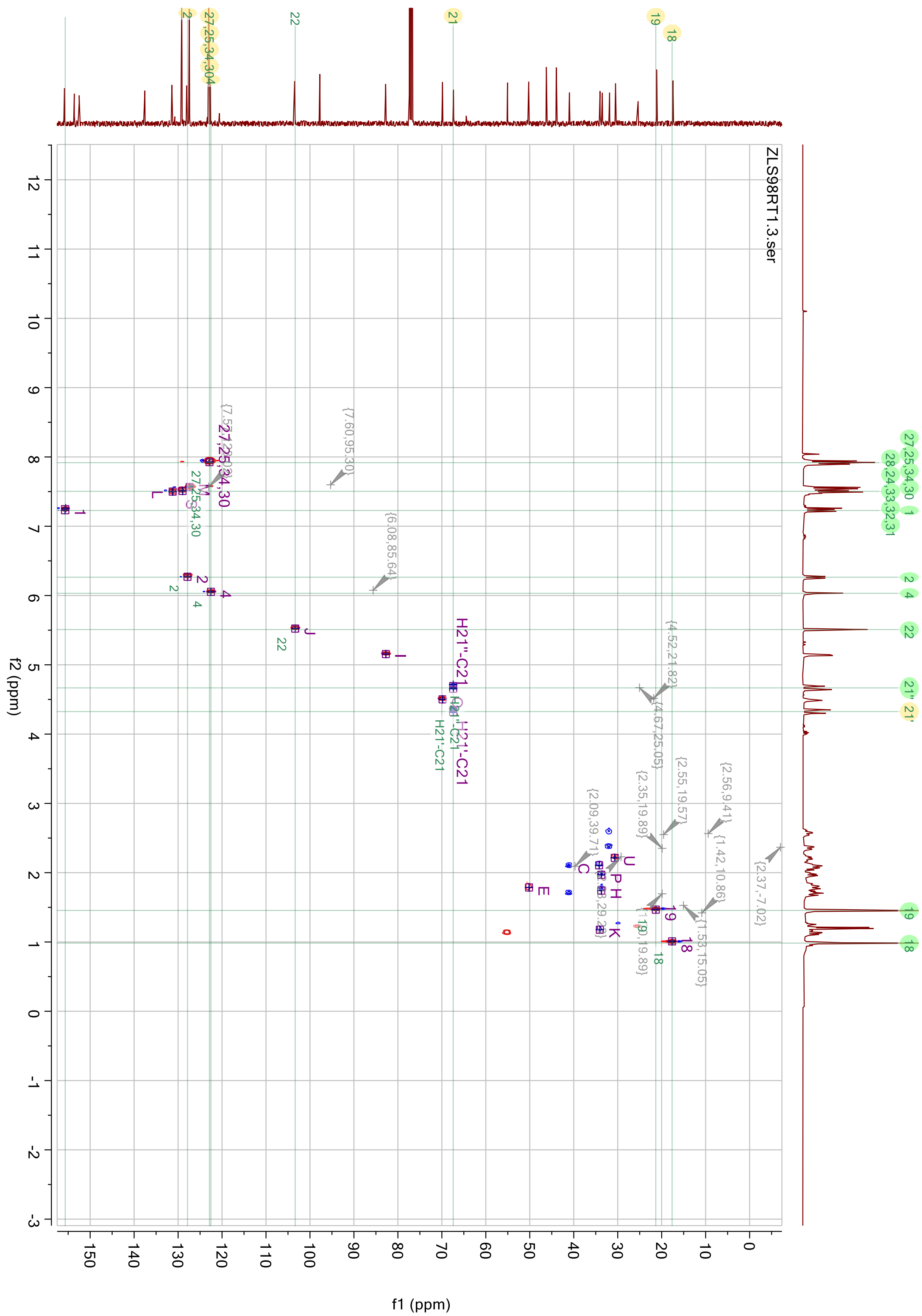

S 6 NOESY NMR of AzoCort1(400MHz, $CDCl_3$)

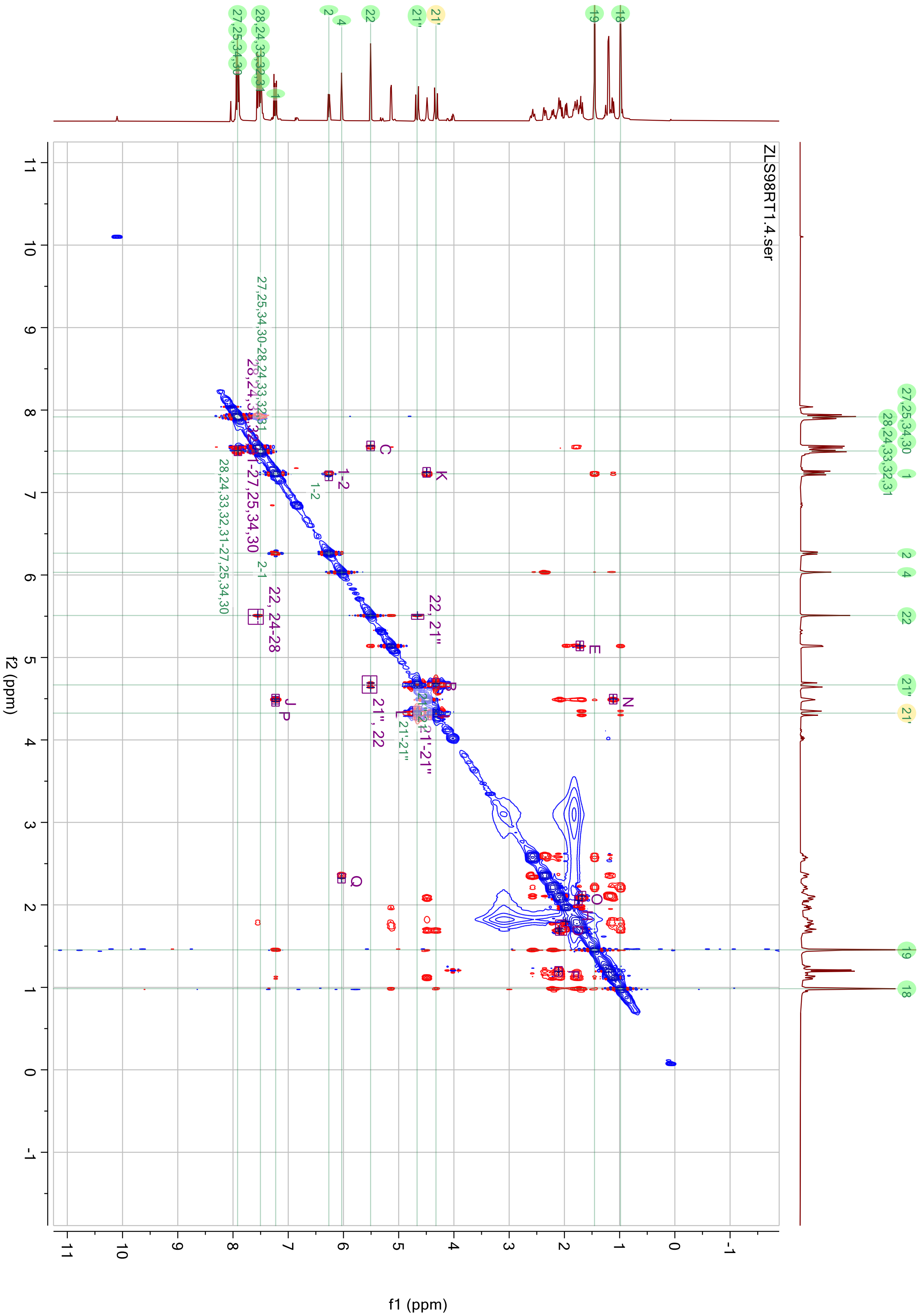

*S 7 ¹H NMR of AzoCort1β (400MHz, CDCl₃)*

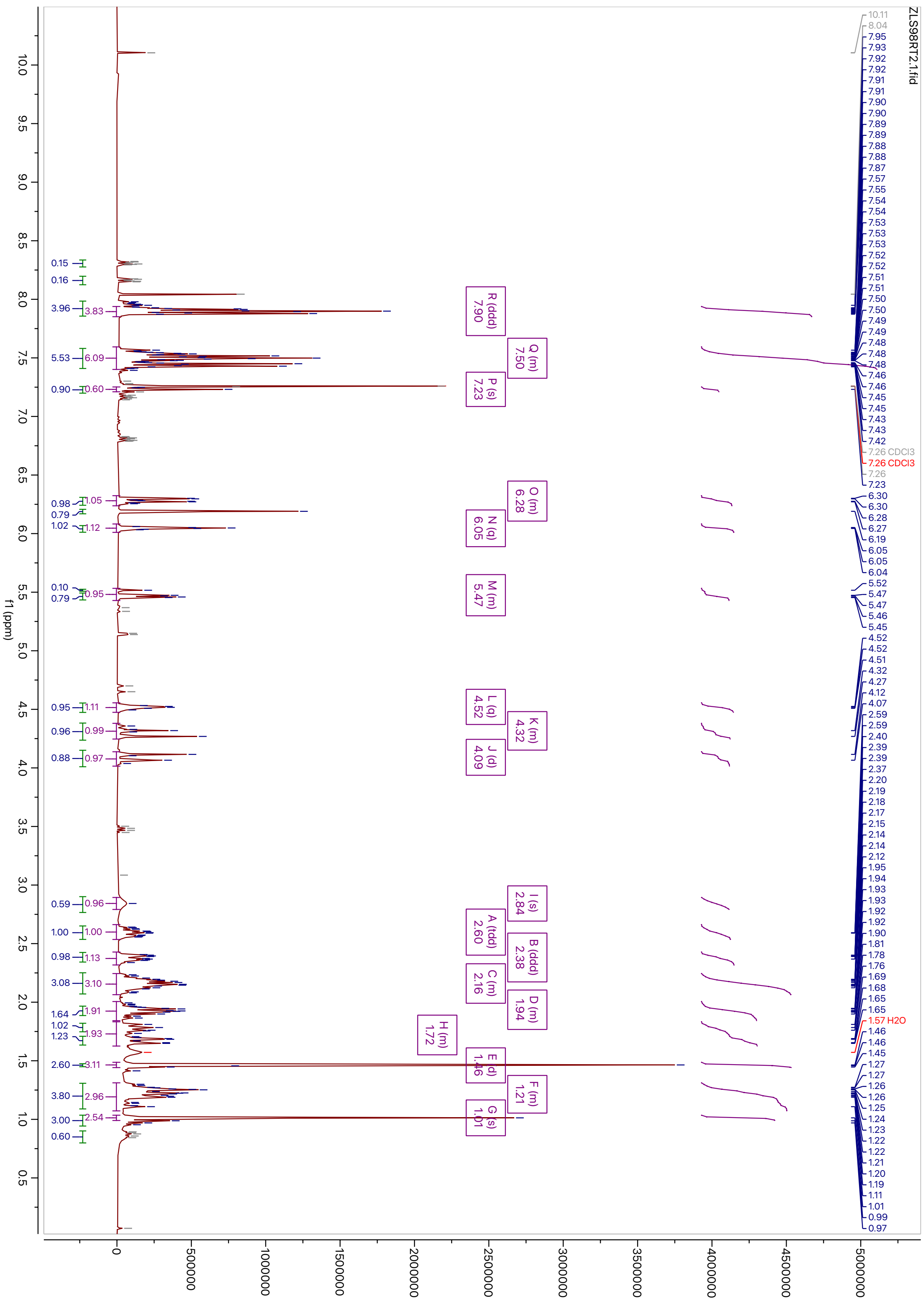

S 8 $^{13}C$ NMR of AzoCort1β (101MHz, $CDCl_3$)

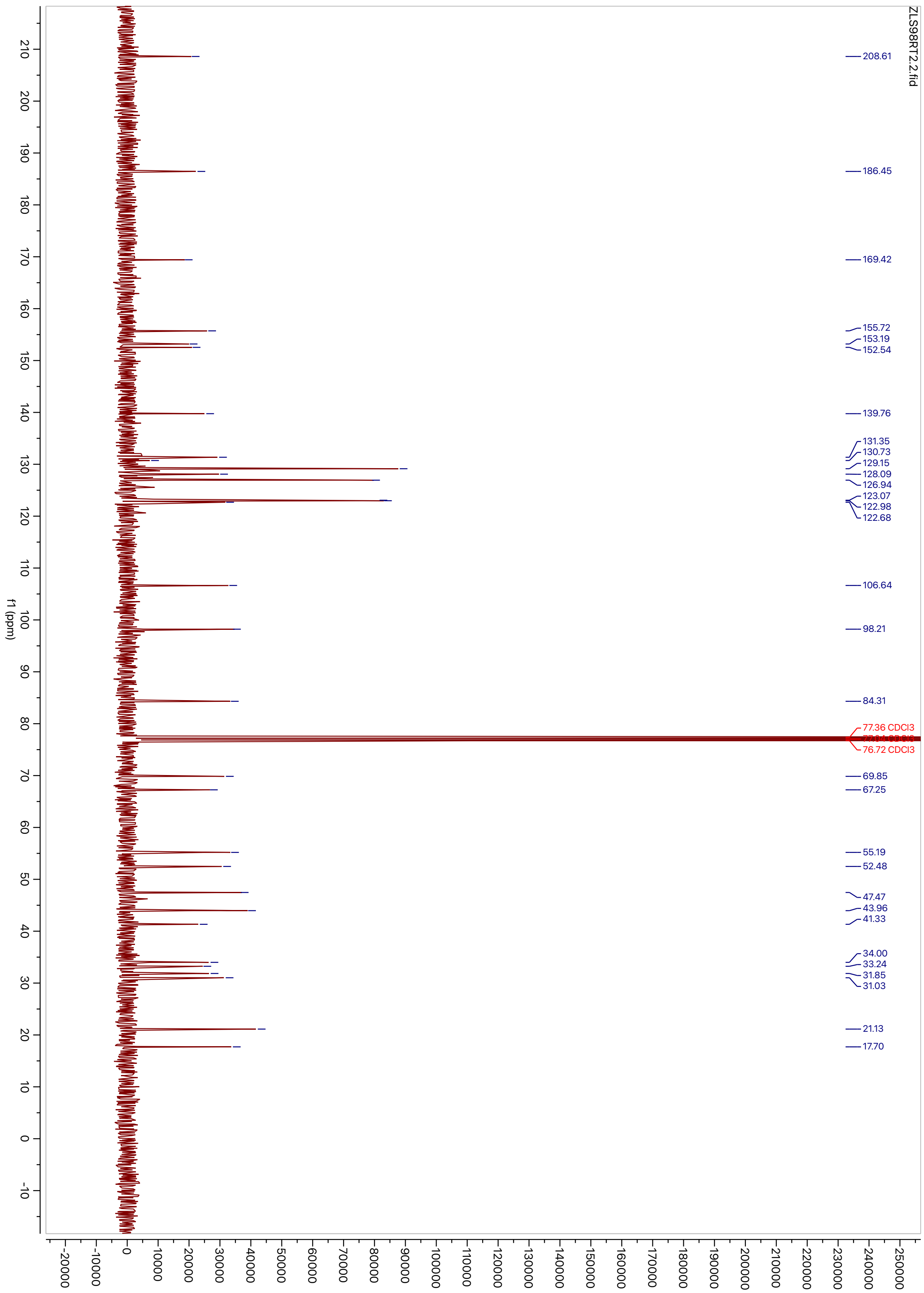

*S 9 HSQC 2D NMR of AzoCort1β (400MHz, $CDCl_3$)*

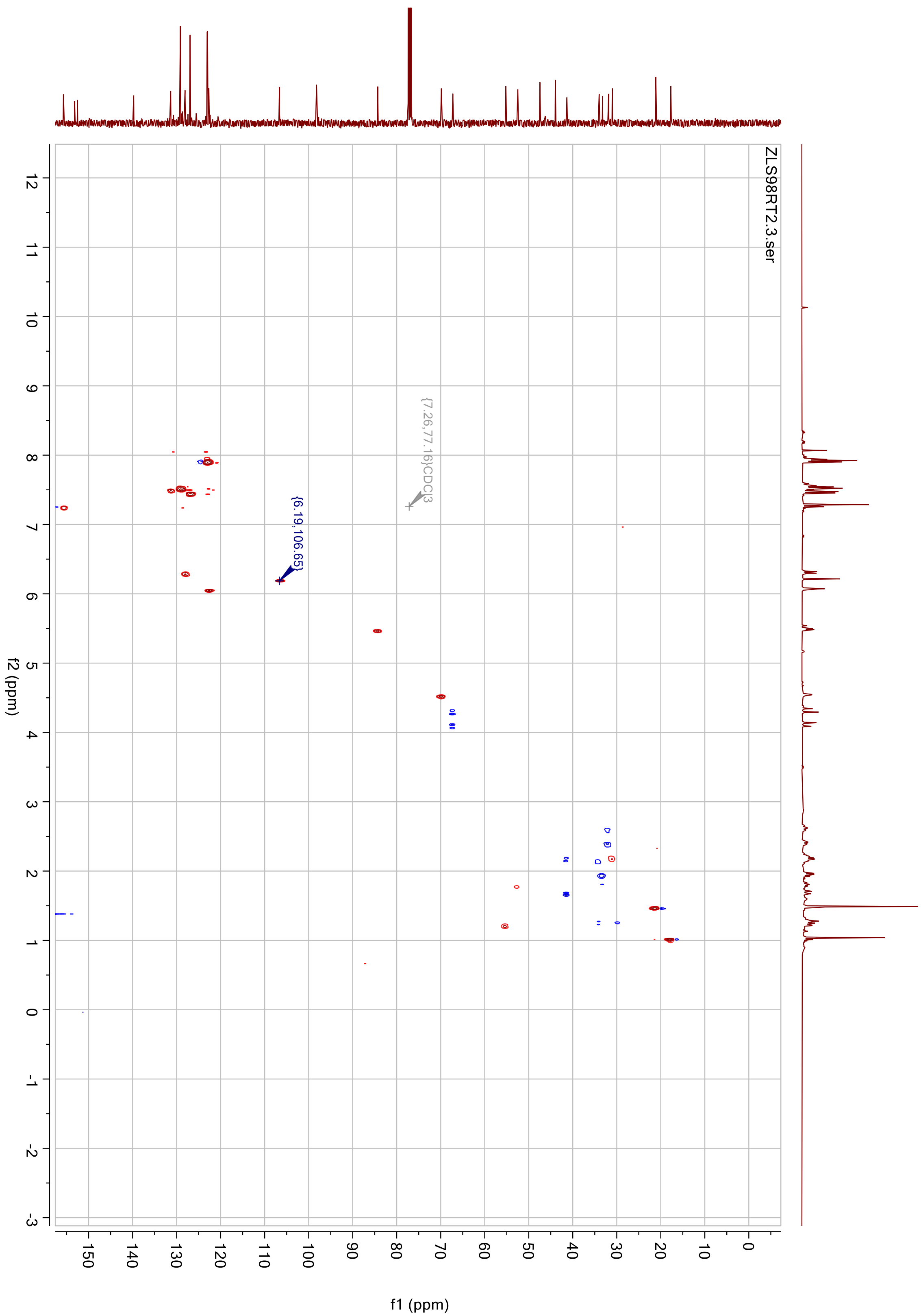

*S 10 NOESY 2D NMR of AzoCort1β (400MHz, $CDCl_3$)*

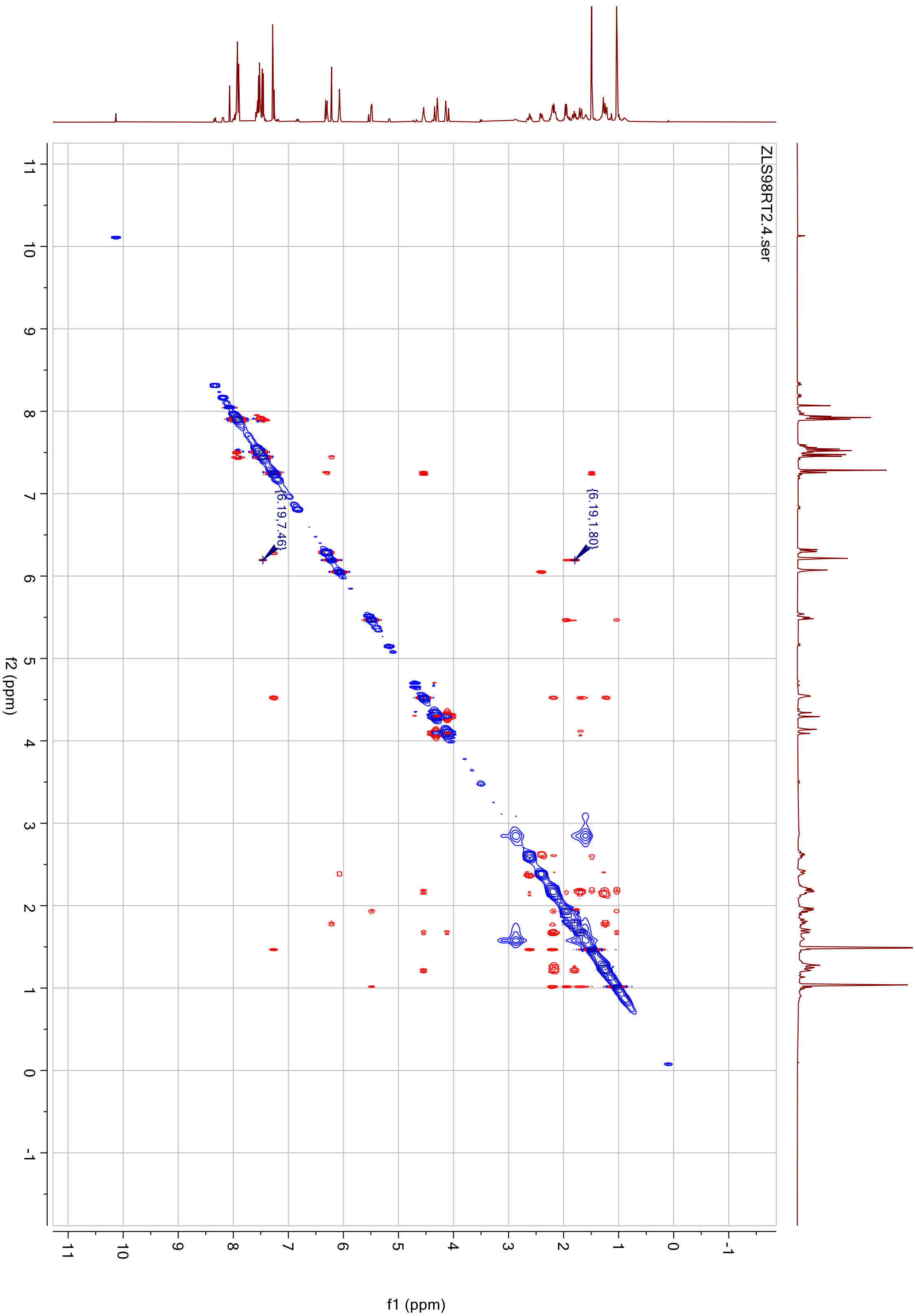

*S 11 $^{1}$H NMR of AzoCort2 (600MHz, MeOD)*

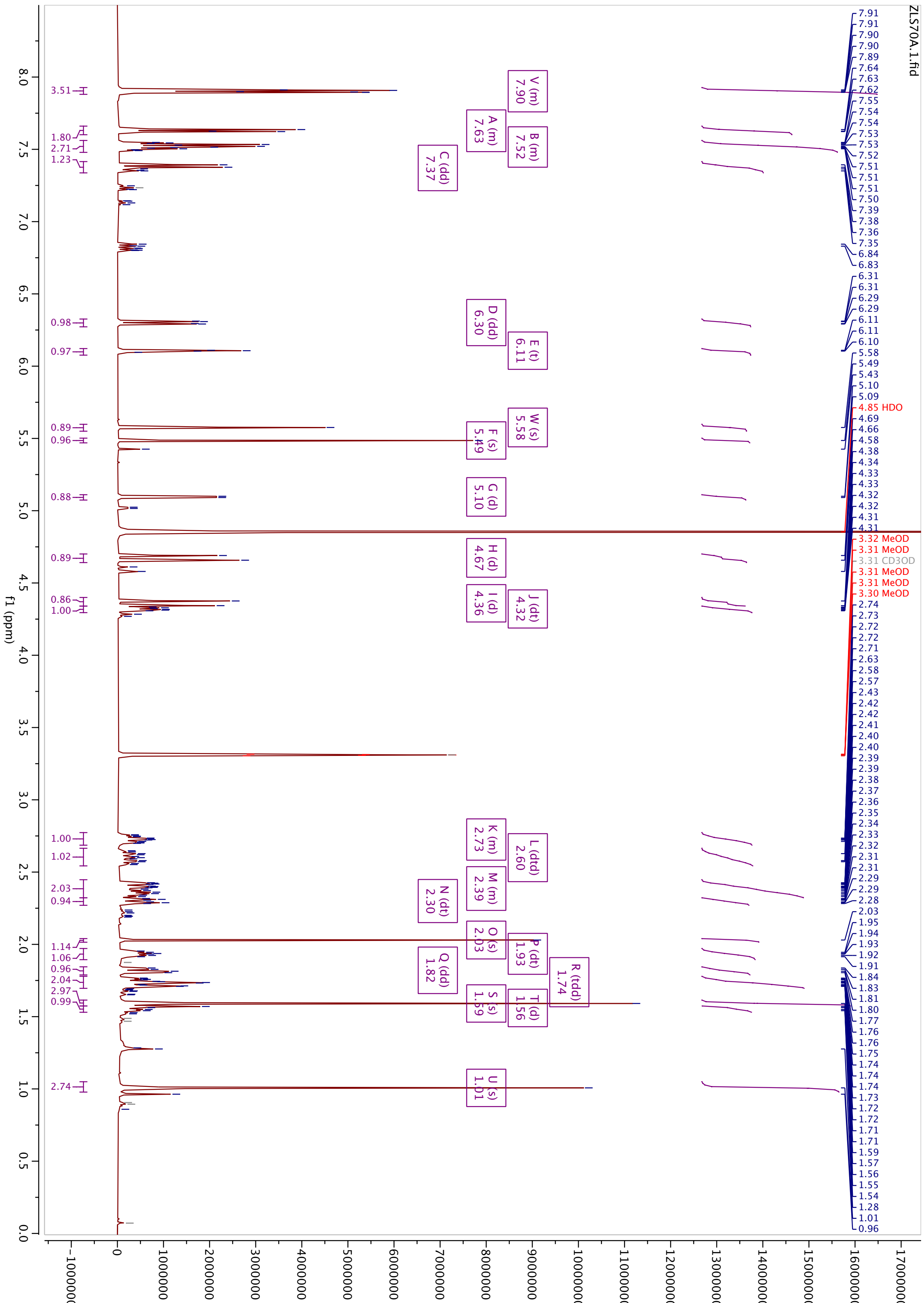

*S 12 $^{13}$C NMR of AzoCort2 (151MHz, MeOD)*

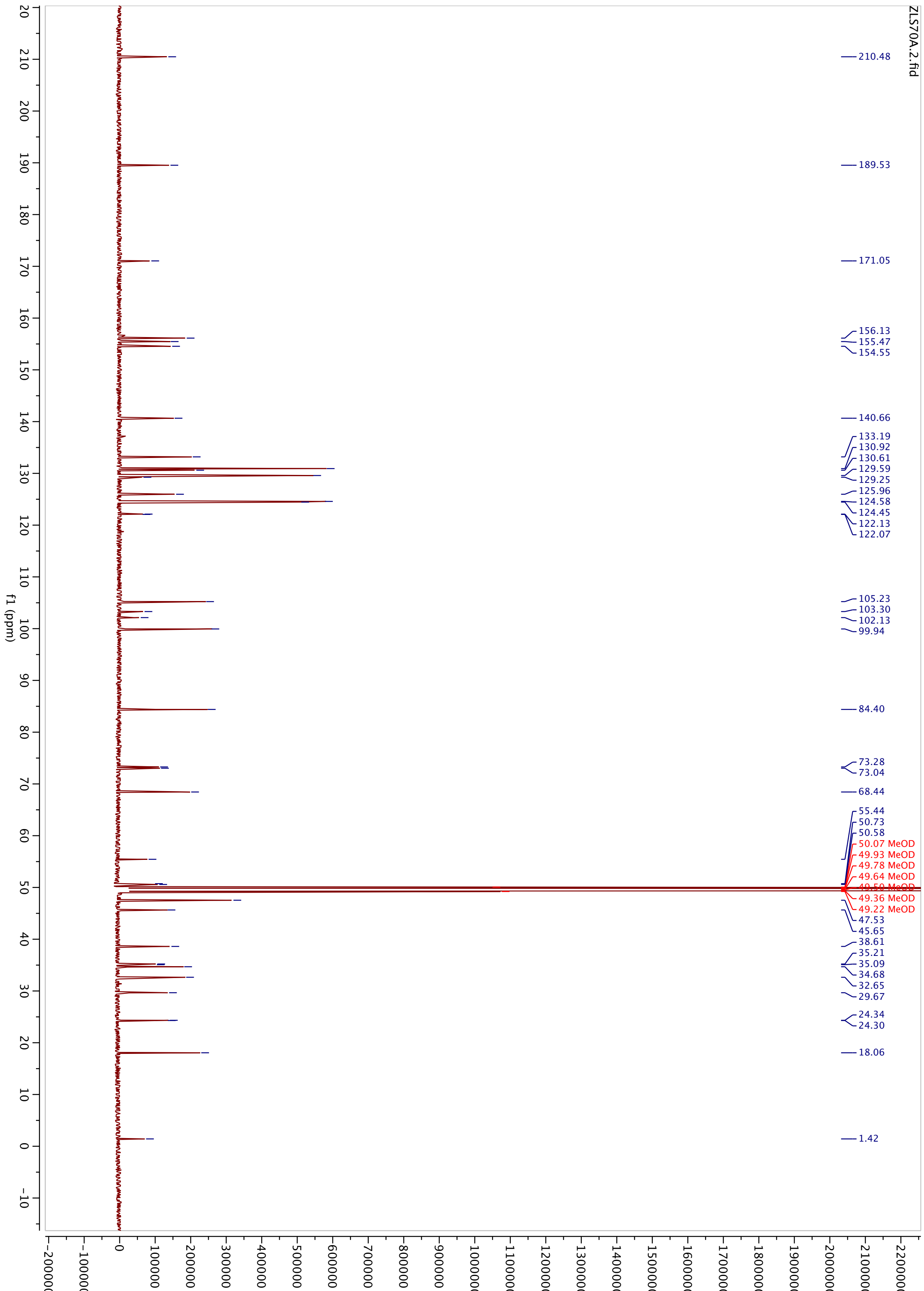

*S 13 HSQC 2D NMR of AzoCort2 (600MHz, MeOD)*

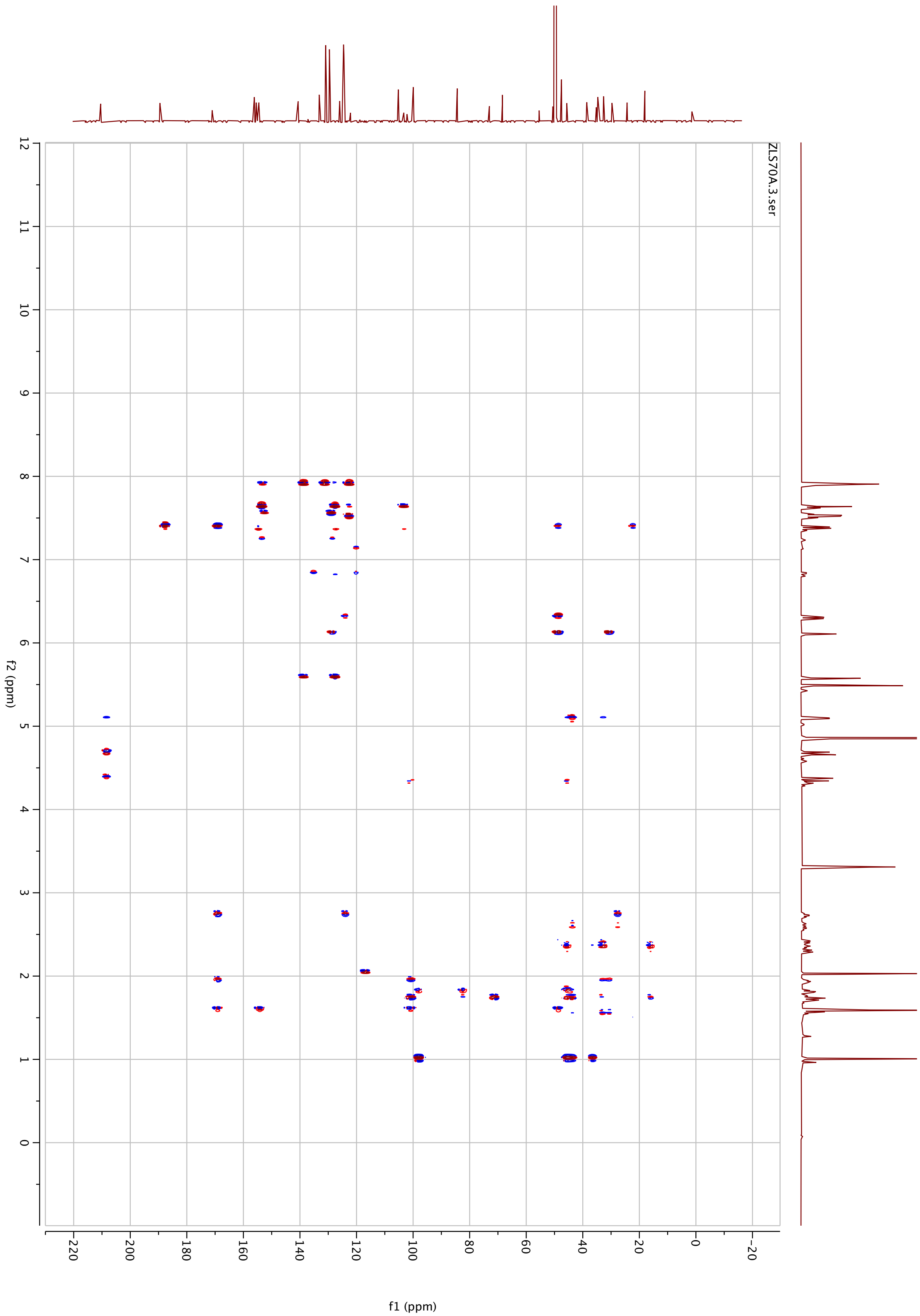

*S 14 NOESY 2D NMR of AzoCort2 (600MHz, MeOD)*

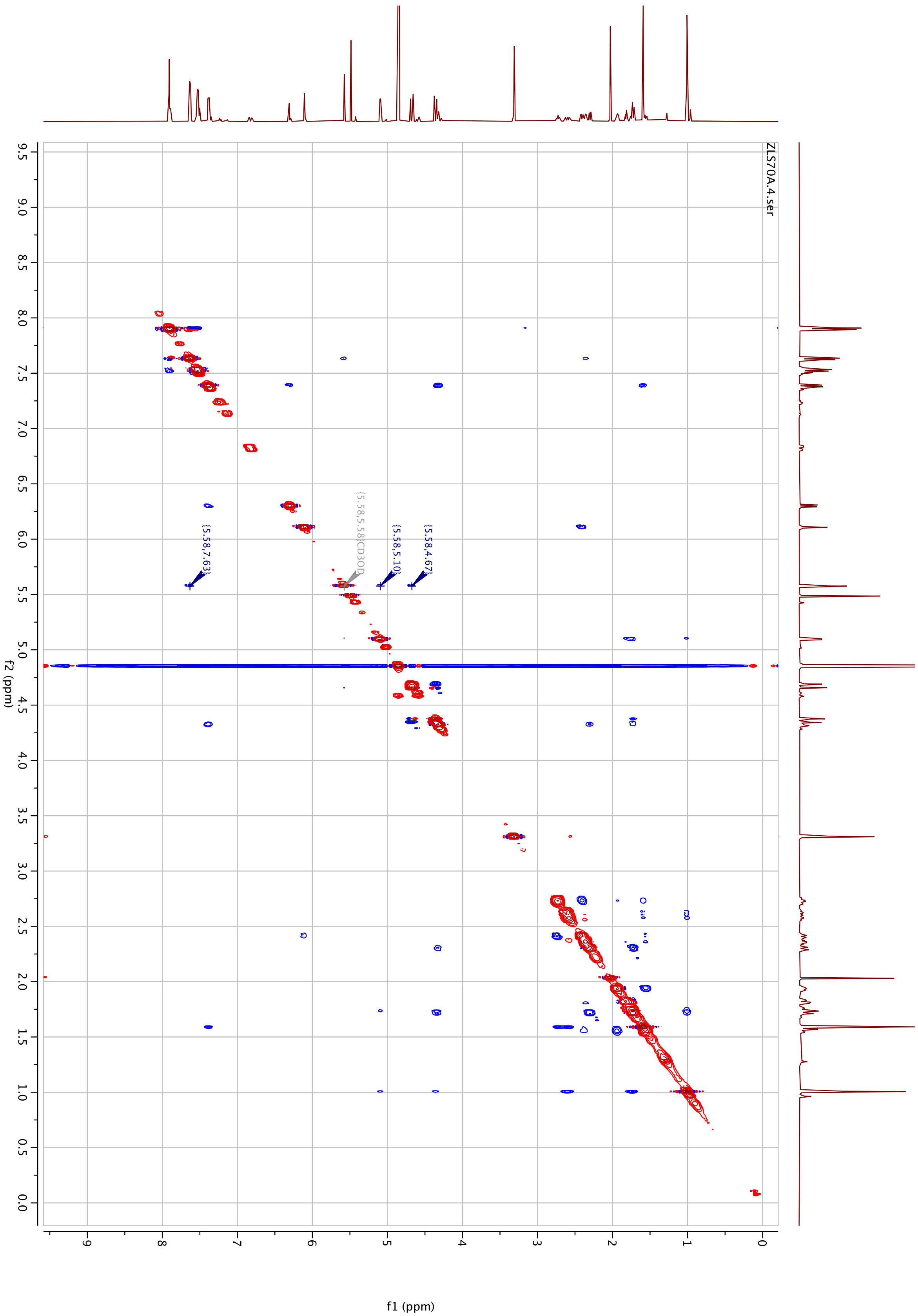

*S 15 ¹H NMR of AzoCort2β (600MHz, MeOD)*

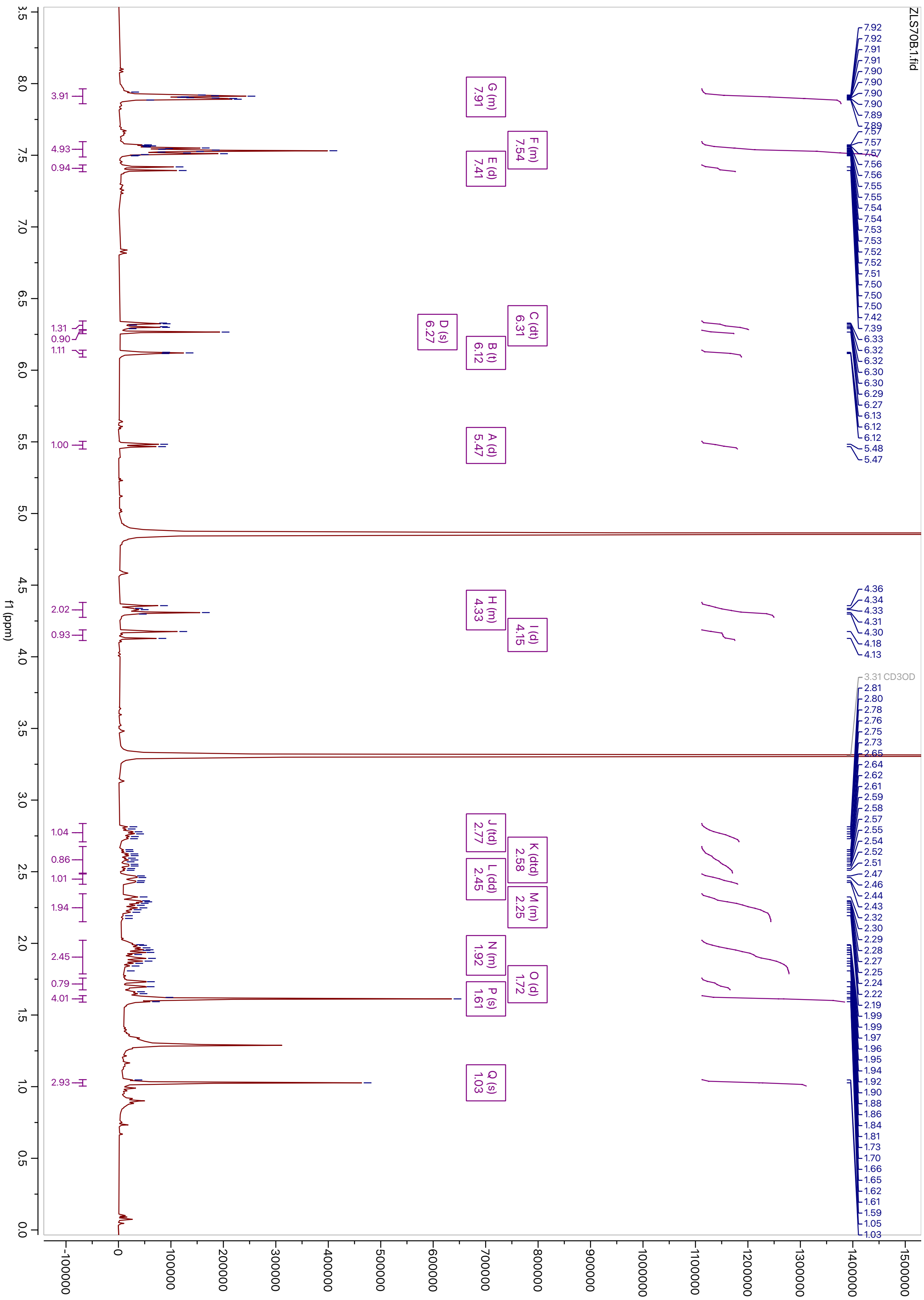

S 16 13C NMR of AzoCort2$\beta$ (151MHz, MeOD)

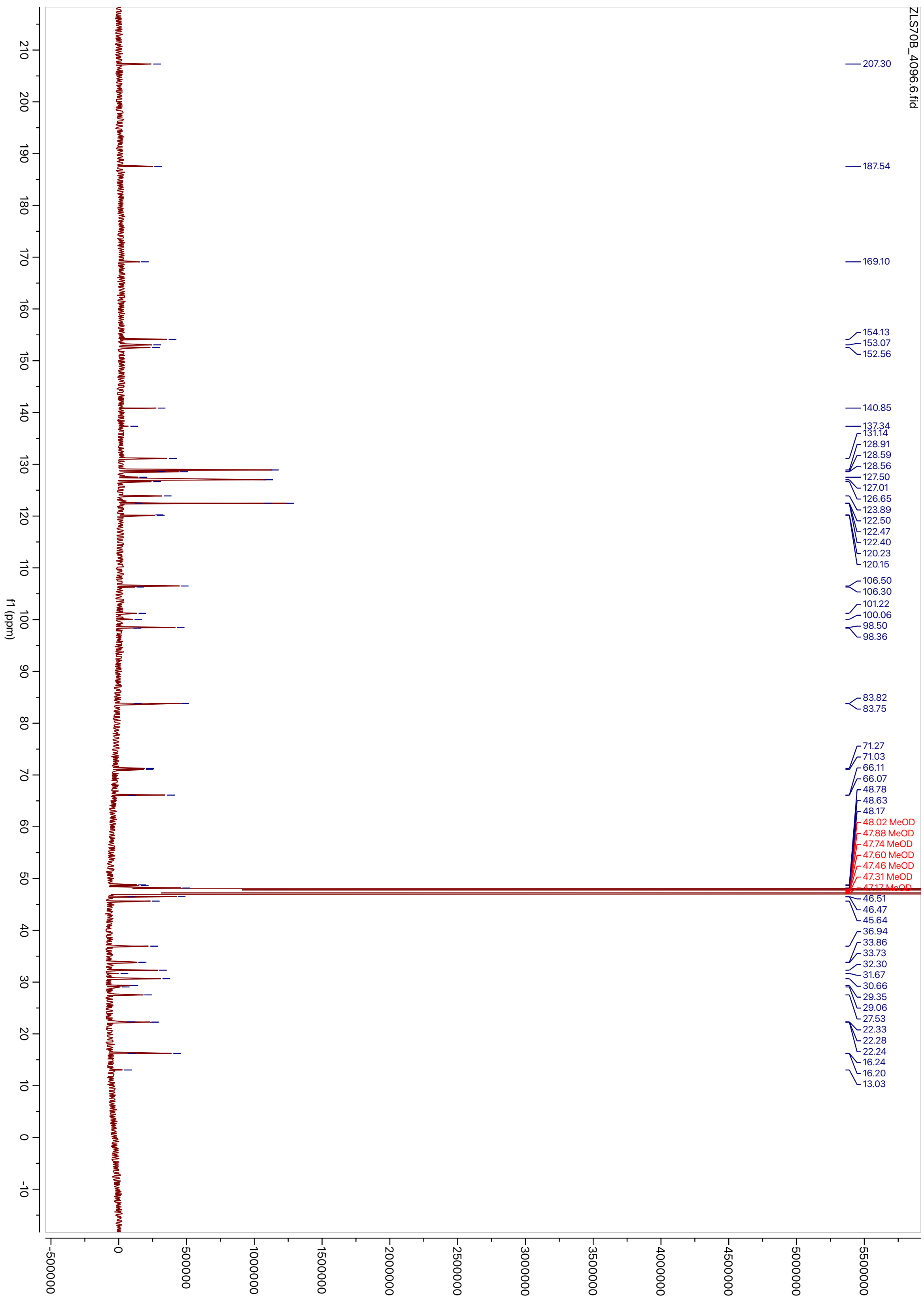

*S 17 NOESY 2D NMR of AzoCort2β (600MHz, MeOD)*

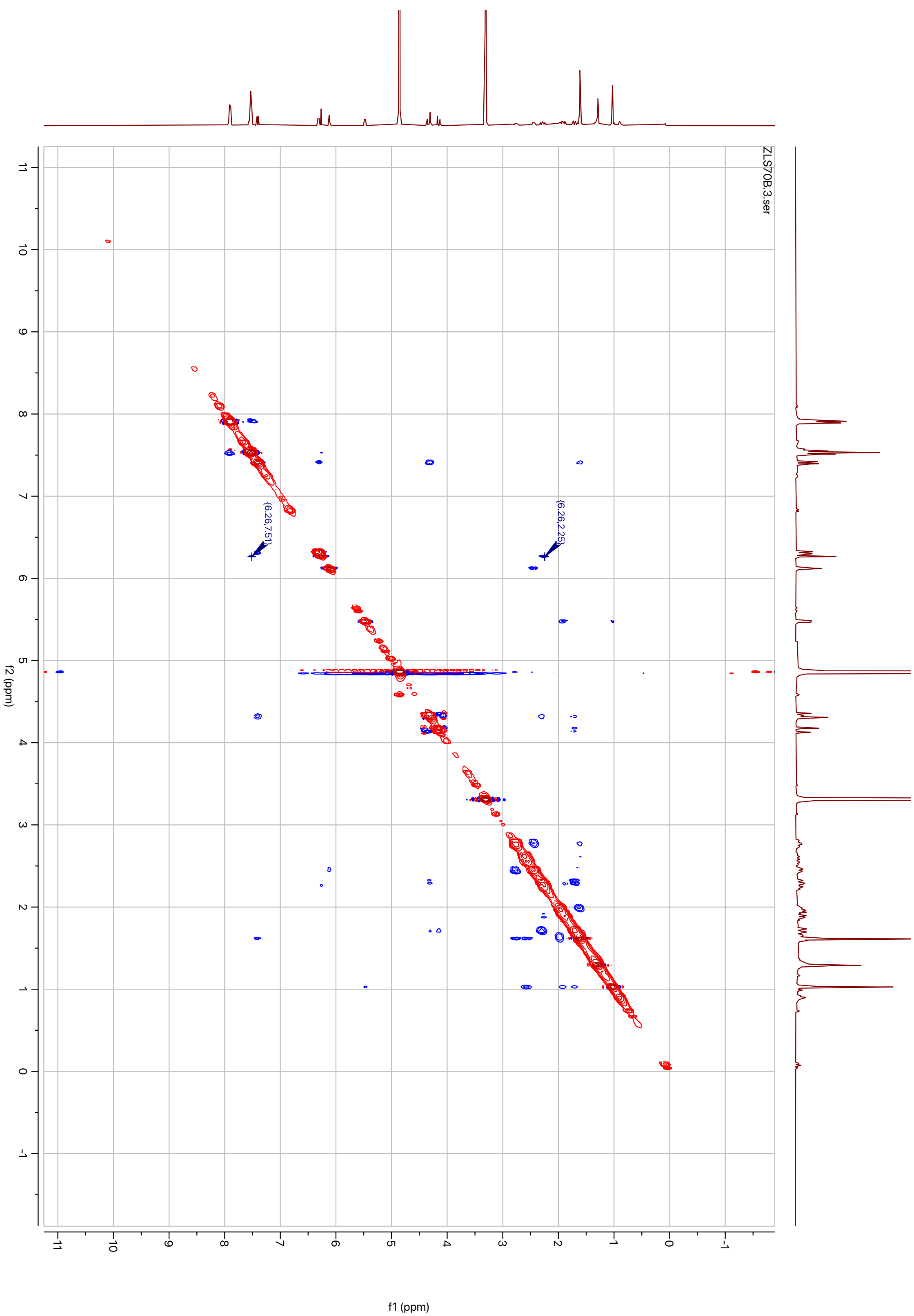

*S 18 ¹H NMR of AzoCort3 (600MHz, MeOD)*

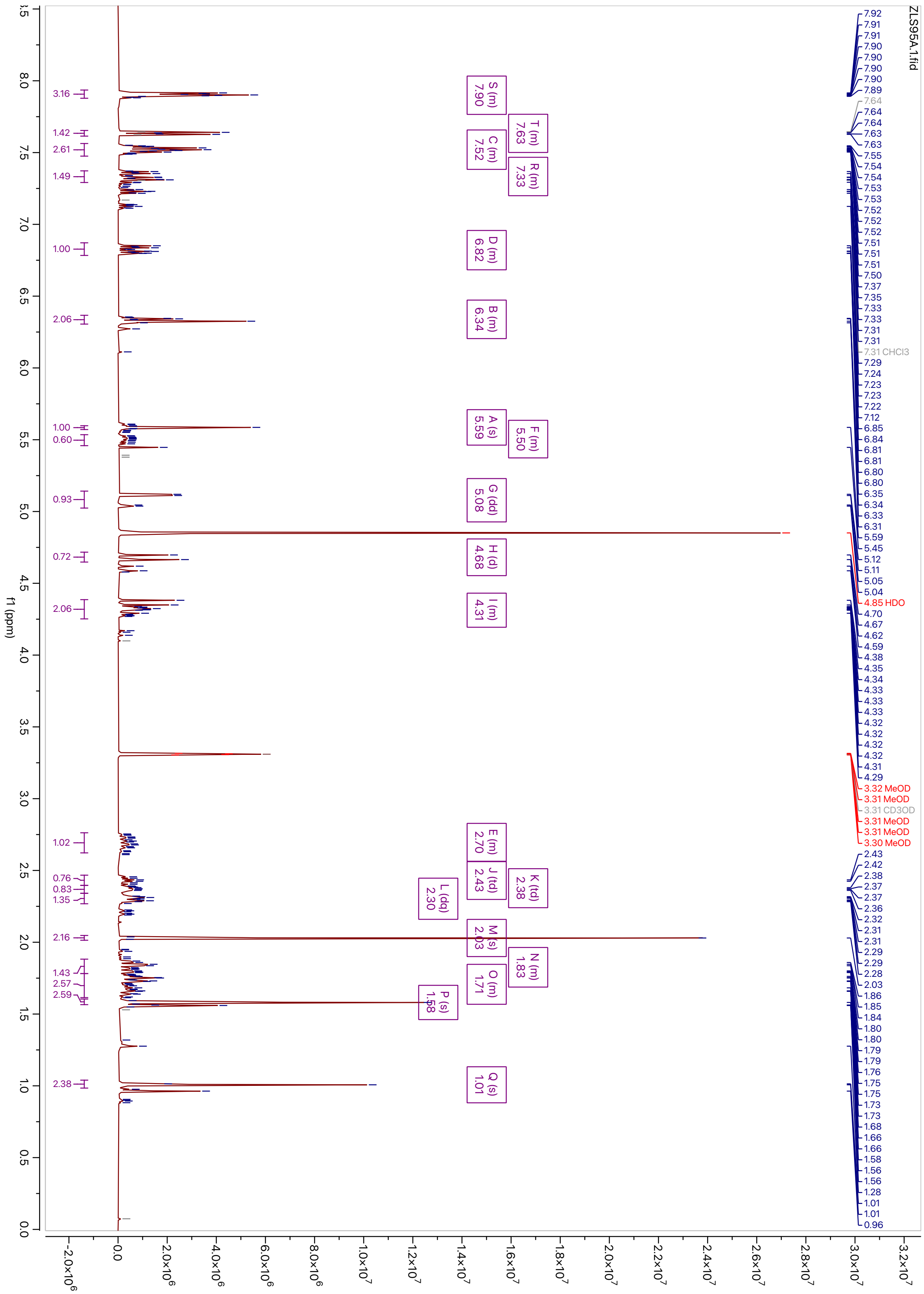

*S 19 $^{13}$C NMR of AzoCort3 (151MHz, MeOD)*

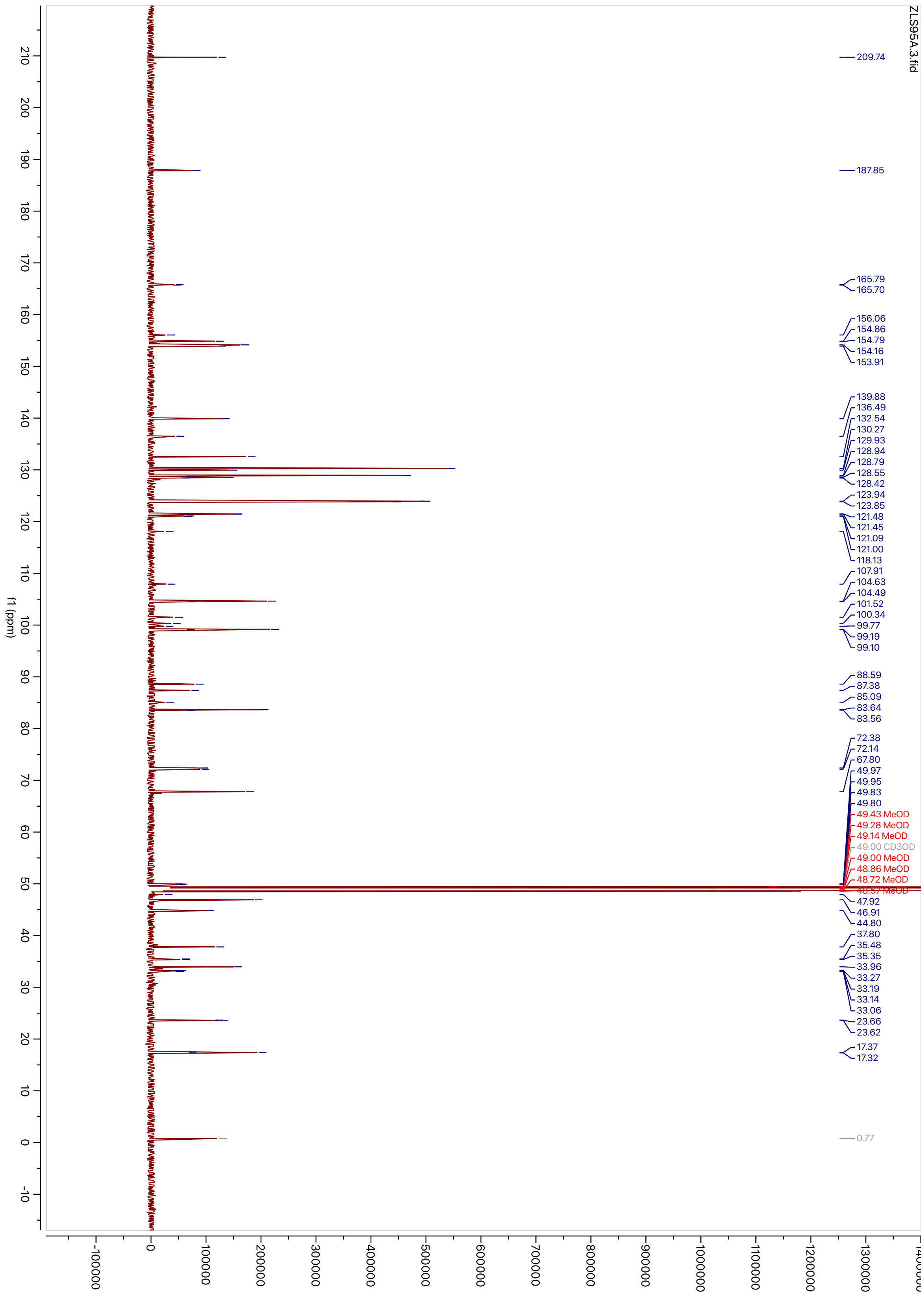

*S 20 HSQC NMR of AzoCort3 (600MHz, MeOD)*

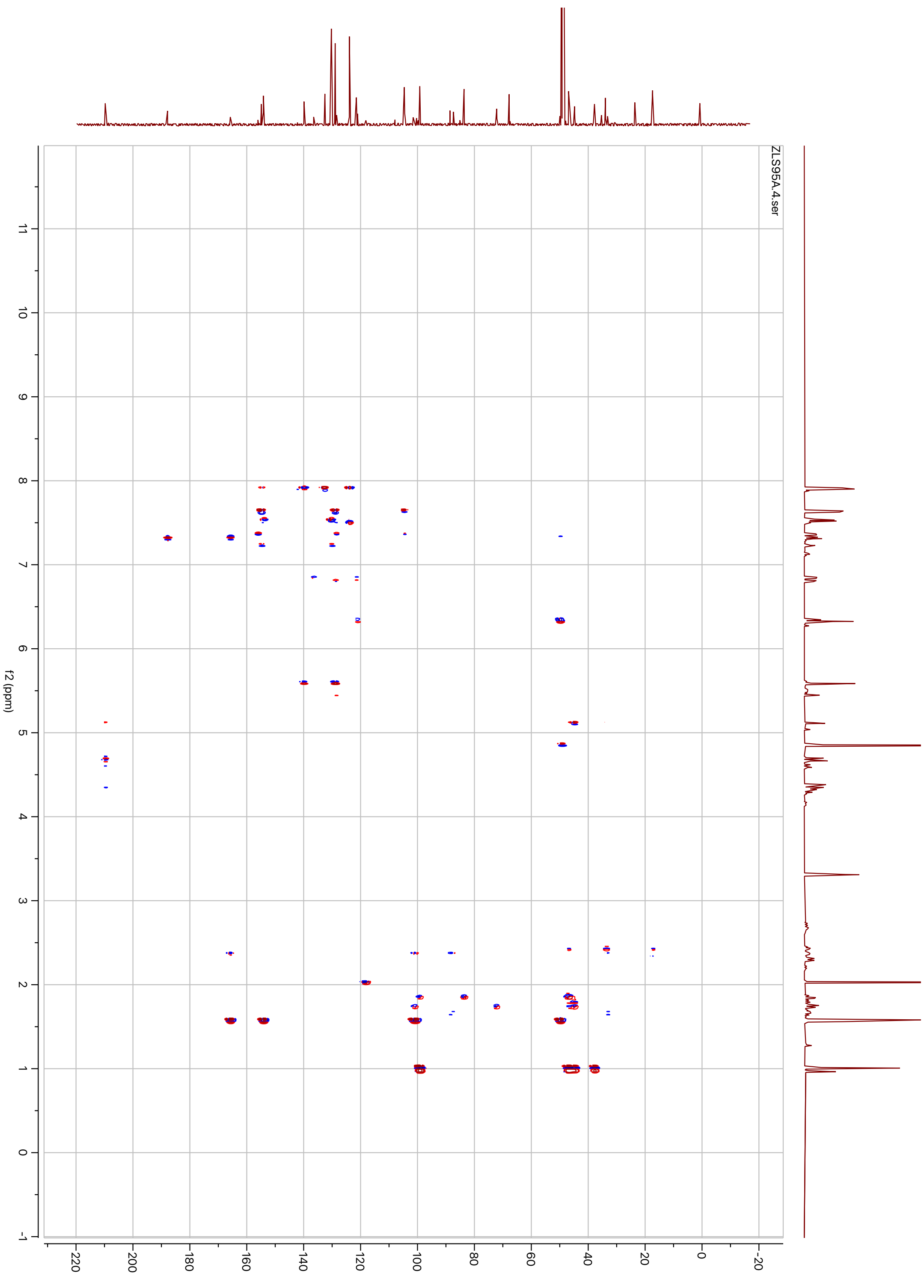

*S 21 NOESY NMR of AzoCort3 (600MHz, MeOD)*

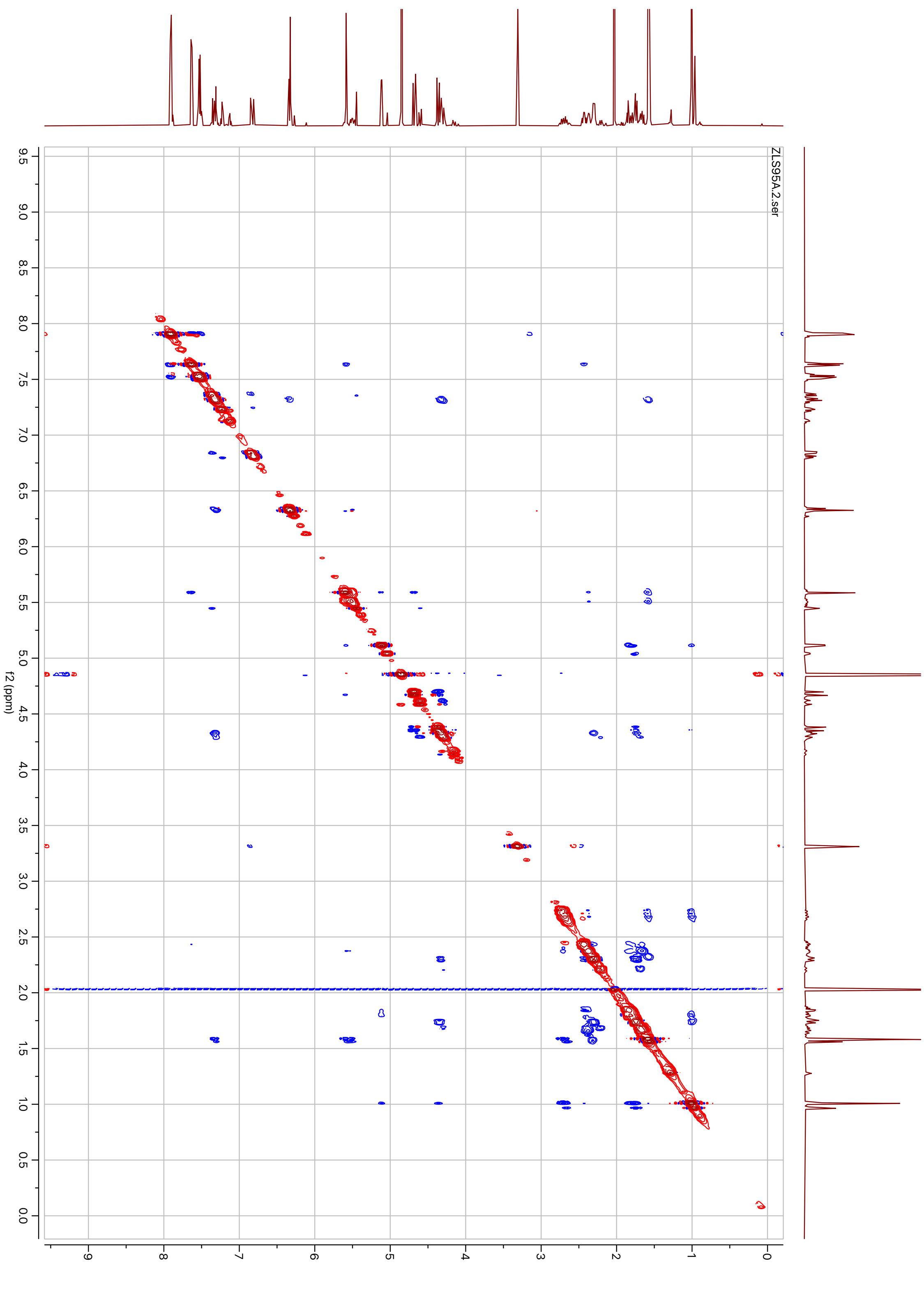

*S 22 ¹H NMR of AzoCort3β (400MHz, MeOD)*

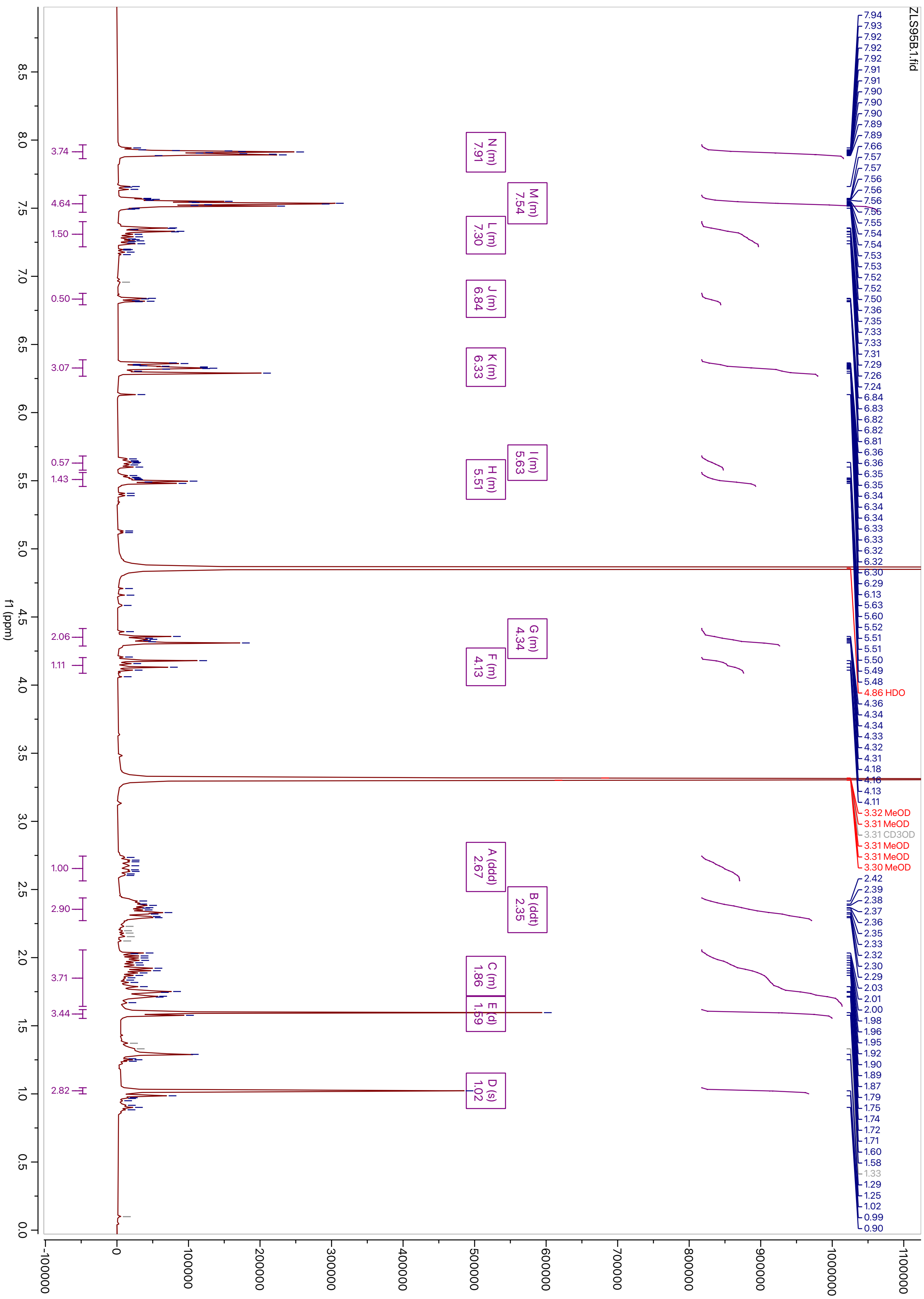

*S 23 $^{13}$C NMR of AzoCort3β (151MHz, MeOD)*

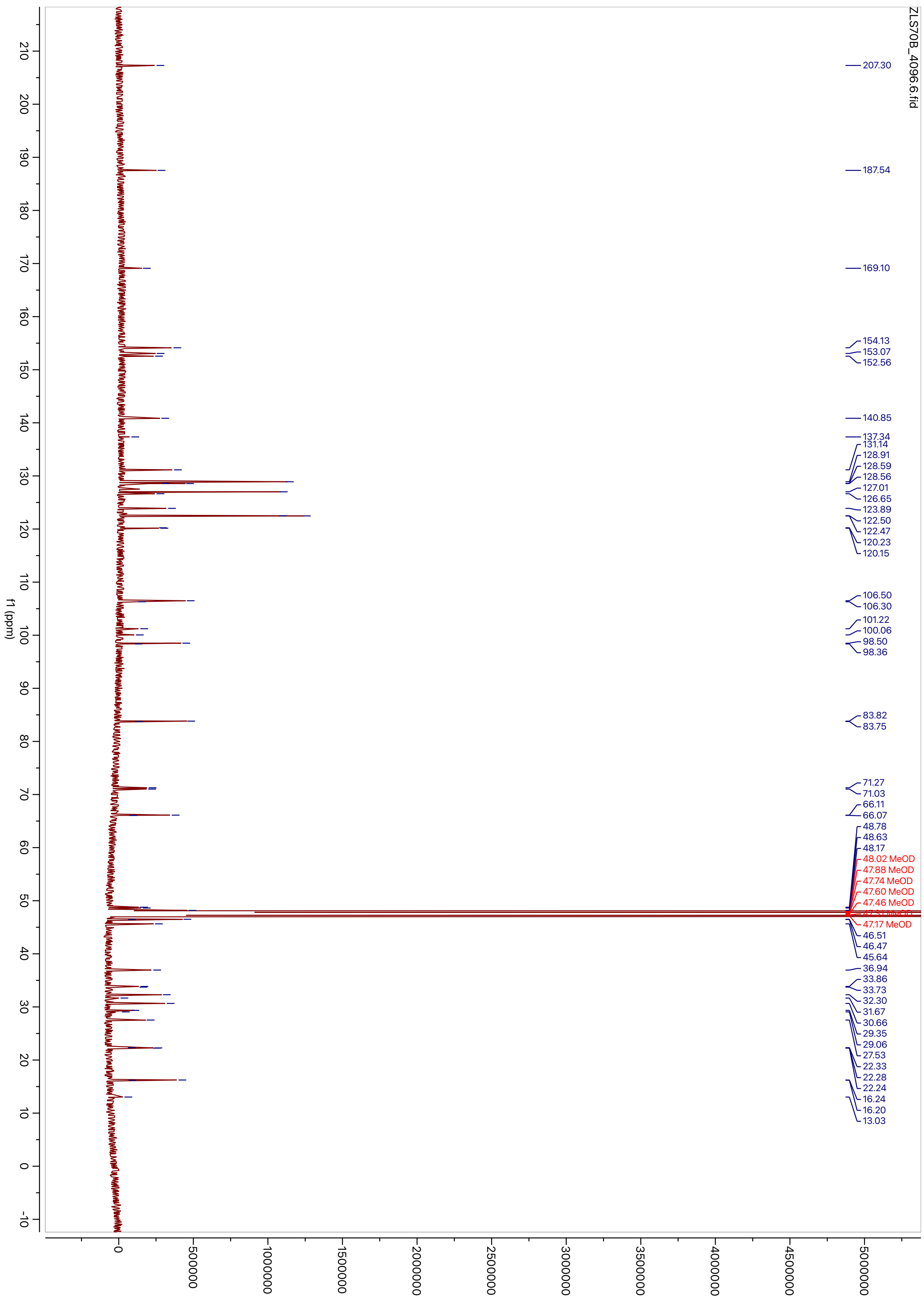

*S 24 NOESY NMR of AzoCort3β (400MHz, MeOD)*

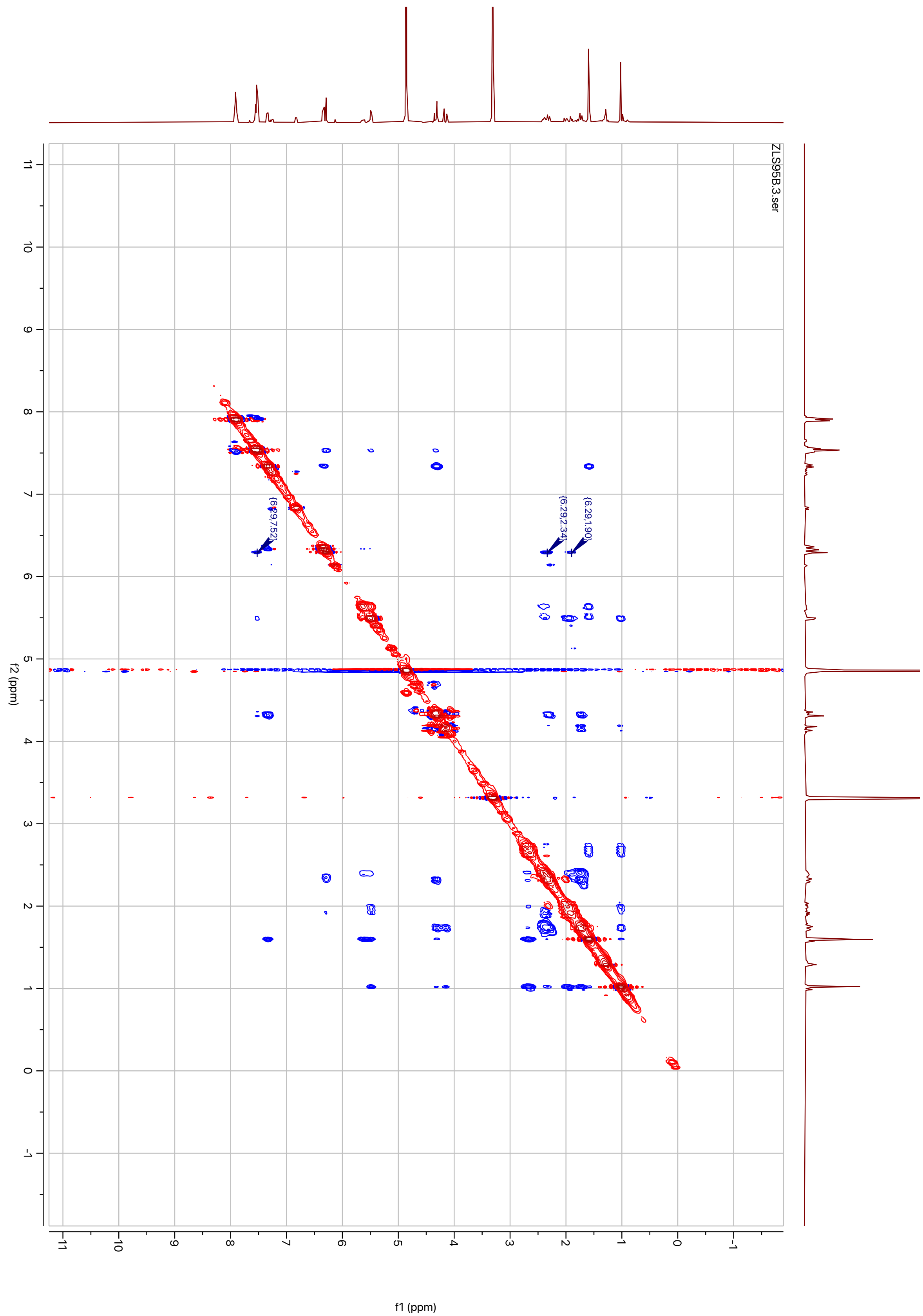

S 25 $^{1}$H NMR of AzoCort4 (400MHz, MeOD)

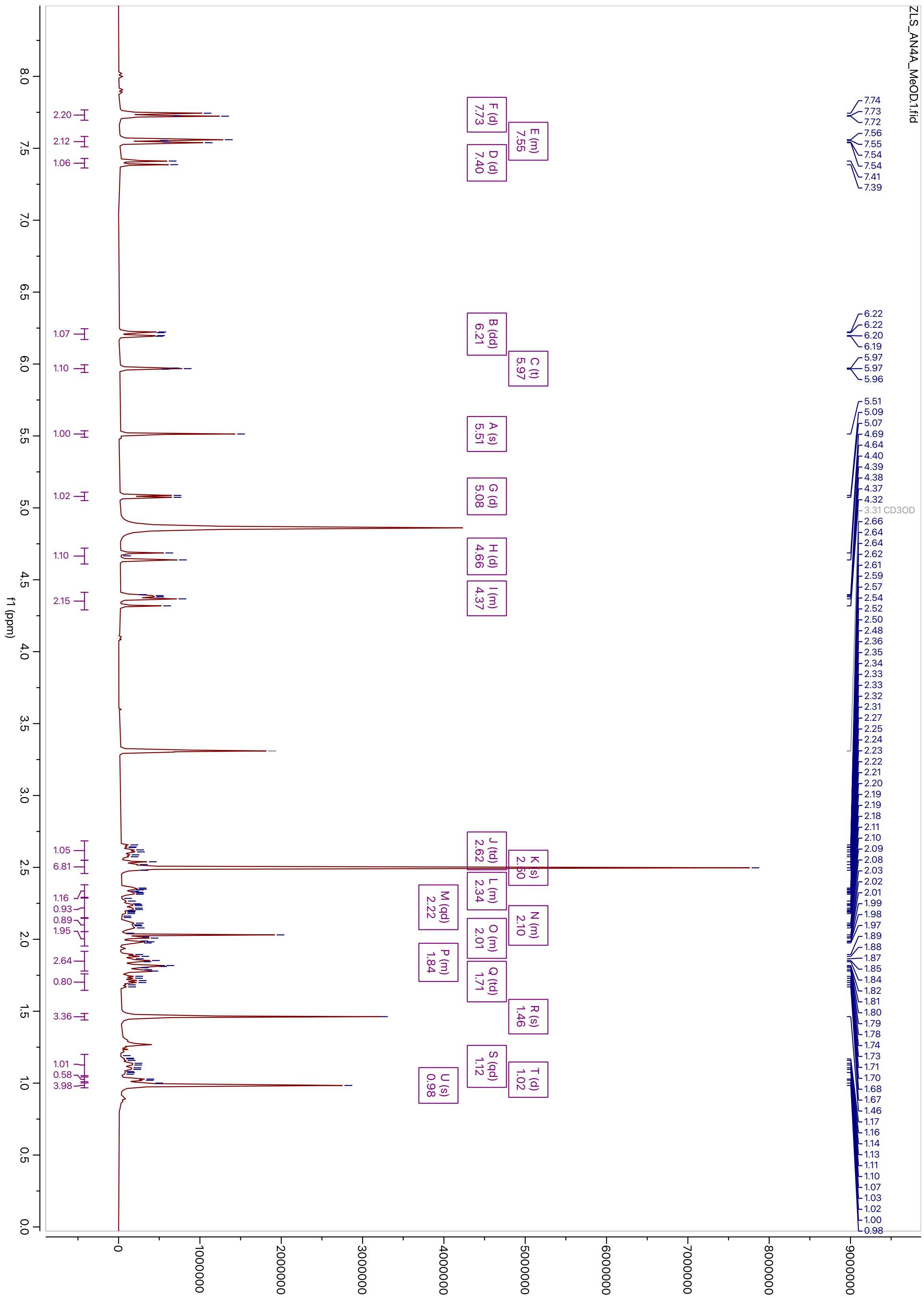

*S 26 13C NMR of AzoCort4 (101MHz, MeOD)*

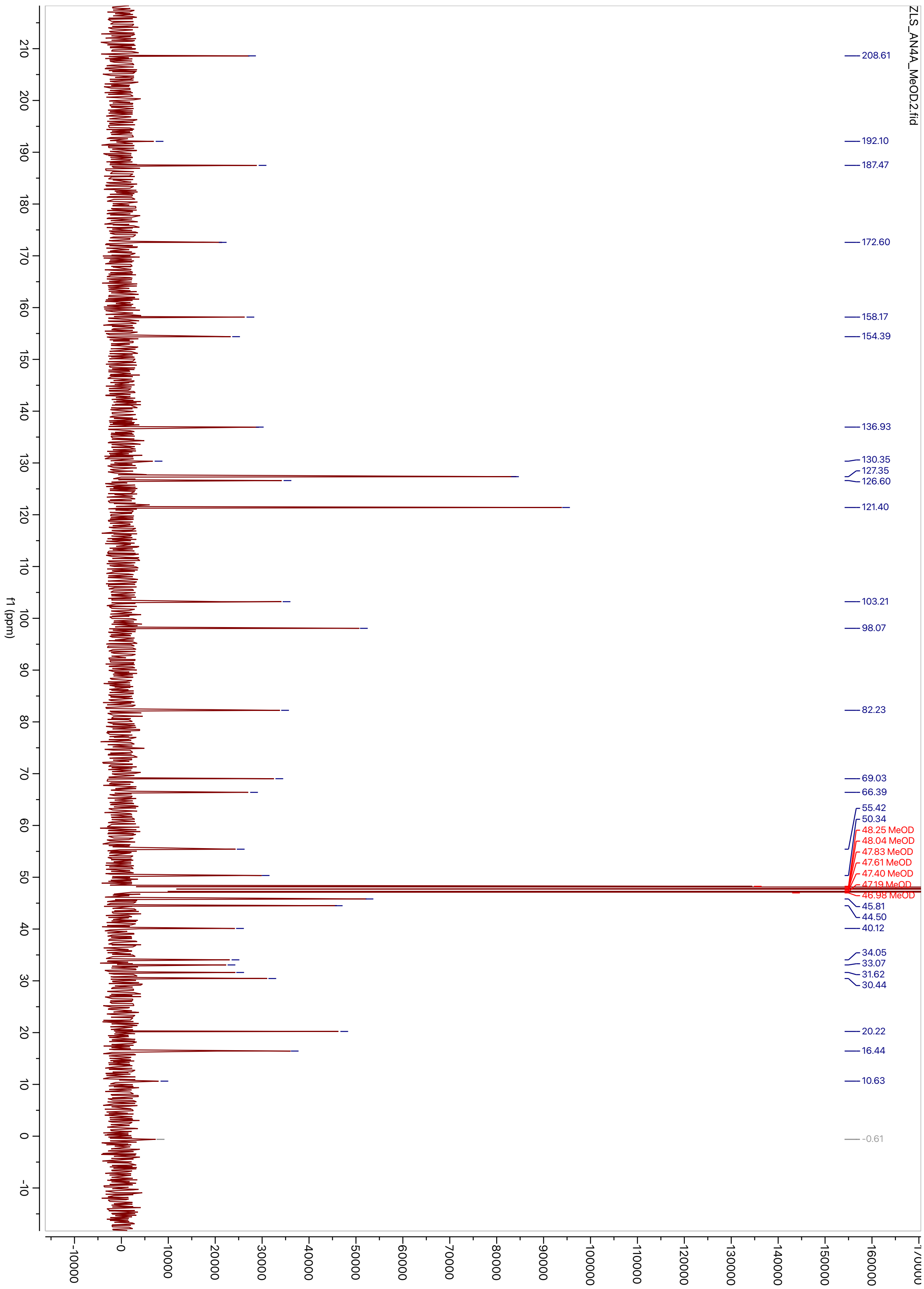

*S 27 HSQC NMR of AzoCort4 (400MHz, MeOD)*

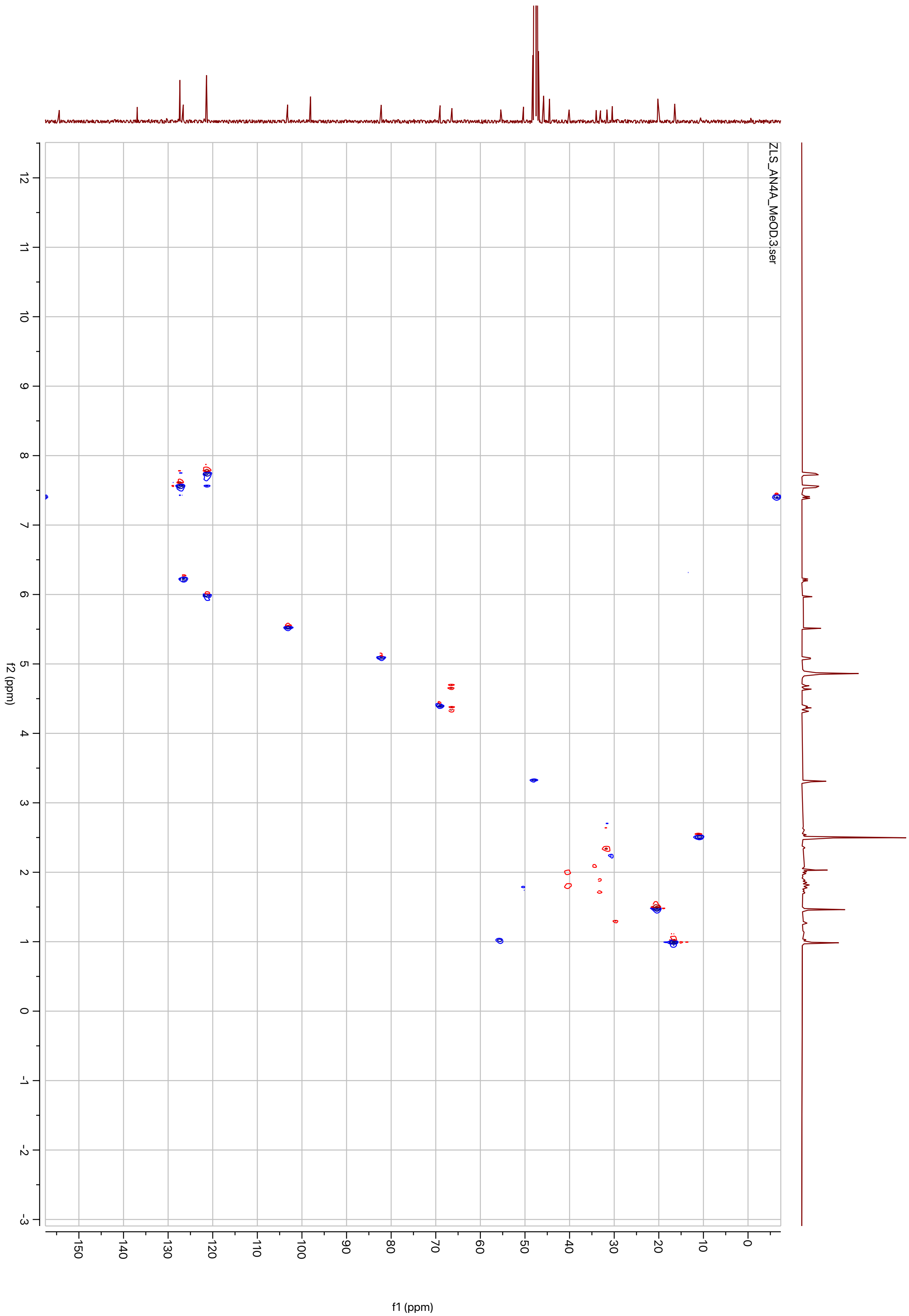

S 28 NOESY NMR of AzoCort4 (400MHz, MeOD)

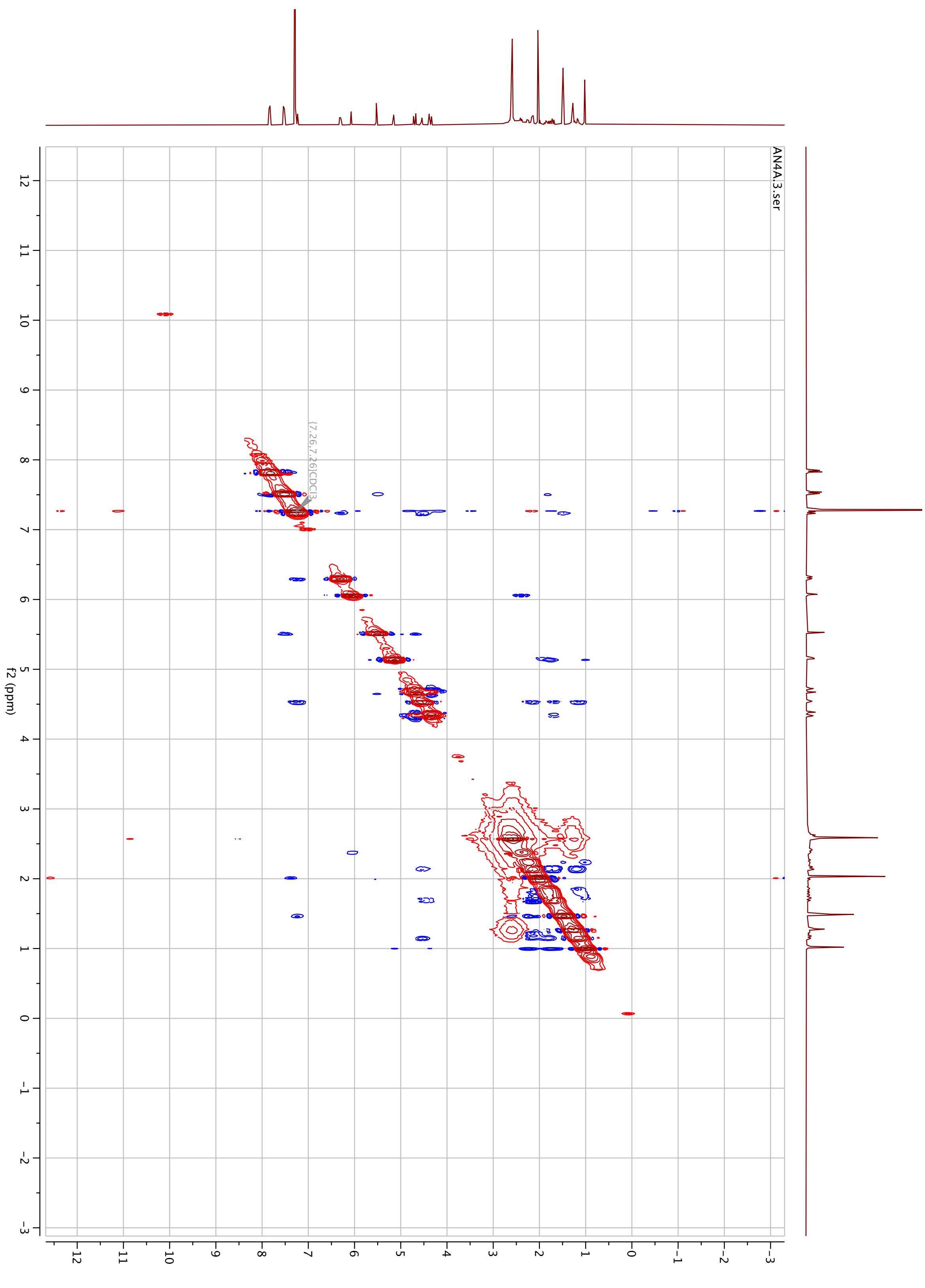

*S 29 1H NMR of AzoCort4β (400MHz, MeOD)*

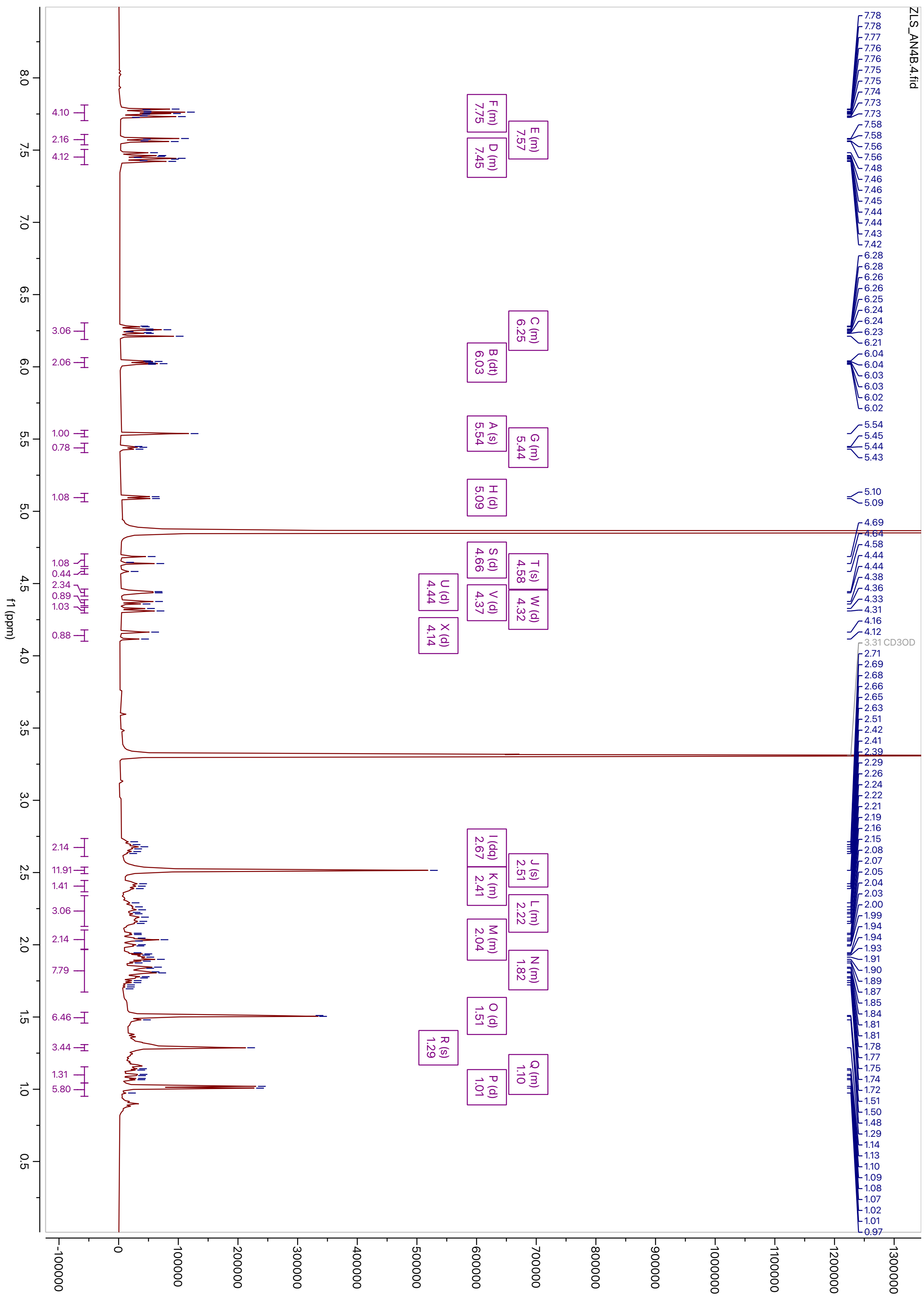

S 30 13C NMR of AzoCort4$\beta$ (151MHz, MeOD)

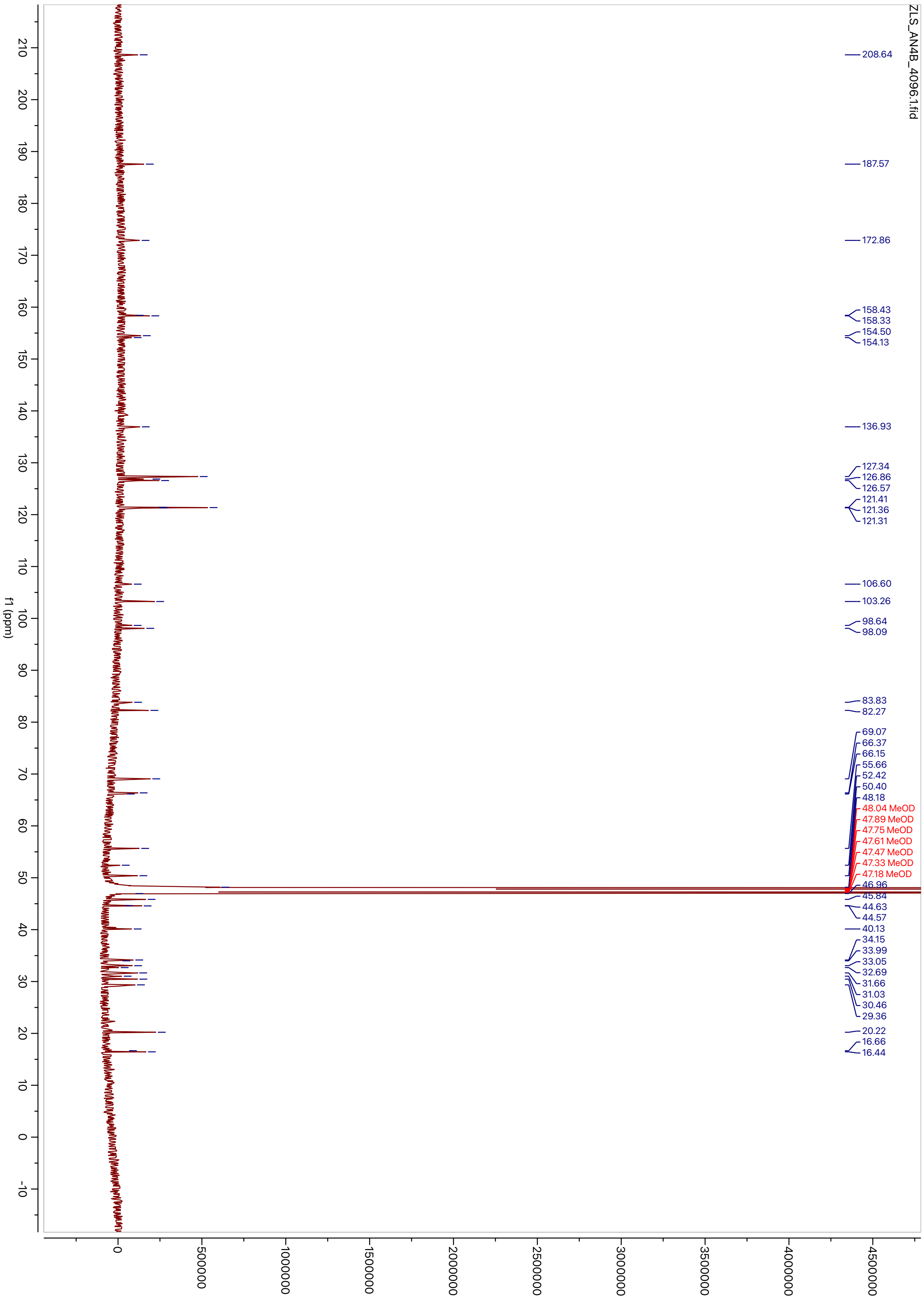

*S 31 NOESY NMR of AzoCort4β (400MHz, MeOD)*

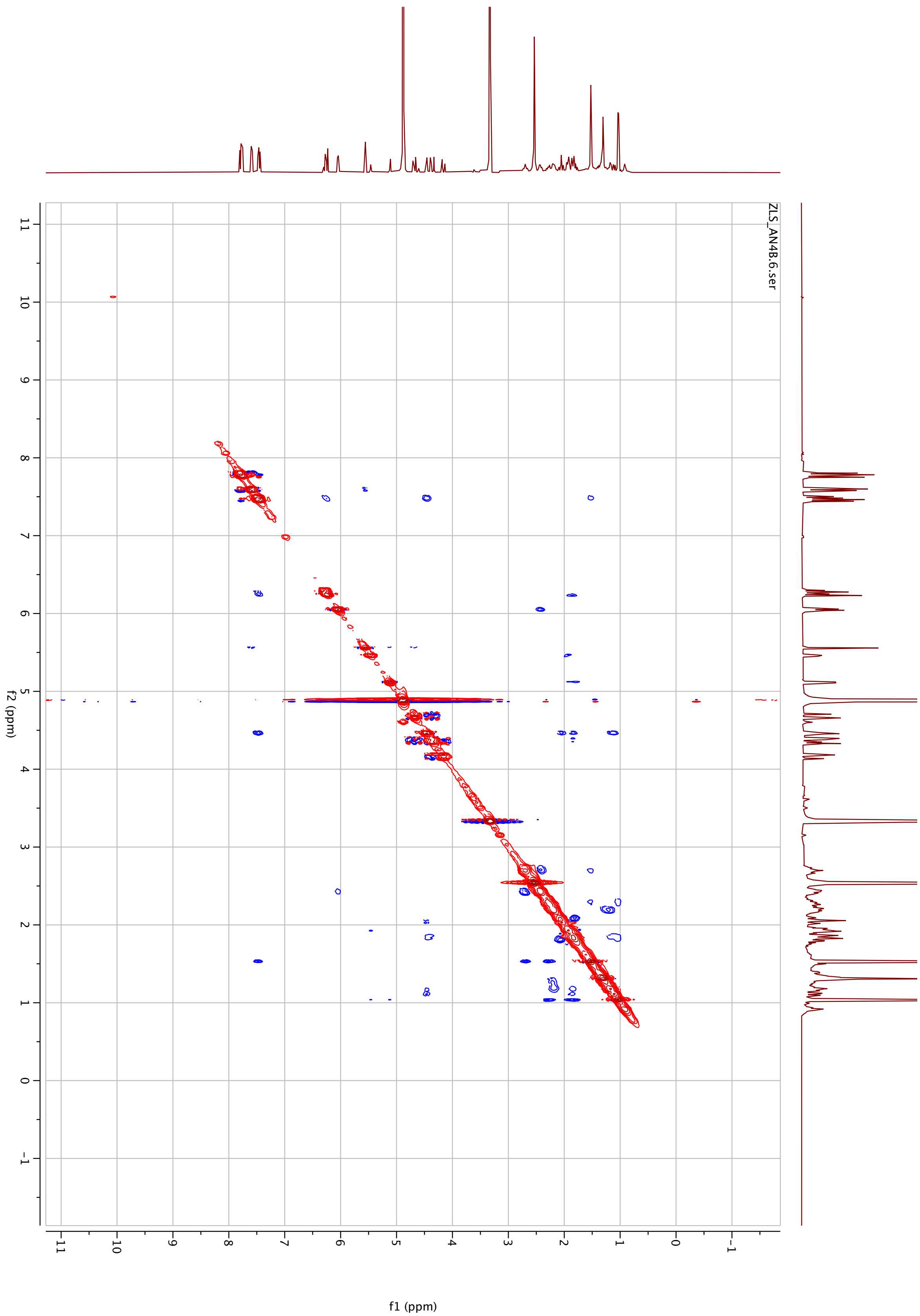

*S 32 1H NMR of AzoCort5 (400MHz, MeOD)*

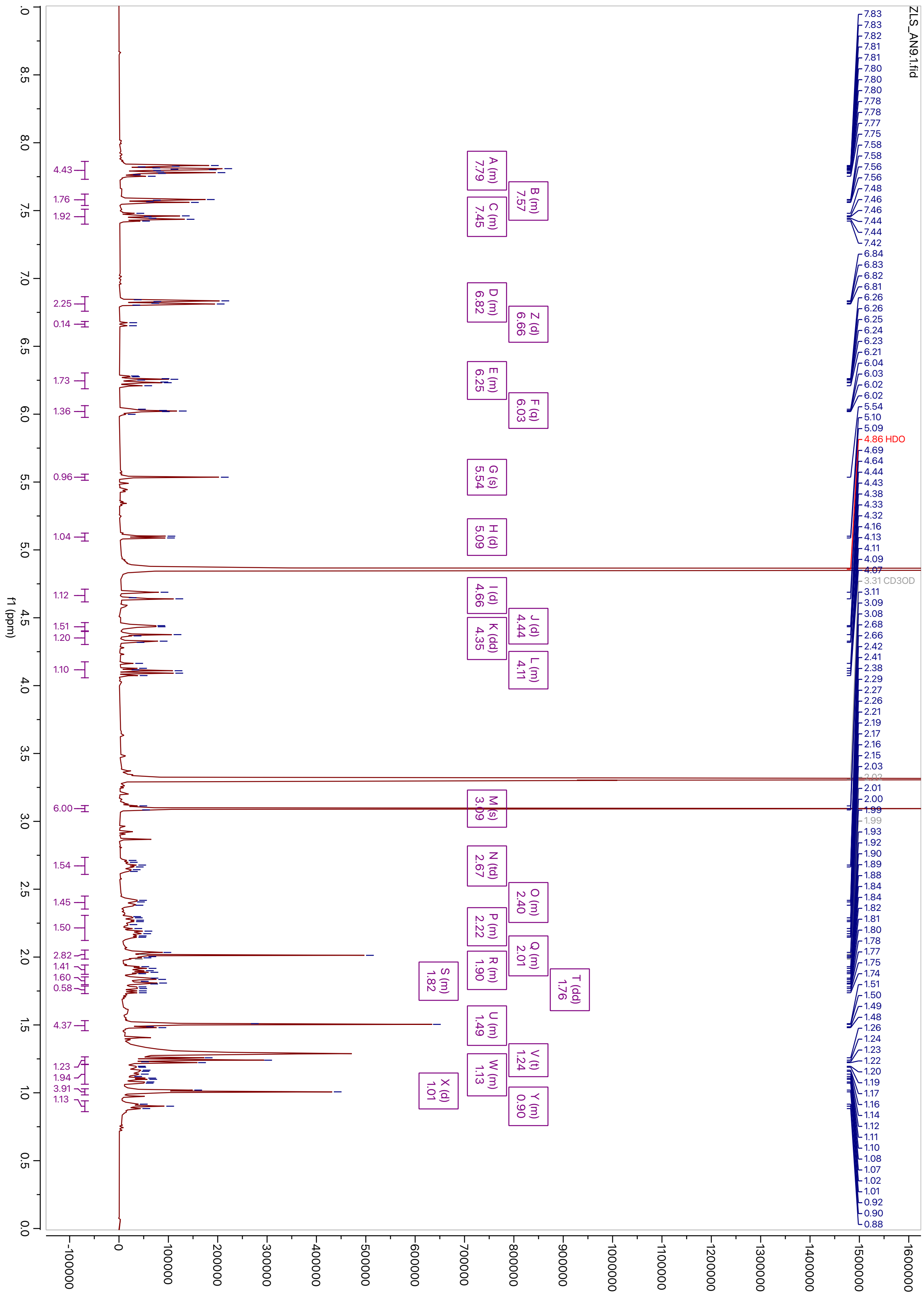

*S 33 13C NMR of AzoCort5 (151MHz, MeOD)*

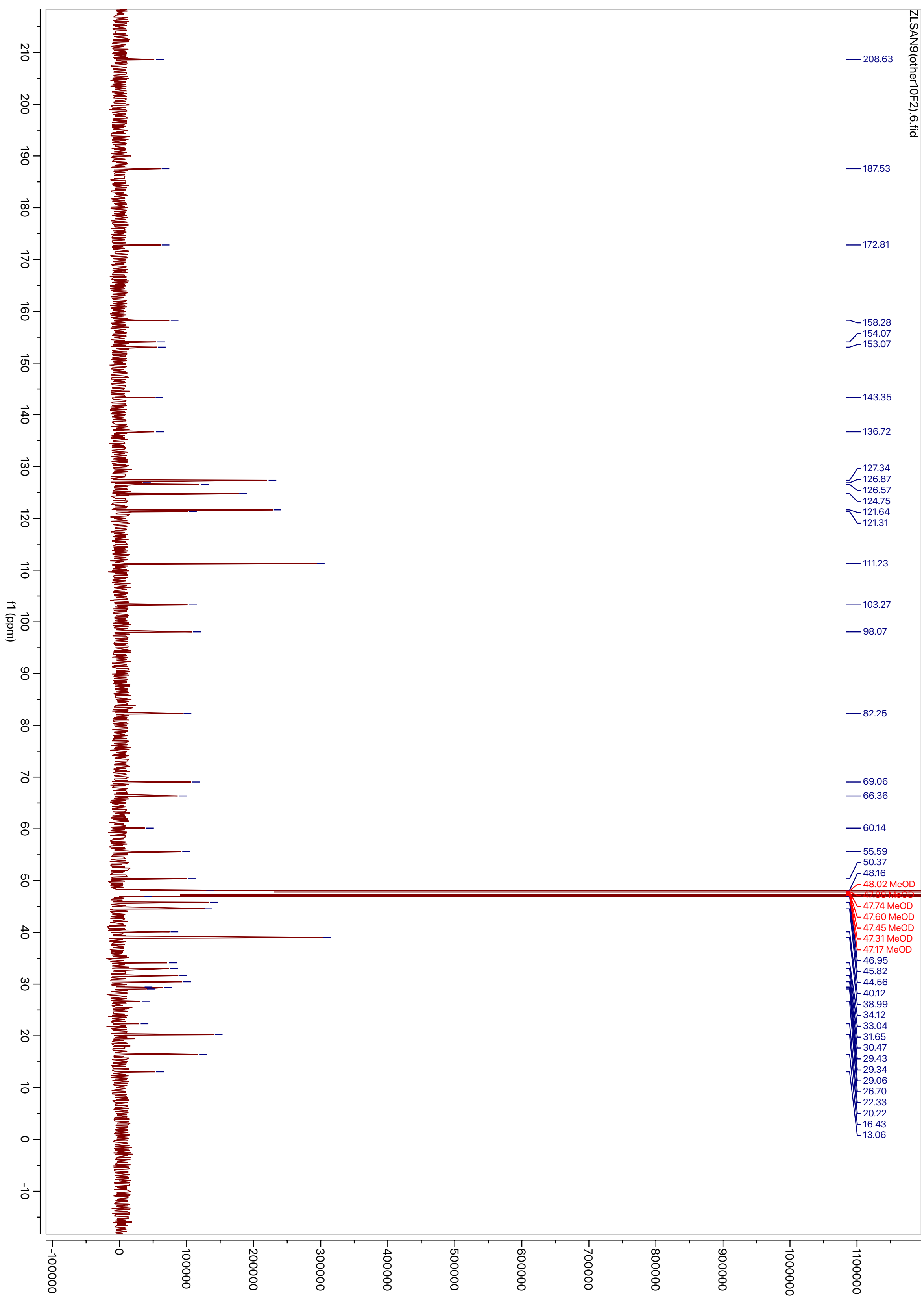

*S 34 HSQC NMR of AzoCort5 (600MHz, MeOD)*

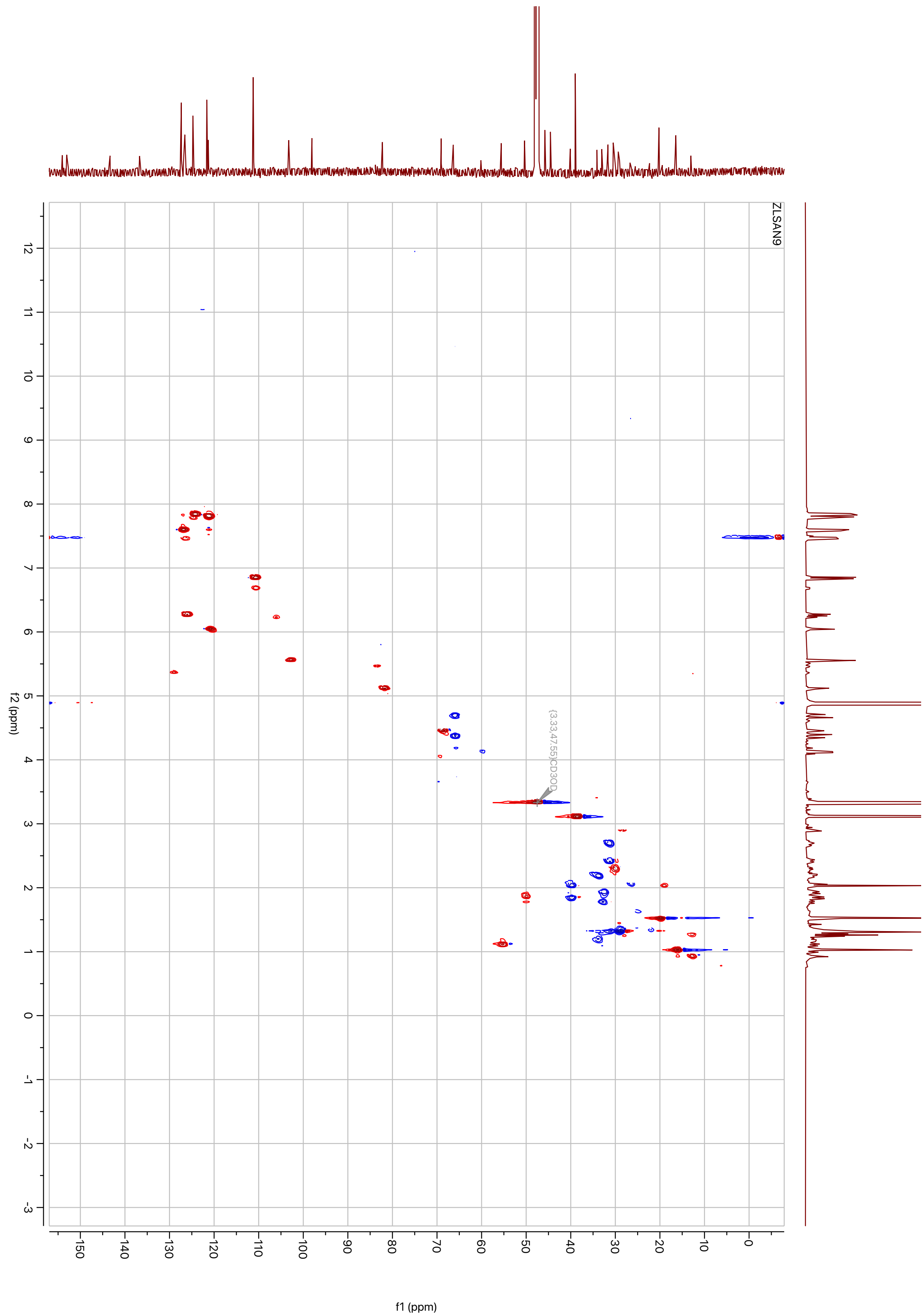

*S 35 NOESY NMR of AzoCort5 (600MHz, MeOD)*

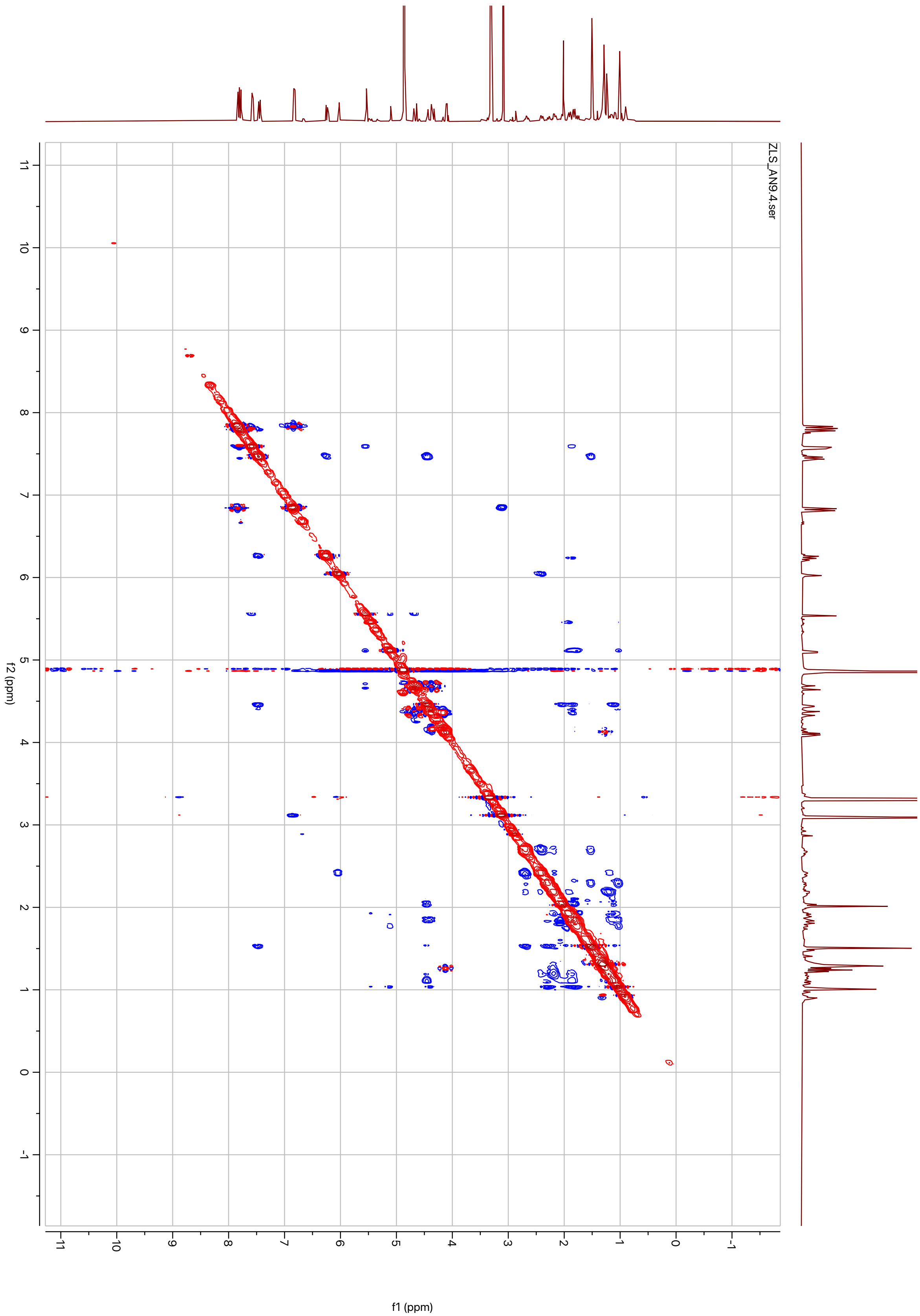

*S 36 1H NMR of AzoCort6 (400MHz, MeOD)*

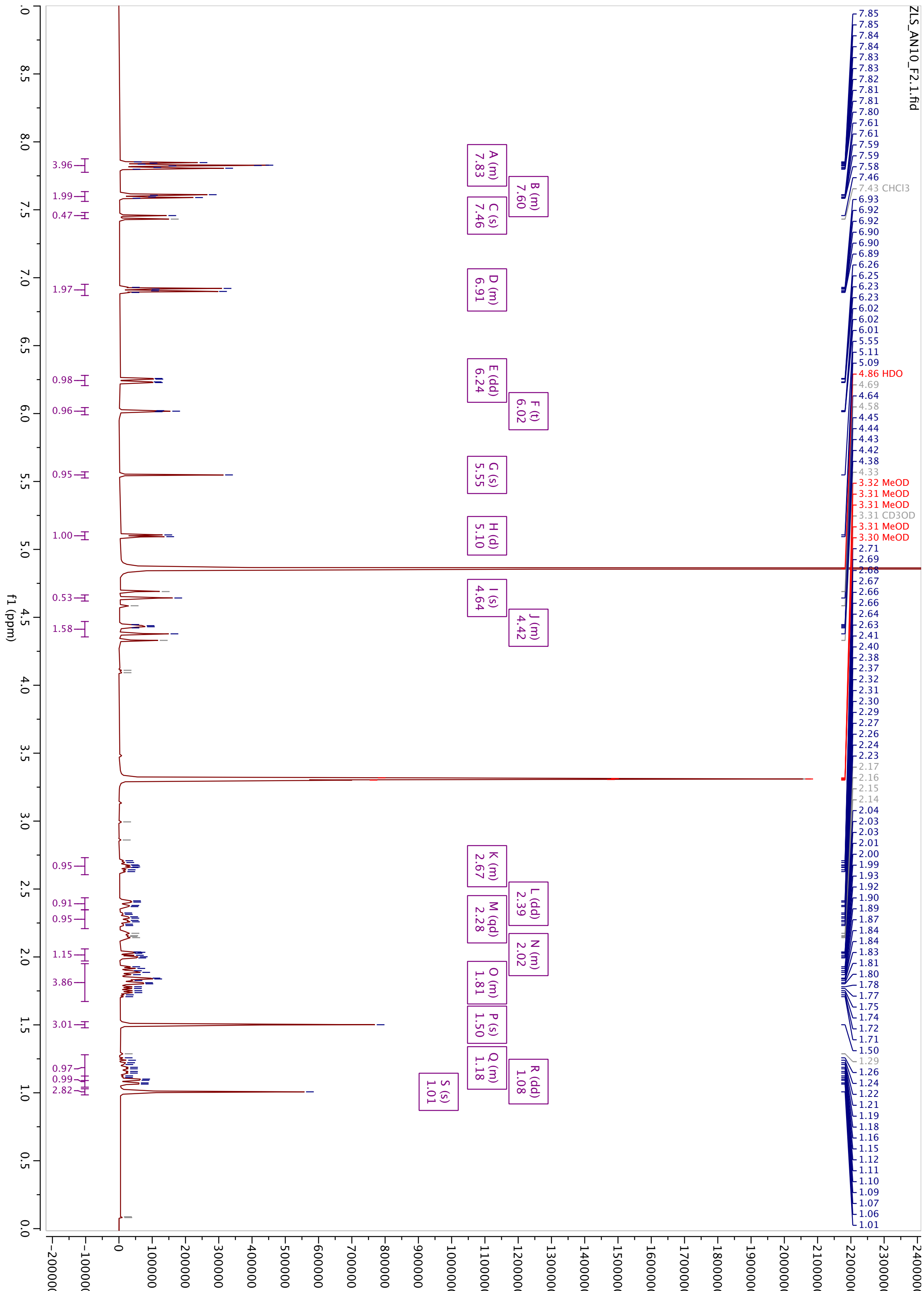

*S 37 13C NMR of AzoCort6 (151MHz, MeOD)*

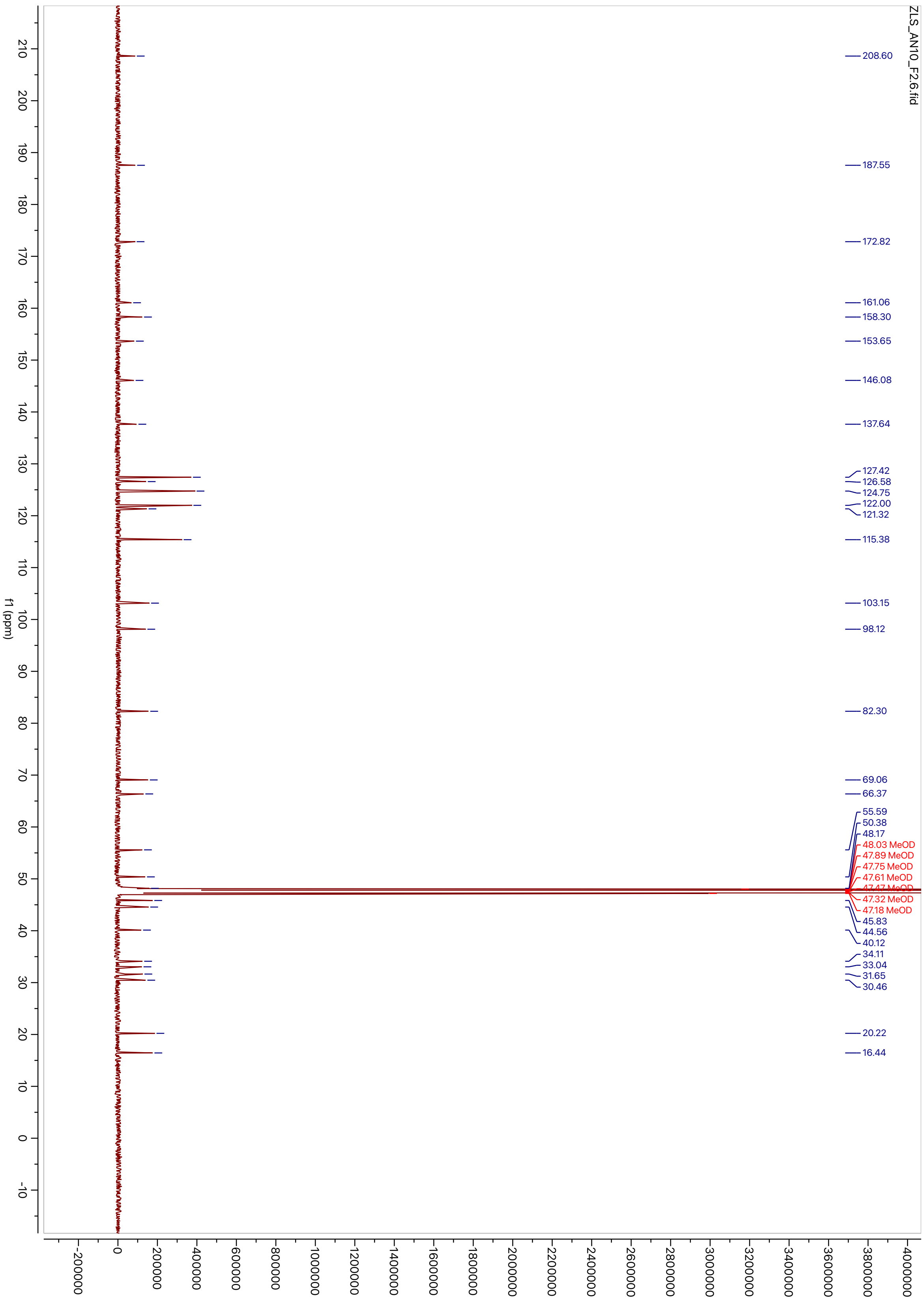

*S 38 HSQC NMR of AzoCort6 (600MHz, MeOD)*

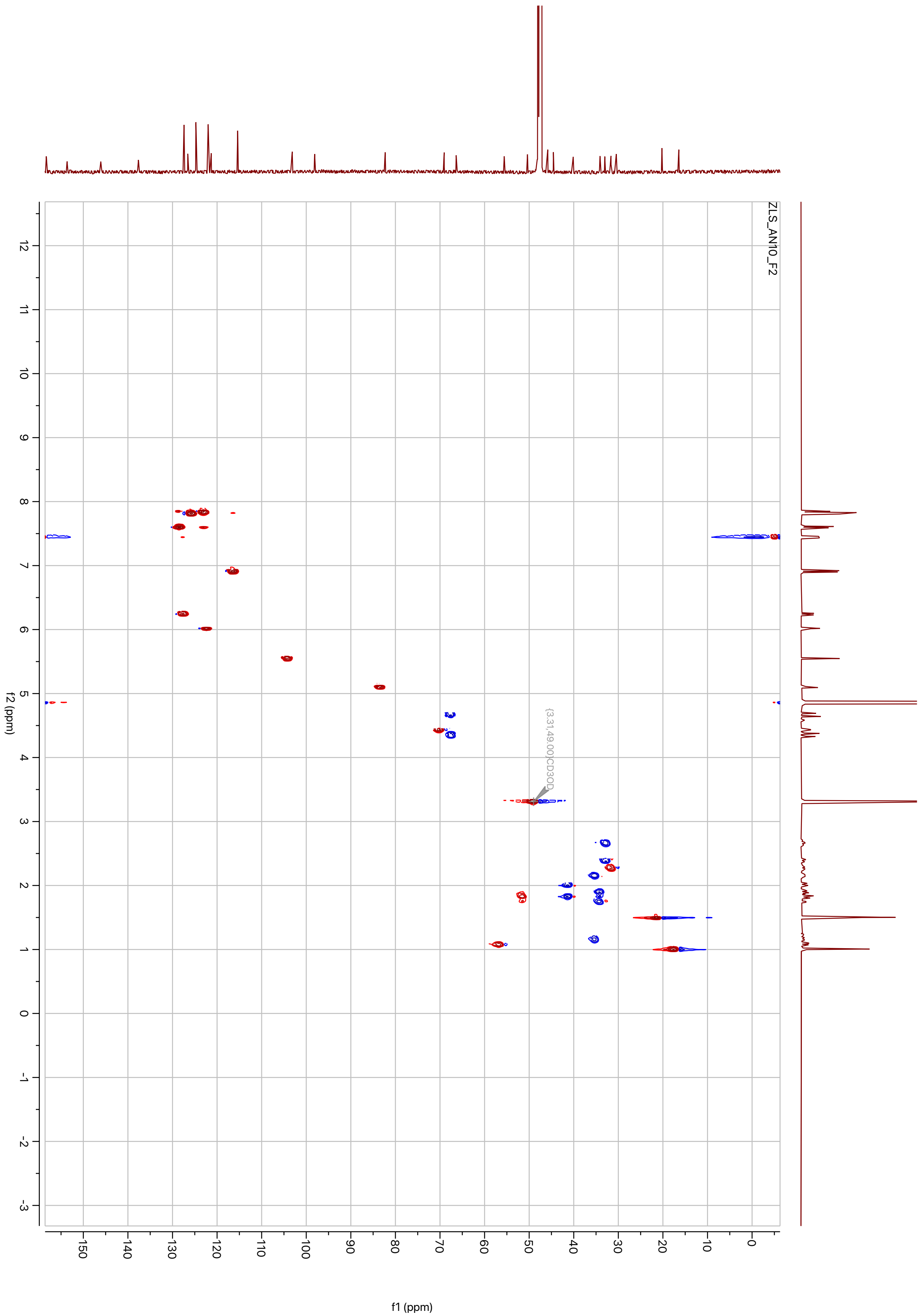

*S 39 NOESY NMR of AzoCort6 (600MHz, MeOD)*

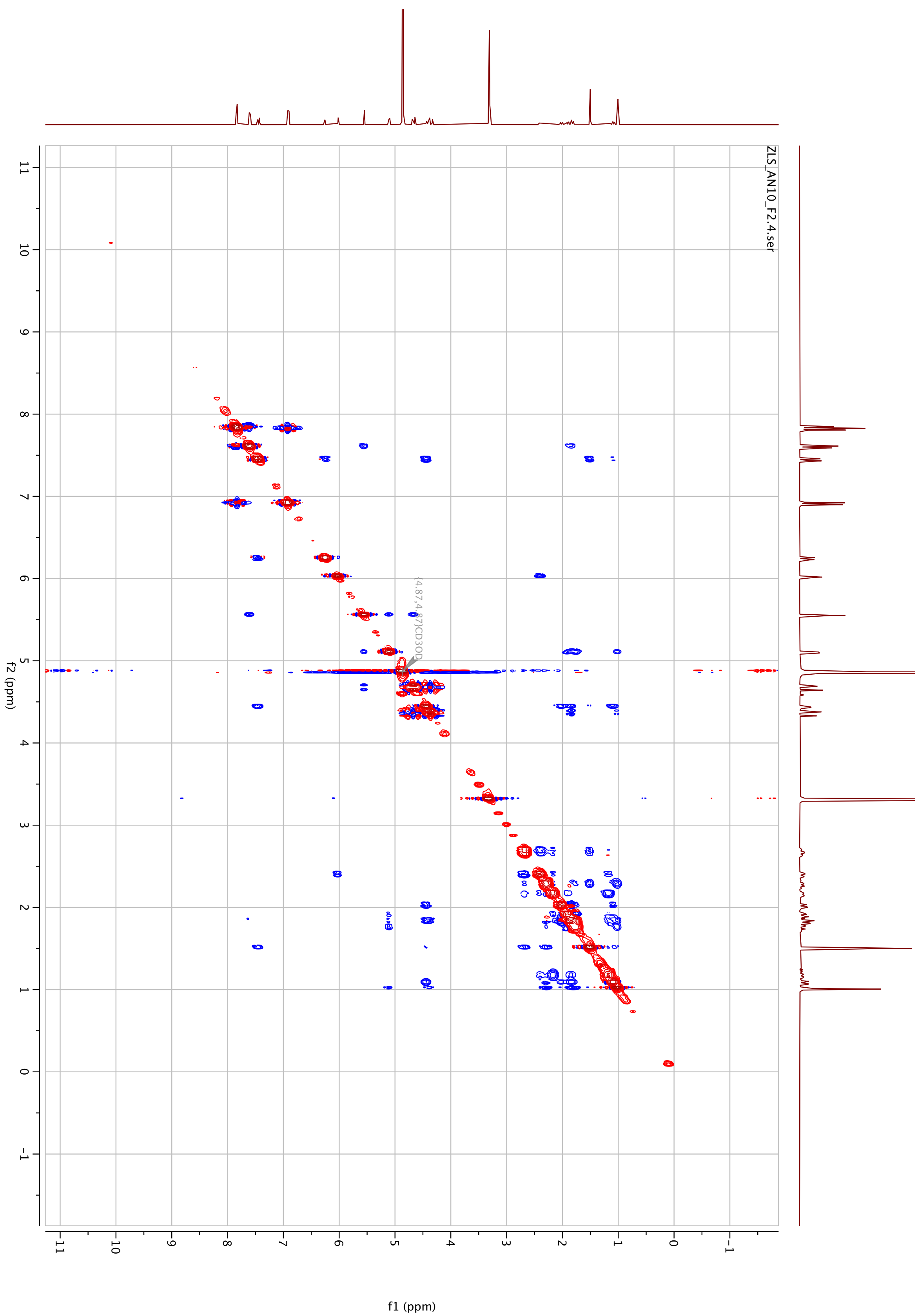

*S 40 1H NMR of AzoCort6β (400MHz, MeOD)*

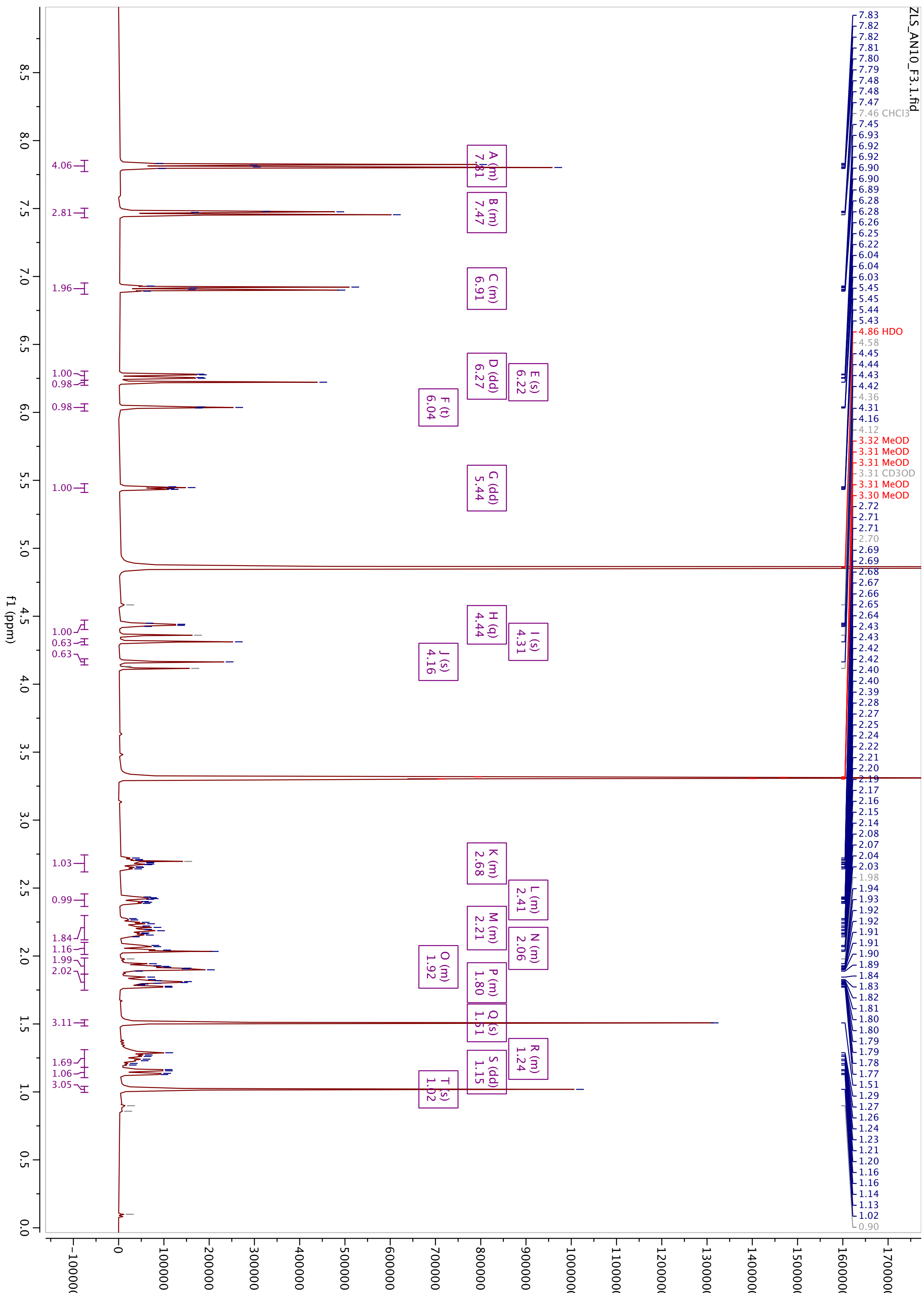

S 41 13C NMR of AzoCort6β (101MHz, MeOD)

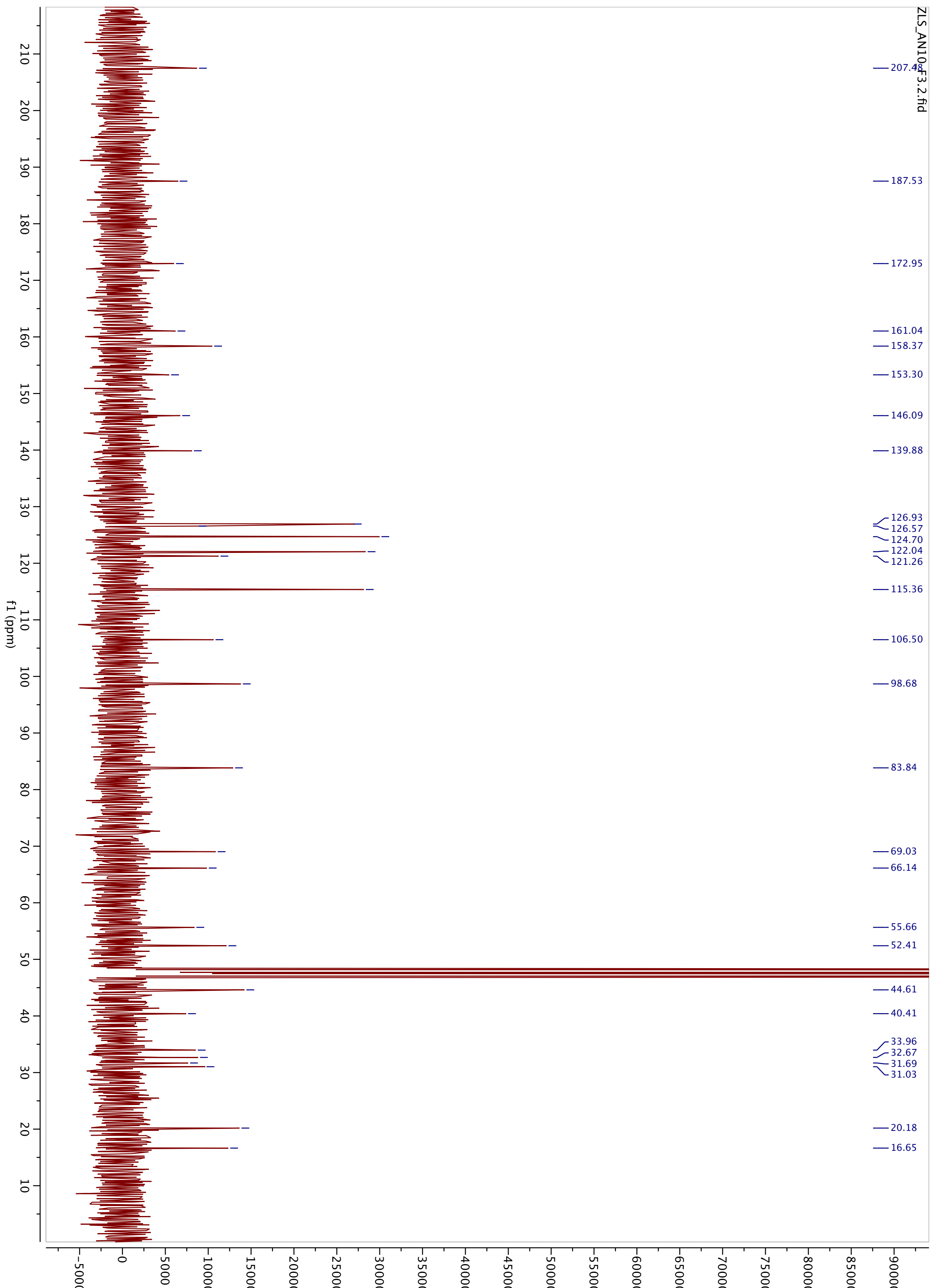

*S 42 NOESY NMR of AzoCort6$\beta$ (400MHz, MeOD)*

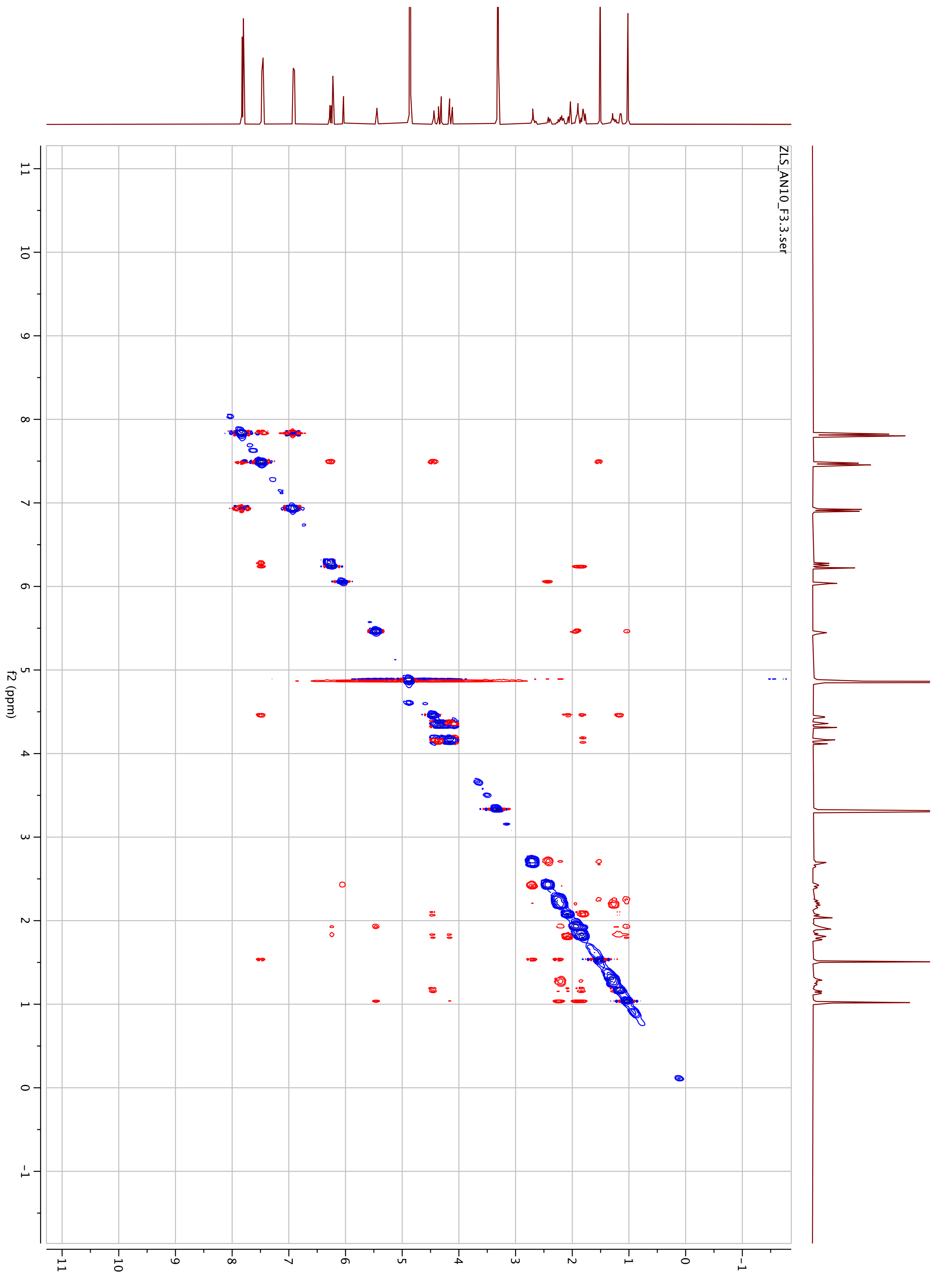

## LC-MS Purity Traces

All final compounds submitted for biological evaluation had a purity > 95%. Purity was determined via analytical LC-MS using an Agilent Poroshell 120 4 SB-C18 reverse phase column (3.0 x 50 mm) at 254nm. A linear gradient of 5-95% acetonitrile in water (0.1% formic acid) over 5 minutes followed by a 2-minute hold (flow rate of 0.5 mL/min) was used. Retention time and purity is calculated from the area of the UV trace at 254nm excluding the injection peak.

*S 43 AzoCort1 LC-MS Purity Trace*

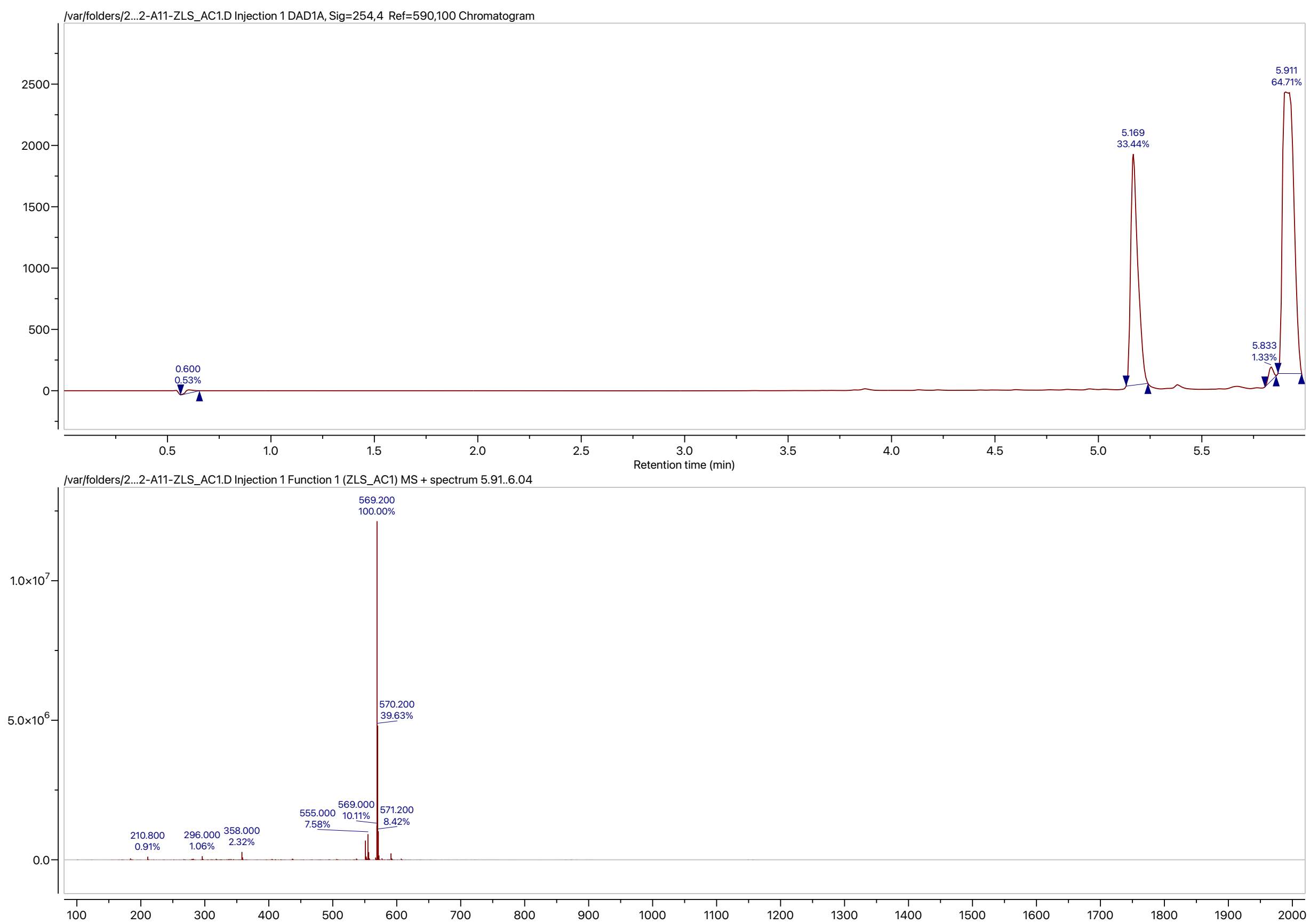


$t_R$= 5.17 + 5.91 min, m/z $[M+H]^+$569.200. Purity 98.7% (254nm)

*S 44 AzoCort2 LC-MS Purity Trace*

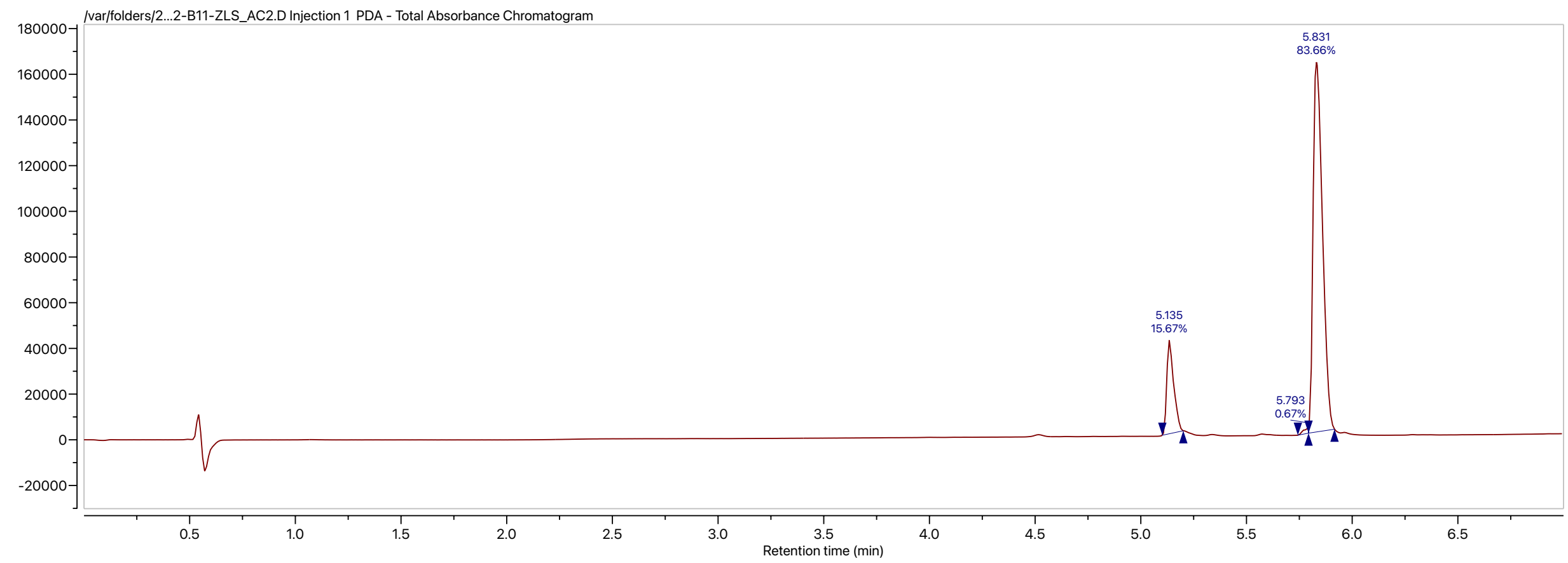


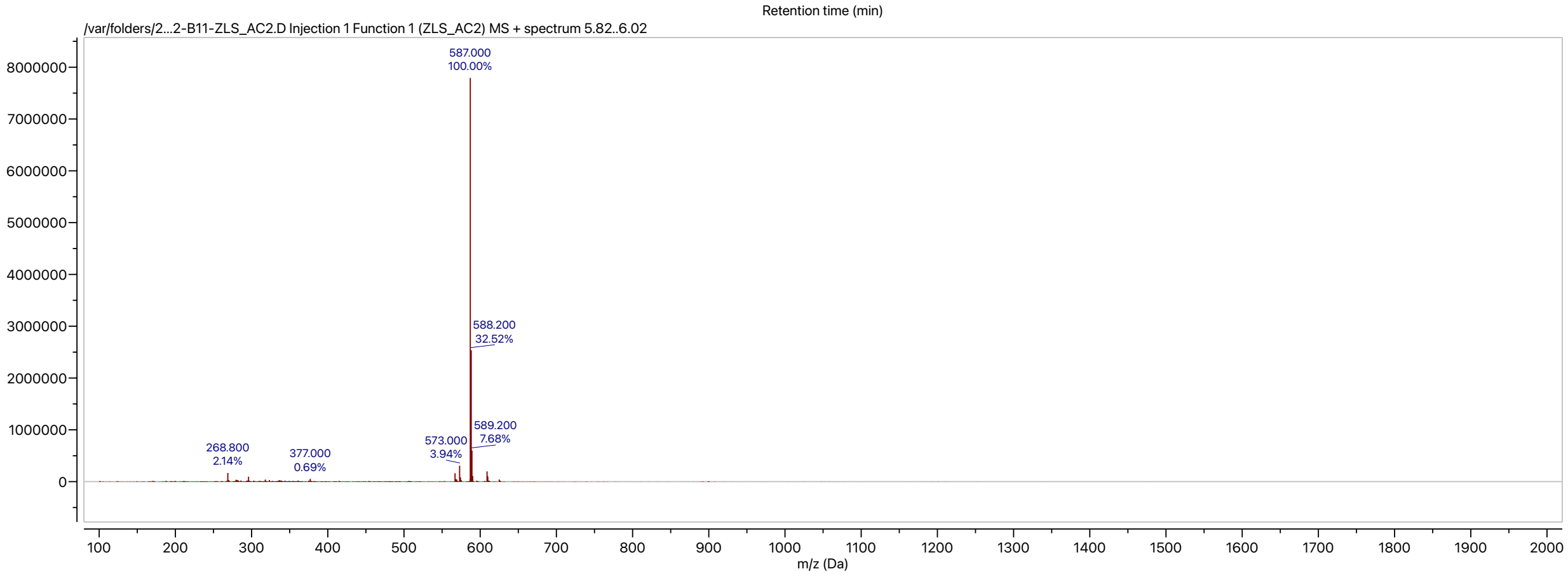


$t_R$= 5.14 + 5.83 min, m/z $[M+H]^+$ 587.000. Purity 99.3% (254nm)

*S 45 AzoCort3 LC-MS Purity Trace*

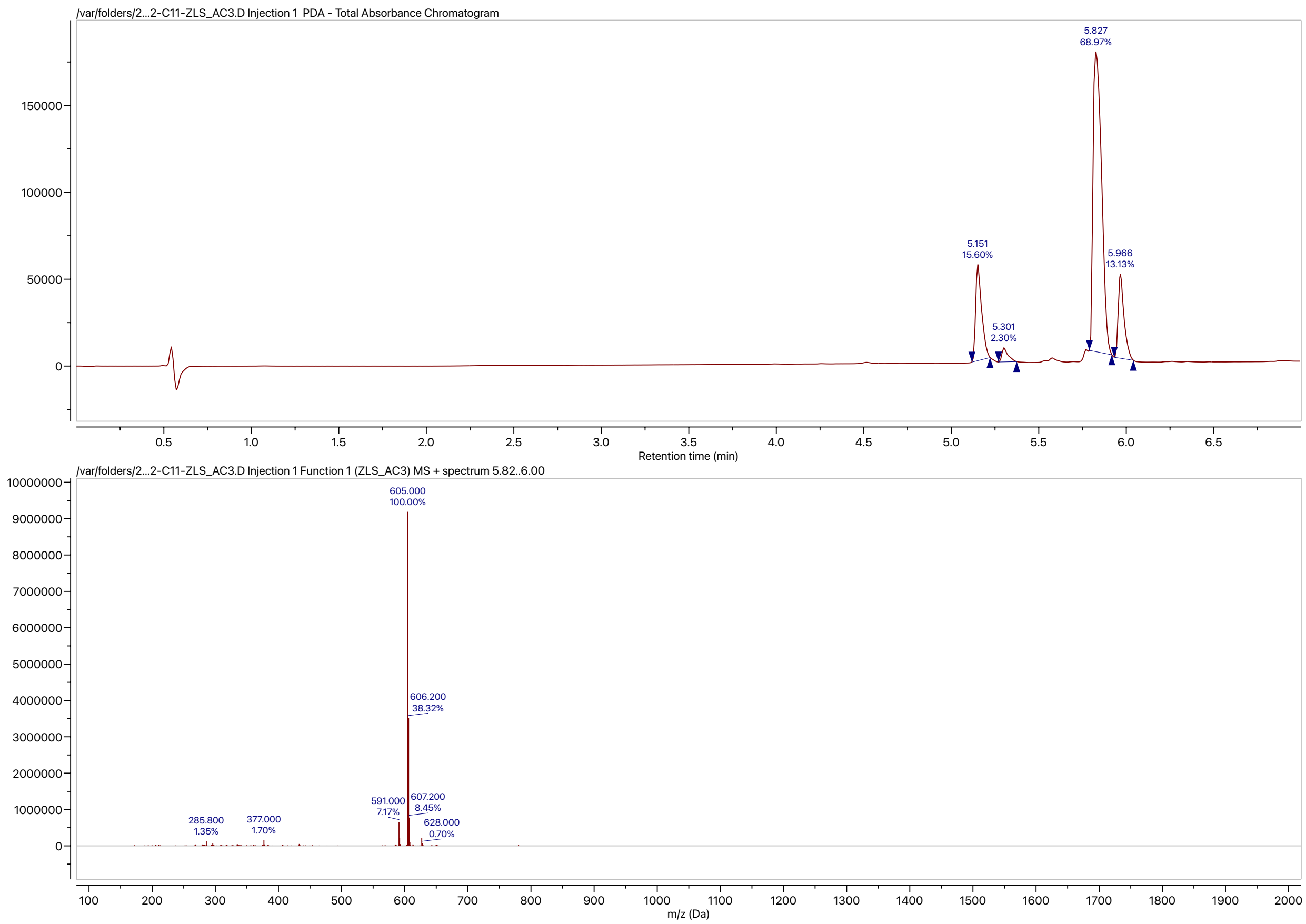


$t_R$= 5.15 + 5.83 min, m/z $[M+H]^+$ 605.000. Purity 84.6%, 15.4% AzoCort3 C22β (254nm)

*S 46 AzoCort4 LC-MS Purity Trace*

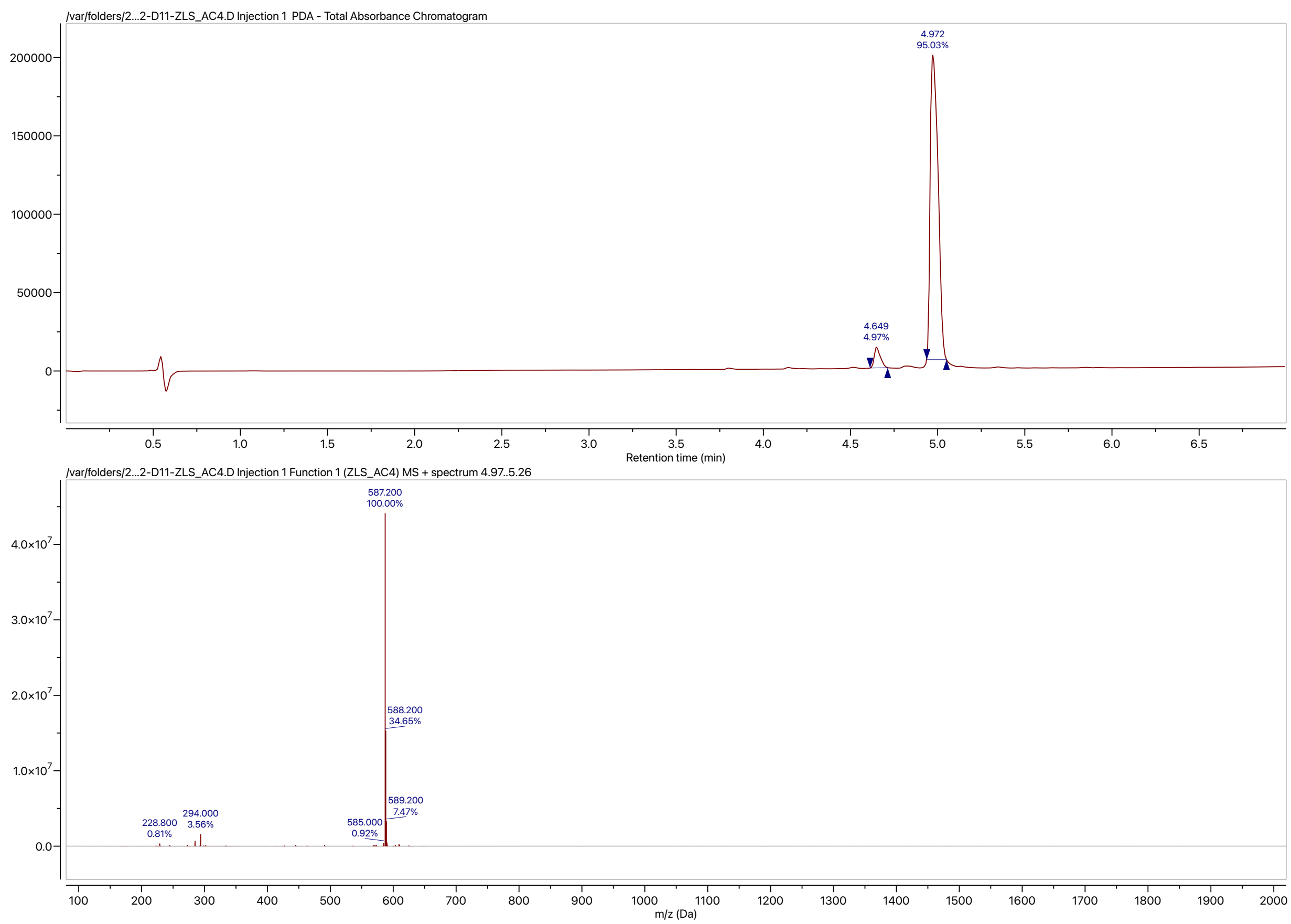


$t_R$= 4.97 min, m/z $[M+H]^+$ 587.200. Purity 95.0% (254nm)

*S 47 AzoCort5 LC-MS Purity Trace*

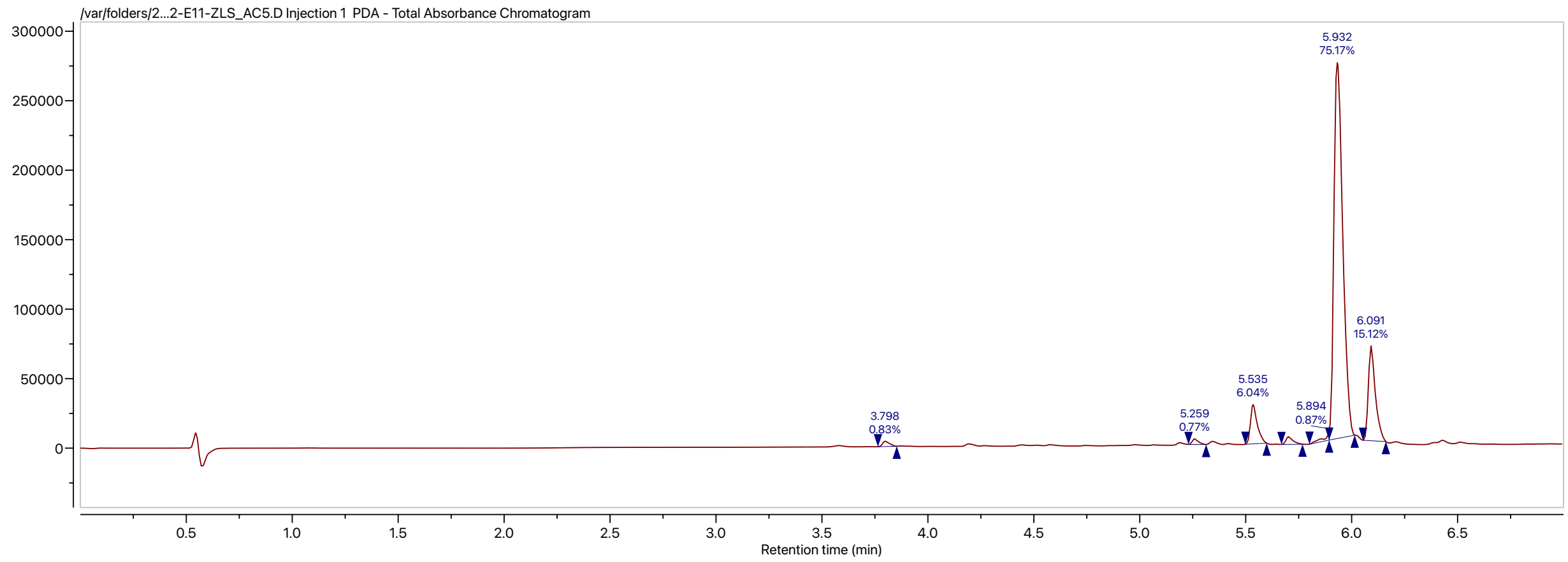


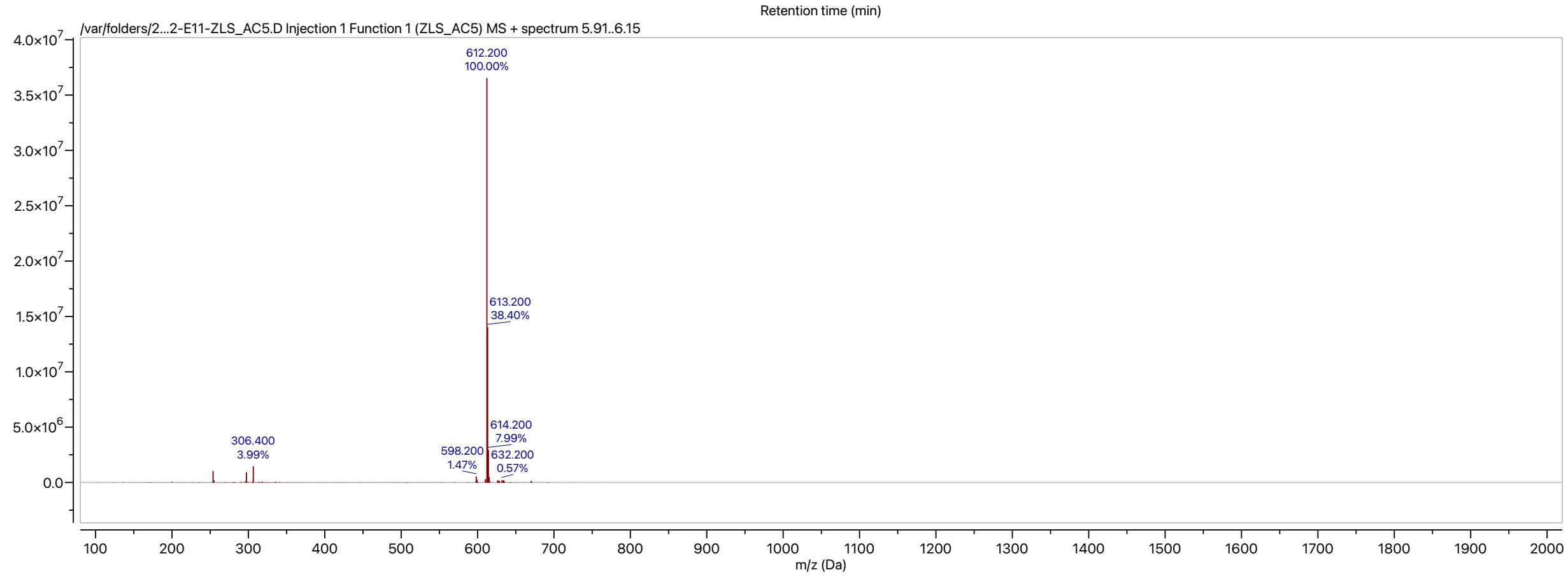


$t_R$= 5.93 + 6.09 min, m/z $[M+H]^+$ 612.200. Purity 90.3% (254nm)

*S 48 AzoCort6 LC-MS Purity Trace*

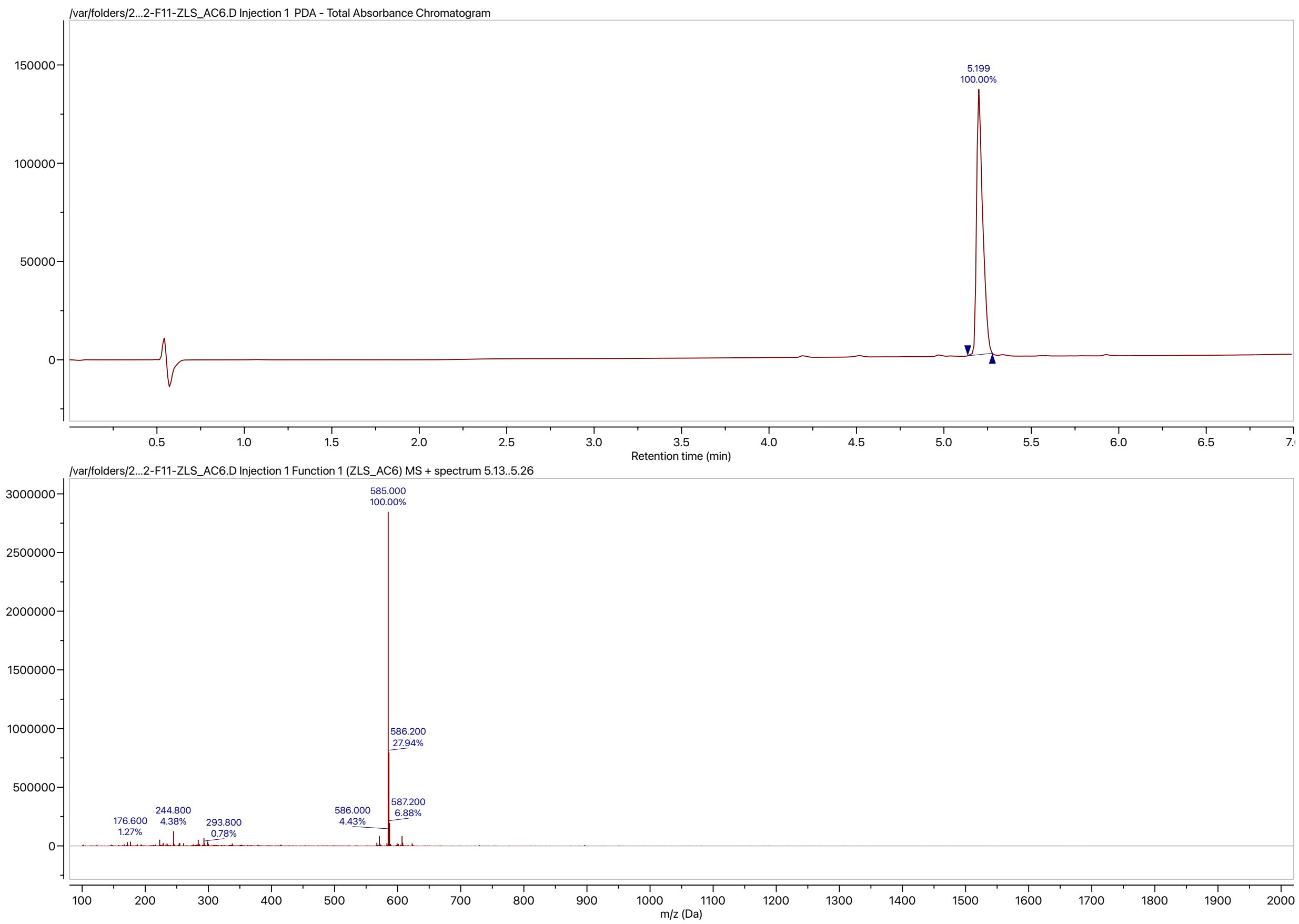


$t_R$= 5.20 min, m/z $[M+H]^+$ 585.000. Purity = 99.9% (254nm)

# UV-Vis Characterization

## Methods

All samples were in 100% DMSO at 50 μM. All experiments were performed at room temperature, except thermal relaxation which was kept at 37 °C using a circulating water bath. Wavelengths scans were performed using 5 min increments of irradiation. Thermal relaxation was performed from maximum cis enrichment and monitored with the noted wavelength. Photocycling was performed using the optimal wavelengths per compound, as represented by the color (360 nm & 440 nm for AzoCort1-3, 400 nm & 600 nm for AzoCort4, 360 nm & 625 nm for AzoCort5, and 380 nm & 600 nm for AzoCort6). Light sources were a monochromator (Cairn OptoScan with a 75W Xenon Arc Lamp) or LEDs (Roithner LaserTechnik LED600-03, B5-433-B525, VL430-5-15, VL420-5-15, VL410-5-15, RLS-UV400, VL390-5-15, B5M-447-380S, XSL-370-5E, or XSL-360-5E) and absorbance was measured using an Agilent Cary 60 UV-Vis Spectrometer. Data was processed in GraphPad Prism (version 10.5)[3]. The photostationary state was estimated as relative trans:cis ratios from UV-vis absorbance changes at $\lambda_{max}$, assuming the dark-adapted spectrum corresponds predominantly to the thermodynamically stable trans isomer.

# Wavelength Scan, Thermal Relaxation, & Photocycling Spectra

*S 49 AzoCort1 Photocharacterization*

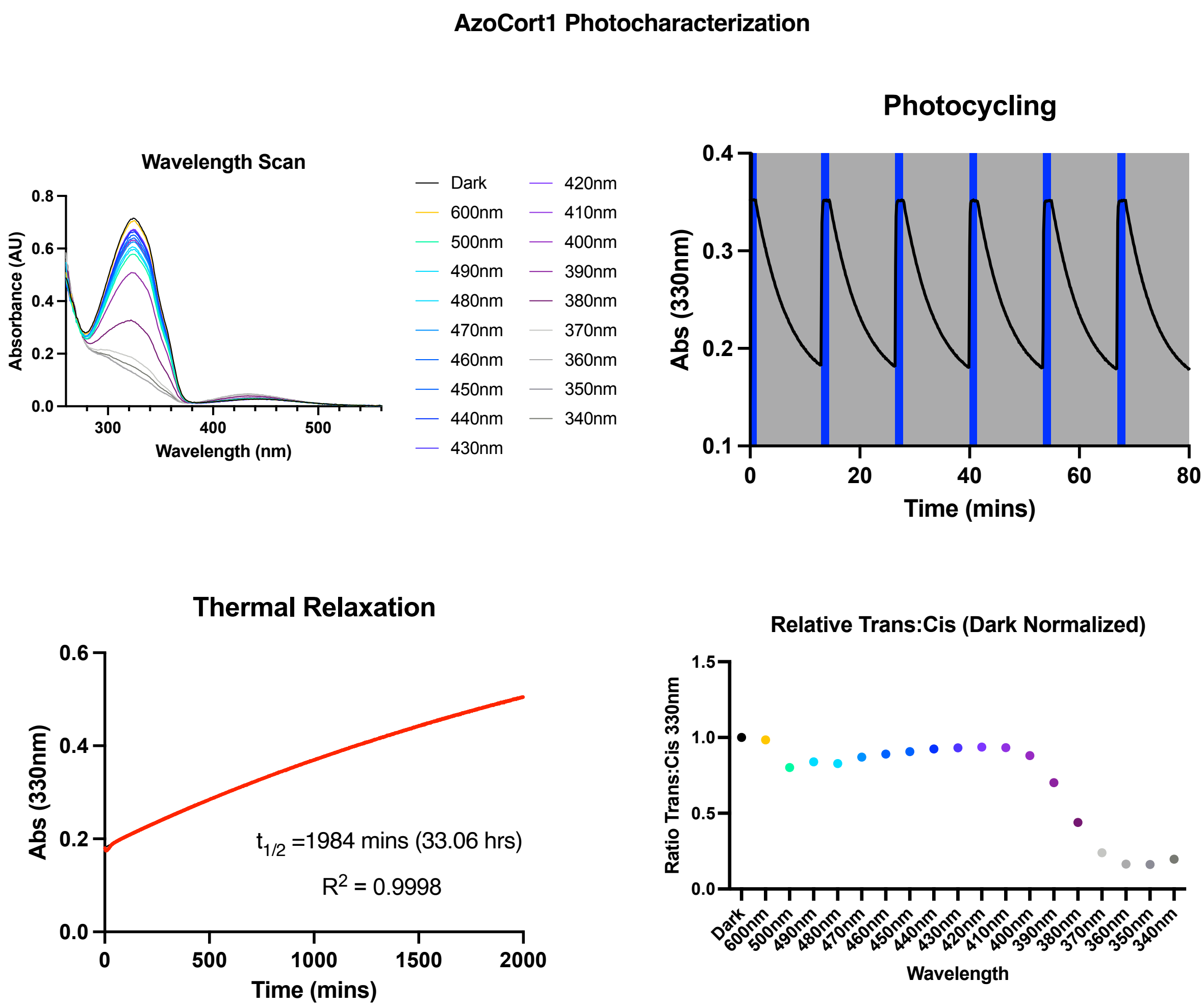

*S 50 AzoCort2 Photocharacterization*

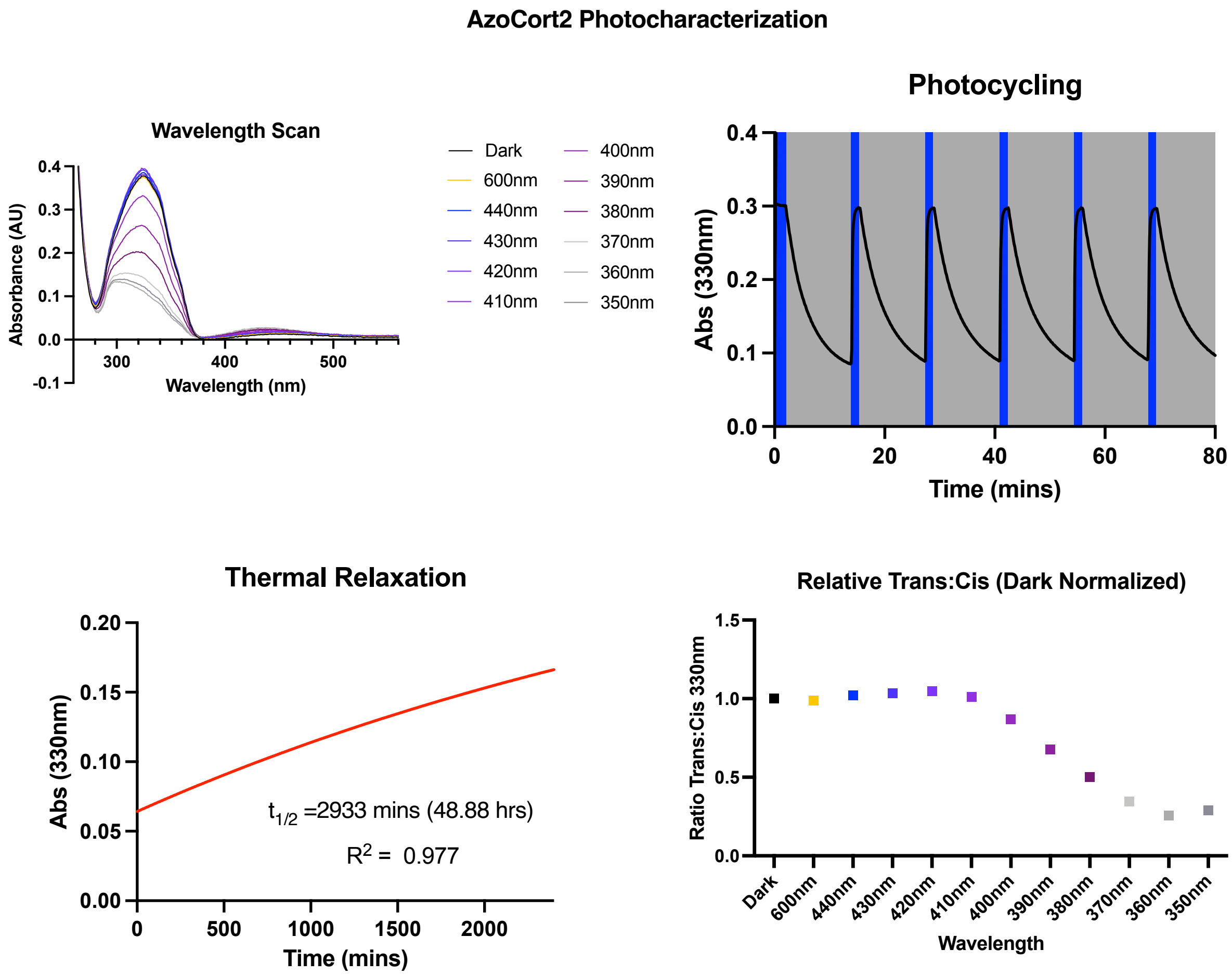

*S 51 AzoCort3 Photocharacterization*

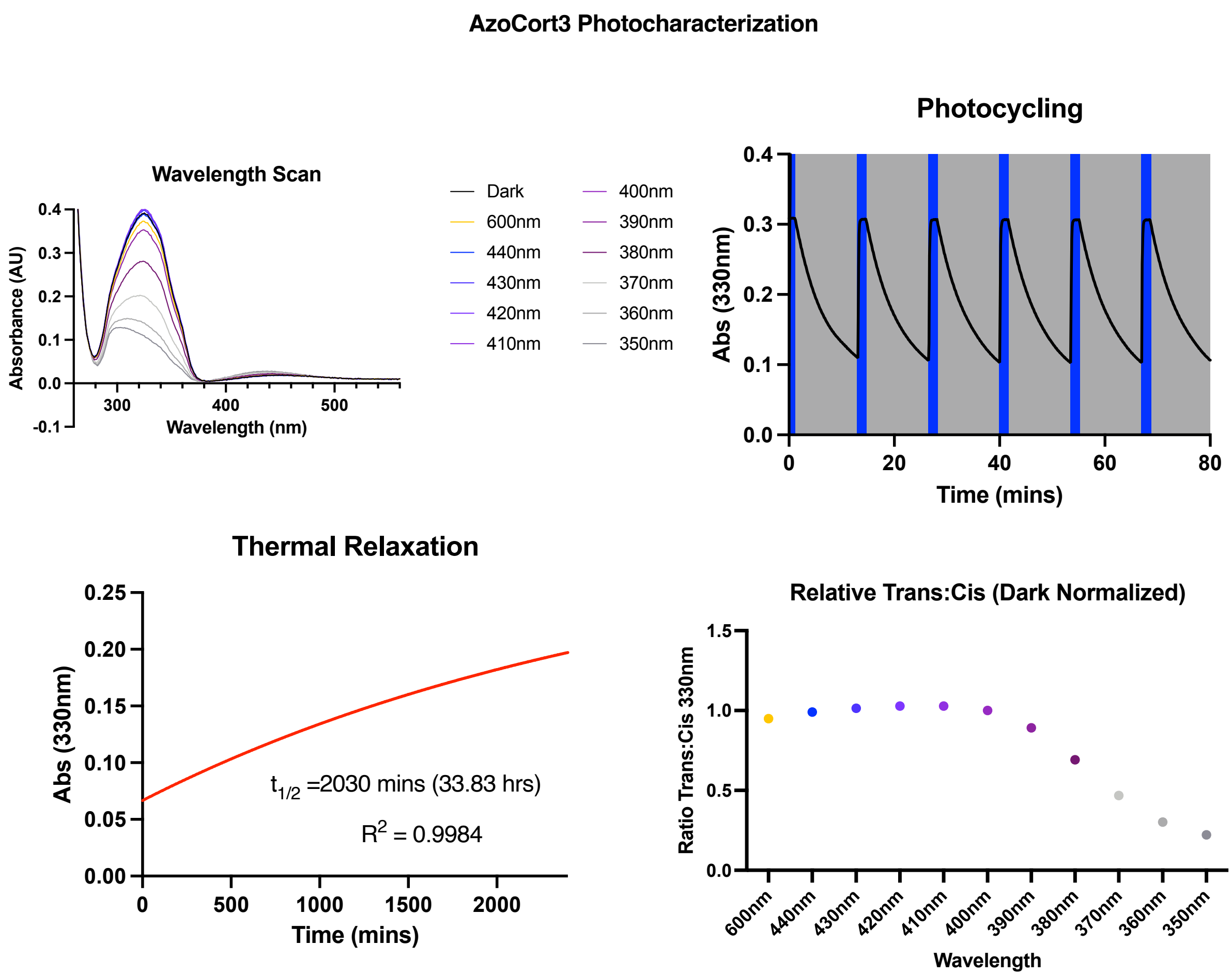

*S 52 AzoCort4 Photocharacterization*

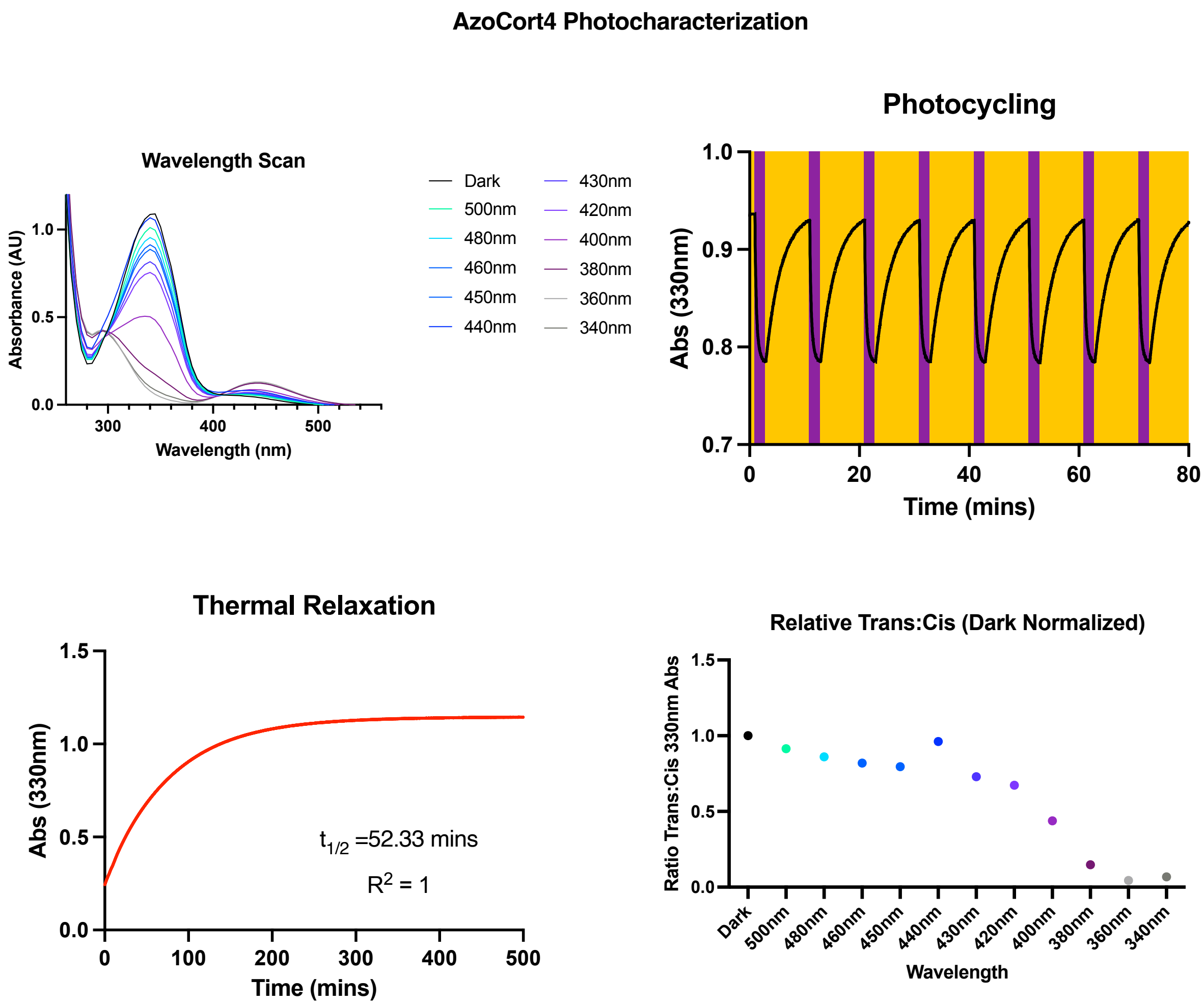

*S 53 AzoCort5 Photocharacterization*

**AzoCort5 Photocharacterization**

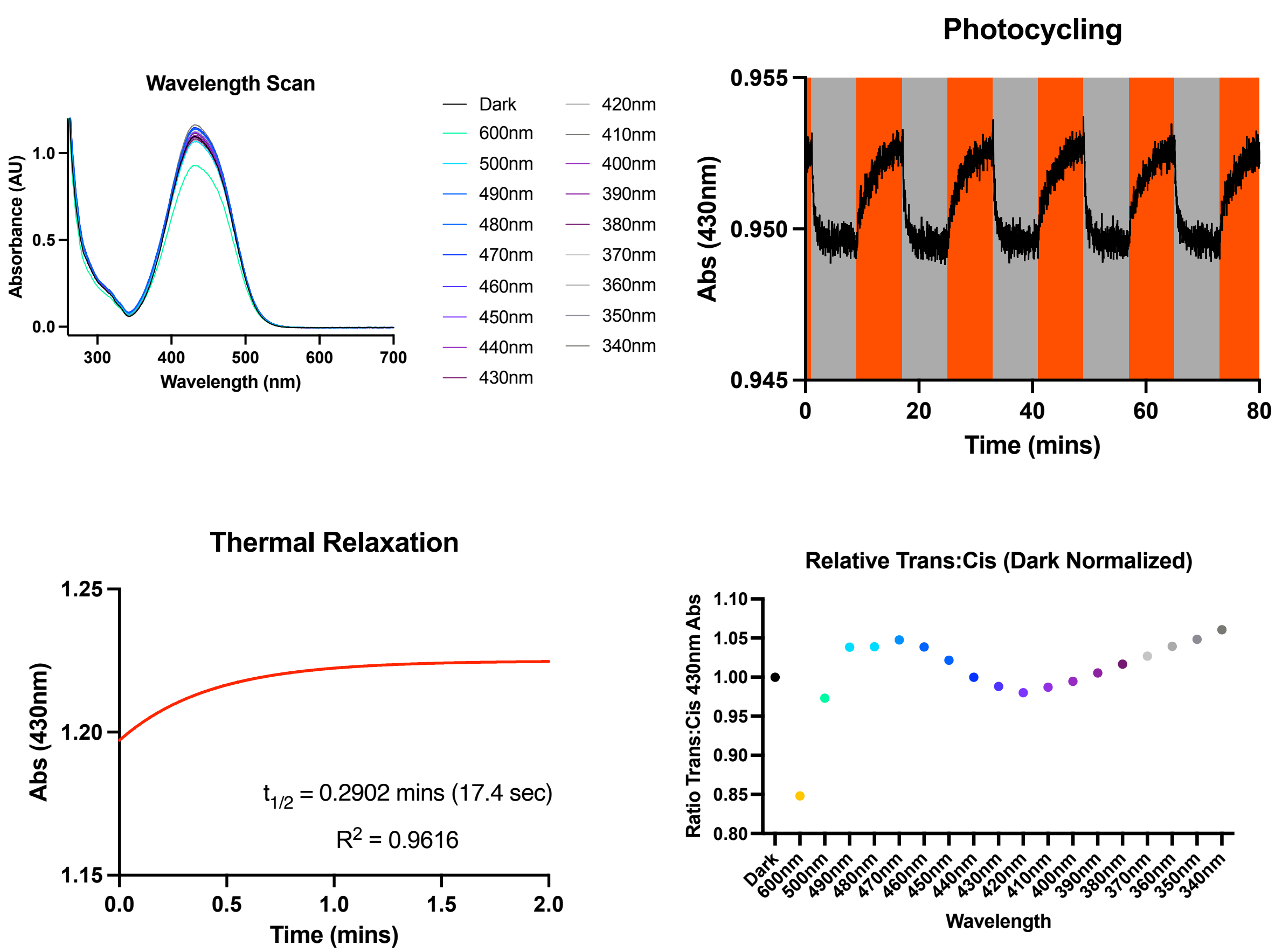

*S 54 AzoCort6 Photocharacterization*

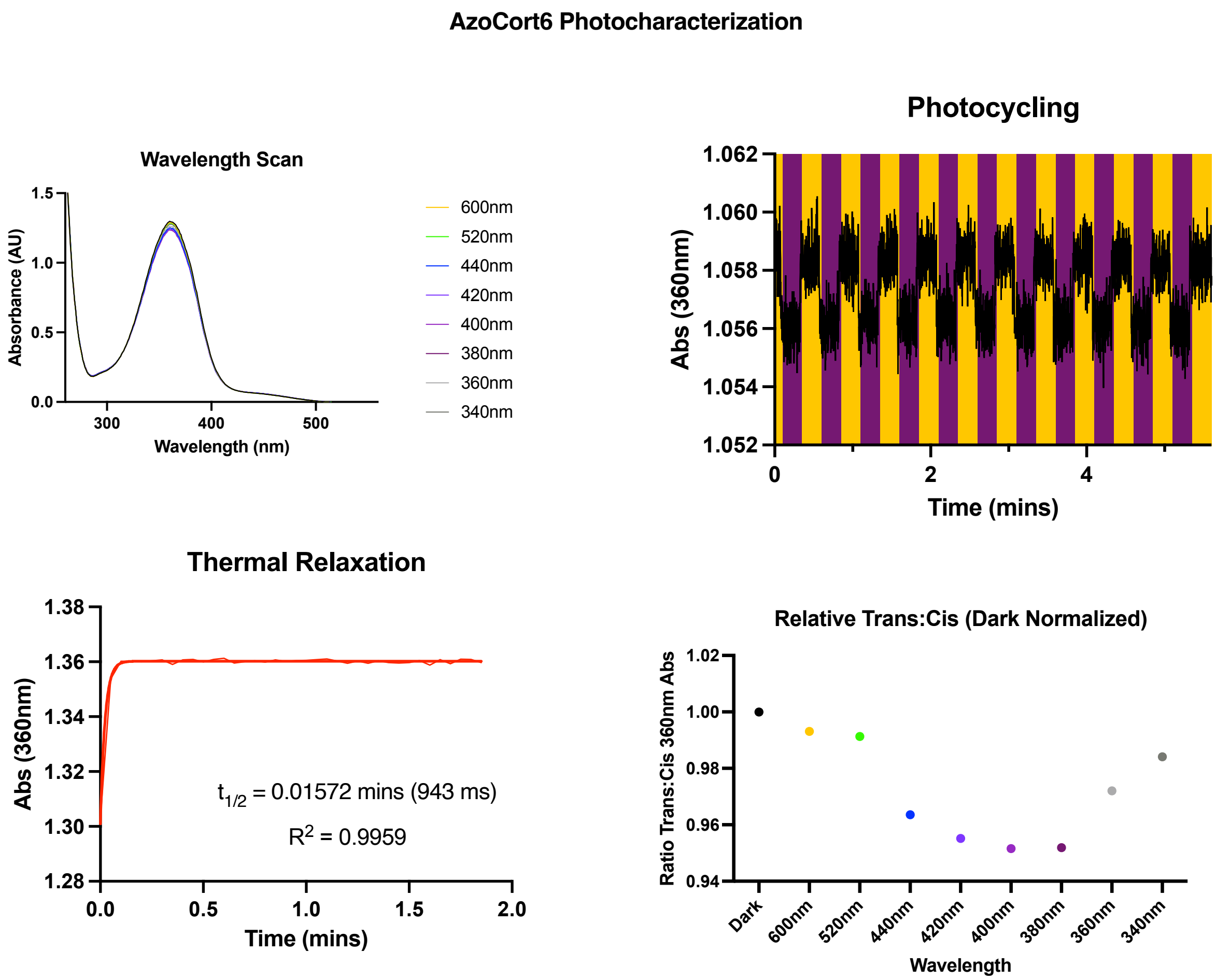

# Computational Methods

## Molecular Docking

3D molecular docking was performed using Maestro version 13.4.134, MMshare Version 6.0.134, Release 2022-4, Platform Darwin-x86_64.[6] All simulations were performed with a pH of 7.4 assumed, as well as all water 2 Å or further from the binding pose removed. The following nodes were used throughout the generation of the docking poses: Protein Preparation, LigPrep, Receptor Grid Generation, and Ligand Docking. Following the receptor grid generation, the co-crystallized ligand was extracted and redocked as a positive control. Docking was run using flexible XP parameters and the OPLS_2005 force field. Reported data are glide scores, where the minimum score for reporting is -6, with the more negative score representing higher predictive binding affinity. Interaction analyses were performed using PLIP (protein–protein interactions to the protein–ligand interaction profiler)[7] and visualized in PyMol[8] (version 2.5.5).

## Data Processing

### Immunofluorescence Data Processing

Images were processed using a Fiji macro (ImageJ2 Version 2.14.0)[2]. The script applies Huang threshold to generate filters for nuclear (DAPI=1) and cytosolic (DAPI=0). The raw mean grey values are then counted for each image and filter. Using GraphPad Prism (version 10.5)[3]. The cytosolic and nuclear fractions are calculated based on the total per image. The script for Fiji is as follows:

```
// ====== USER SETTINGS ======

parentDir = getDirectory("Choose parent folder");

outputFile = parentDir + "Combined_Results.csv";

// ==========================


// Clear existing Results table

if (isOpen("Results")) run("Clear Results");


// Set measurement parameters

run("Set Measurements...", "mean integrated limit display redirect=None decimal=3");
```

```
// Process all subfolders recursively
processFolder(parentDir);

// Save combined results
saveAs("Results", outputFile);
print("✅ All done! Combined results saved to: " + outputFile);


// ===========================
// FUNCTIONS
// ===========================

function processFolder(folder) {
  list = getFileList(folder);
  for (i = 0; i < list.length; i++) {
    path = folder + list[i];
    if (File.isDirectory(path)) {
      processFolder(path); // recurse into subfolder
    } else if (endsWith(path, "d1.tif") || endsWith(path, "d1.TIF")) {
      prefix = replace(list[i], "d1.tif", "");
      prefix = replace(prefix, "d1.TIF", "");
      dapiPath = folder + prefix + "d0.tif";
      if (!File.exists(dapiPath)) dapiPath = folder + prefix + "d0.TIF";
      if (File.exists(dapiPath)) analyzePair(dapiPath, path);
      else print("⚠️ No matching DAPI for " + list[i]);
```

```
    }
  }
}

function analyzePair(dapiPath, gfpPath) {
  dapiName = File.getName(dapiPath);
  gfpName = File.getName(gfpPath);

  // Open images
  open(dapiPath);
  open(gfpPath);

  // --- Nuclear mask (DAPI) ---
  selectImage(dapiName);
  run("Duplicate...", "title=[" + dapiName + "-nuc]");
  nucMask = dapiName + "-nuc";
  selectImage(nucMask);
  setAutoThreshold("Huang dark no-reset");
  setOption("BlackBackground", true);
  run("Convert to Mask");
  run("16-bit");
  run("Subtract...", "value=254");  // 0/1 mask

  // --- Cytoplasmic mask (inverse of Nuclear) ---
  selectImage(dapiName);
```

```
run("Duplicate...", "title=[" + dapiName + "-cyto]");
cytoMask = dapiName + "-cyto";
selectImage(cytoMask);
setAutoThreshold("Huang dark no-reset");
setOption("BlackBackground", true);
run("Convert to Mask");
run("16-bit");
run("Subtract...", "value=254"); // 0/1 mask
run("Invert");              // invert to get cytoplasm

// --- Multiply for Nuclear ---
imageCalculator("Multiply create", gfpName, nucMask);
selectImage("Result of " + gfpName);
setAutoThreshold("Huang dark no-reset");
run("Measure");
nRow = nResults() - 1;
setResult("Signal", nRow, "Nuclear");
close(getTitle());

// --- Multiply for Cytoplasmic ---
imageCalculator("Multiply create", gfpName, cytoMask);
selectImage("Result of " + gfpName);
setAutoThreshold("Huang dark no-reset");
run("Measure");
cRow = nResults() - 1;
setResult("Signal", cRow, "Cytoplasmic");
```

```
    close(getTitle());

    // --- Add identifiers ---
    startRow = nResults() - 2;
    for (r = startRow; r < nResults(); r++) {
        setResult("File", r, gfpName);
        setResult("Folder", r, File.getParent(gfpPath));
    }
    updateResults();

    // Close all images
    close("*");
    print("✅ Analyzed: " + gfpName);
}
```

# Supplementary Tables

TABLE S1 HPLC PURIFICATION METHODS .......... 90
TABLE S2 ANTIBODY SPECIFICATIONS & USAGE .......... 90
TABLE S3 AZOCORT GRE $EC_{50}$ VALUES .......... 91

## HPLC Purification Methods

*Table S1 HPLC Purification Methods*

| **Method ID** | **Column & Gradient (% MeCN in $H_2O$)** |
|---|---|
| **A** | Agilent Poroshell 120 4 SB-C18 21.2 x 150mm<br>0-1 min 50%,1-4mins 50-75%, 4-9 mins 75-90%, 9-10mins 90-95%<br>OR<br>Agilent Pursuit XRs 5 C18 21.2 x 250mm<br>0-1 min 50-75%, 1-3min 75-85%, 3-6min 85-95%, 6-10 min 95% ($t_r$ = 6.601 (C22$\alpha$) and 7.309 (C22$\beta$)) |
| **B** | Agilent Pursuit XRs 5 C18 21.2 x 250mm<br>0-1 min 80-85%, 1-5min 85-90%, 5-10min 90-95% |
| **C** | Agilent Pursuit XRs 5 C18 21.2 x 250mm<br>0-1 min 80-87%, 1-5min 87-90%, 5-10min 90-95% |
| **D** | Agilent Poroshell 120 4 SB-C18 21.2 x 150mm<br>0-1 min 50-65%, 1-8mins 65-85% |
| **E** | Agilent Poroshell 120 4 SB-C18 21.2 x 150mm<br>0-7 min 50-95%, 7-8mins 95% |

## Antibody Specifications

*Table S2 Antibody Specifications & Usage*

| **Antibody** | **Manufacturer** | **Catalog #** | **Species** | **Dilution Used** |
|---|---|---|---|---|
| Glucocorticoid Receptor (D6H2L) | Cell Signaling | 12041S | Rabbit (Monoclonal) | 1:1000 (WB)<br>1:500 (IF) |
| P**hospho-Glucocorticoid Receptor (Ser203)** | Sigma Aldrich | SAB4504585 | Rabbit | 1:1000 (WB) |
| Phospho-Glucocorticoid | Cell Signaling | 4161S | Rabbit | 1:1000 (WB) |

| | | | | |
|---|---|---|---|---|
| Receptor (Ser211) | | | | |
| P**hospho-Glucocorticoid Receptor (Ser226)** | Cell Signaling | 97285T | Rabbit (Monoclonal) | 1:1000 (WB) |
| GAPDH (D4C6R) | Cell Signaling | 97166S | Rabbit (Monoclonal) | 1:1000 (WB) |
| Anti-Rb AlexaFluor 488 | ThermoFisher | A-11008 | Goat | 1:500 (IF) |
| Anti-rabbit IgG, HRP-linked Antibody | Cell Signaling | 7074S | Goat | 1:5000 (WB) |
| Anti-mouse IgG, HRP-linked Antibody | Cell Signaling | 7076S | Horse | 1:5000 (WB) |

## AzoCort GRE Luciferase $EC_{50}$ Values

*Table S3 AzoCort GRE $EC_{50}$ Values*

| **Compound (360nm)** | **360nm $EC_{50}$** | **Dark $EC_{50}$** |
|---|---|---|
| AzoCort1 | 0.01103 | 0.4008 |
| AzoCort2 | 0.007452 | 0.02144 |
| AzoCort3 | 0.454 | 0.09389 |
| AzoCort4 | 0.001367 | 0.00001784 |
| AzoCort5 | 162.8 | 652.8 |
| AzoCort6 | n.d. | n.d. |

## References


[1] L. Van Moortel, J. Thommis, B. Maertens, A. Staes, D. Clarisse, D. De Sutter, C. Libert, O. C. Meijer, S. Eyckerman, K. Gevaert, K. De Bosscher, "Novel assays monitoring direct glucocorticoid receptor protein activity exhibit high predictive power for ligand activity on endogenous gene targets" *Biomedicine and Pharmacotherapy* 2022, *152*, DOI 10.1016/j.biopha.2022.113218.

[2] J. Schindelin, I. Arganda-Carreras, E. Frise, V. Kaynig, M. Longair, T. Pietzsch, S. Preibisch, C. Rueden, S. Saalfeld, B. Schmid, J. Y. Tinevez, D. J. White, V. Hartenstein, K. Eliceiri, P. Tomancak, A. Cardona, 2012, DOI: 10.1038/nmeth.2019.

[3], GraphPad Prism, version 10.6, GraphPad Software, Boston, MA, USA, 2023.

[4] GelAnalyzer, version 23.1.1, István Lázár, Budapest, Hungary, 2023.

[5] C. W. Chu, L. Stricker, T. M. Kirse, M. Hayduk, B. J. Ravoo, "Light-Responsive Arylazopyrazole Gelators: From Organic to Aqueous Media and from Supramolecular to Dynamic Covalent Chemistry" *Chemistry - A European Journal* 2019, *25*, 6131–6140.

[6] Schrödinger Release 2022-4: Maestro, Glide, Schrödinger, LLC, New York, NY, 2022.

[7] P. Schake, S. N. Bolz, K. Linnemann, M. Schroeder, "PLIP 2025: introducing protein–protein interactions to the protein–ligand interaction profiler" *Nucleic Acids Res* **2025**, *53*, W463–W465.

[8] Schrödinger LLC, *The PyMOL Molecular Graphics System, Version 2.5.5*, **2015**.
